# Multiscale Theoretical Calculations Empower Robust Electric Double Layer Toward Highly Reversible Zinc Anode

**DOI:** 10.1007/s40820-025-01915-w

**Published:** 2025-12-25

**Authors:** Yufan Xia, Zhen Luo, Shuang Chen, Yang Xiang, Gao Weng, Hongge Pan, Ben Bin Xu, Mi Yan, Yinzhu Jiang

**Affiliations:** 1 School of Materials Science and Engineering, Zhejiang University, Hangzhou, 310027 People’s Republic of China; 2 Future Science Research Institute, ZJU-Hangzhou Global Scientific and Technological Innovation Center, Zhejiang University, Hangzhou, 311215 People’s Republic of China; 3 Institute of Science and Technology for New Energy, Xi’an Technological University, Xi’an, 710021 People’s Republic of China; 4 Mechanical and Construction Engineering, Faculty of Engineering and Environment, Northumbria University, Newcastle Upon Tyne, NE1 8ST UK

**Keywords:** Zn anode, Theoretical calculations, Electric double layers, Aqueous rechargeable zinc batteries

## Abstract

The electric double layer (EDL) at the electrochemical interface is crucial for ion transport, charge transfer, and surface reactions in aqueous rechargeable zinc batteries (ARZBs). However, Zn anodes routinely encounter persistent dendrite growth and parasitic reactions, driven by the inhomogeneous charge distribution and water-dominated environment within the EDL. Compounding this, classical EDL theory, rooted in mean-field approximations, further fails to resolve molecular-scale interfacial dynamics under battery-operating conditions, limiting mechanistic insights. Herein, we established a multiscale theoretical calculation framework from single molecular characteristics to interfacial ion distribution, revealing the EDL’s structure and interactions between different ions and molecules, which helps us understand the parasitic processes in depth. Simulations demonstrate that water dipole and sulfate ion adsorption at the inner Helmholtz plane drives severe hydrogen evolution and by-product formation. Guided by these insights, we engineered a “water-poor and anion-expelled” EDL using 4,1',6'-trichlorogalactosucrose (TGS) as an electrolyte additive. As a result, Zn||Zn symmetric cells with TGS exhibited stable cycling for over 4700 h under a current density of 1 mA cm^−2^, while NaV_3_O_8_·1.5H_2_O-based full cells kept 90.4% of the initial specific capacity after 800 cycles at 5 A g^−1^. This work highlights the power of multiscale theoretical frameworks to unravel EDL complexities and guide high-performance ARZB design through integrated theory-experiment approaches.

**Supplementary Information:**

The online version contains supplementary material available at 10.1007/s40820-025-01915-w.

## Highlights

- A multiscale theoretical framework deciphers the molecular-ionic dynamics of the electric double layer (EDL) in aqueous rechargeable zinc batteries, correlating interfacial water aggregation, anion-specific adsorption, and electric field inhomogeneity to parasitic reactions and dendrite growth, thereby establishing EDL-driven design principles for ultra-stable Zn anodes.
- Molecular adsorption engineering creates a localized “water-poor and anion-expelled” EDL configuration that suppresses hydrogen evolution and by-product formation while enabling dense Zn electrodeposition through flattened interfacial potential gradients and reduced Zn^2+^ electrostatic repulsion.

**Supplementary Information:**

The online version contains supplementary material available at 10.1007/s40820-025-01915-w.

## Graphical abstract

**Figure:**
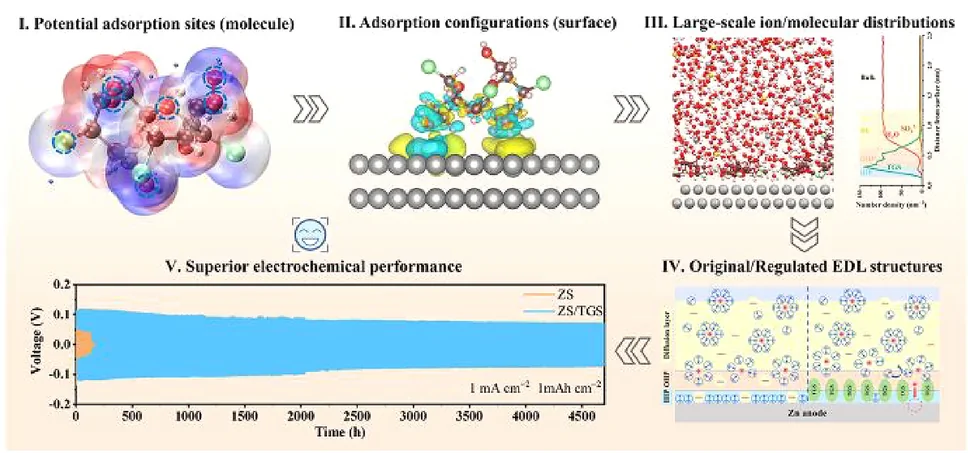

## Introduction

Electrochemical reaction processes, dynamically occurring at electrochemical interfaces, are the cornerstone of energy storage and conversion systems, dictating the performance and stability of devices such as batteries, supercapacitors, and fuel cells [1–3]. Due to the inner electric field between the anode and the cathode, or the difference in the equilibrium state between the electrode and the bulk electrolyte, a rearrangement of the solvated ions, solvent molecules, and adsorbed species takes place on the electrode surface, which leads to the formation of an electrochemical potential difference from the surface to the bulk electrolyte [4, 5]. Under this potential difference, an electric double layer (EDL) forms at the interface between the electrode and the liquid electrolyte, representing one of the most fundamental and critical concepts in electrochemistry [6]. Most importantly, the properties of the EDL directly govern the electrochemical reaction processes at the electrode-electrolyte interfaces, thereby influencing the electrochemical responses and performances of practical devices [7, 8].

The understanding of the EDL structure dates back to the early nineteenth century [9], beginning with Helmholtz’s pioneering work in 1879 (Fig. S1). Building on Helmholtz’s foundational contributions, researchers made significant advancements, culminating in the well-known Gouy-Chapman-Stern (GCS) model [10]. This model divides the EDL into two layers: (1) the Stern layer (or Helmholtz plane, HP), which interacts directly with the electrode surface, and (2) the Gouy–Chapman layer (or diffuse layer, DL), which extends into the bulk electrolyte. Despite its widespread use in studying electrode-electrolyte interfaces [11–14], the GCS model fails to account for the specific adsorption of water molecules and anions, leading to inaccuracies in describing the detailed distribution of ions and solvents at the interface. To address these limitations, further electrochemical studies were conducted by Grahame [15] and Bockris et al. [16], ultimately resulting in the detailed Bockris-Devanathan-Müller (BDM) model. The BDM model refines the HP by dividing it into an inner Helmholtz plane (IHP) and an outer Helmholtz plane (OHP). It reveals that water molecules near the electrode surface orient directionally due to their dipole interactions with the surface. As a result, the IHP becomes a complex mixture of specifically adsorbed ions and oriented water molecules, while the OHP is populated primarily by solvated ions. This insight into the structured arrangement of water and ions at the interface has profoundly deepened our understanding of interfacial phenomena in electrochemical systems.

Aqueous rechargeable zinc batteries (ARZBs), due to their low cost, high safety, and environmental compatibility, have emerged as one of the most attractive alternatives for large-scale energy storage [17–19]. However, the Zn metal anode is confronted with issues such as rampant dendrite growth and severe parasitic reactions, which occur at the anode–electrolyte interface (AEI) and result in low Coulombic efficiency (CE) during the Zn plating/stripping process [20–22]. As reported by previous research [23], the EDL structure plays a decisive role in governing ion transport, charge distribution, and Zn deposition at AEI. On the one hand, the specific adsorption of ions and water molecules within IHP induces inhomogeneous charge distribution on the Zn anode surface, which guides Zn^2+^ diffusion to the prior nucleation sites and promotes the uneven Zn deposition with dendritic morphology [24, 25]. On the other hand, the solvating water in the Zn^2+^ shell would migrate from the bulk electrolyte to the EDL and be pulled across the IHP, which has a higher reduction potential than free water due to the positive charge in the cation, making the hydrogen more reactive [26, 27]. Consequently, these water molecules would be decomposed to release H_2_ along with the reduction of Zn^2+^, which is known as the hydrogen evolution reaction (HER). Despite the critical role of EDL in stabilizing the Zn anode, investigating the EDL at AEI notoriously remains challenging due to the limitations of classical mean-field descriptions for the classical EDL models [4, 5, 28, 29]. Therefore, there is a pressing need to address key unsolved questions: What are the specific molecular and ion distributions within the EDL? Are anions specifically adsorbed, and if so, in which layer? How can the EDL structure be adjusted in scenarios where side reactions dominate? To address these complexities, multiscale simulations, particularly those focusing on interfacial dynamics and ion-specific interactions [30], offer a powerful approach to bridging the gap between classical theory and experimental observations.

**Fig. 1:**
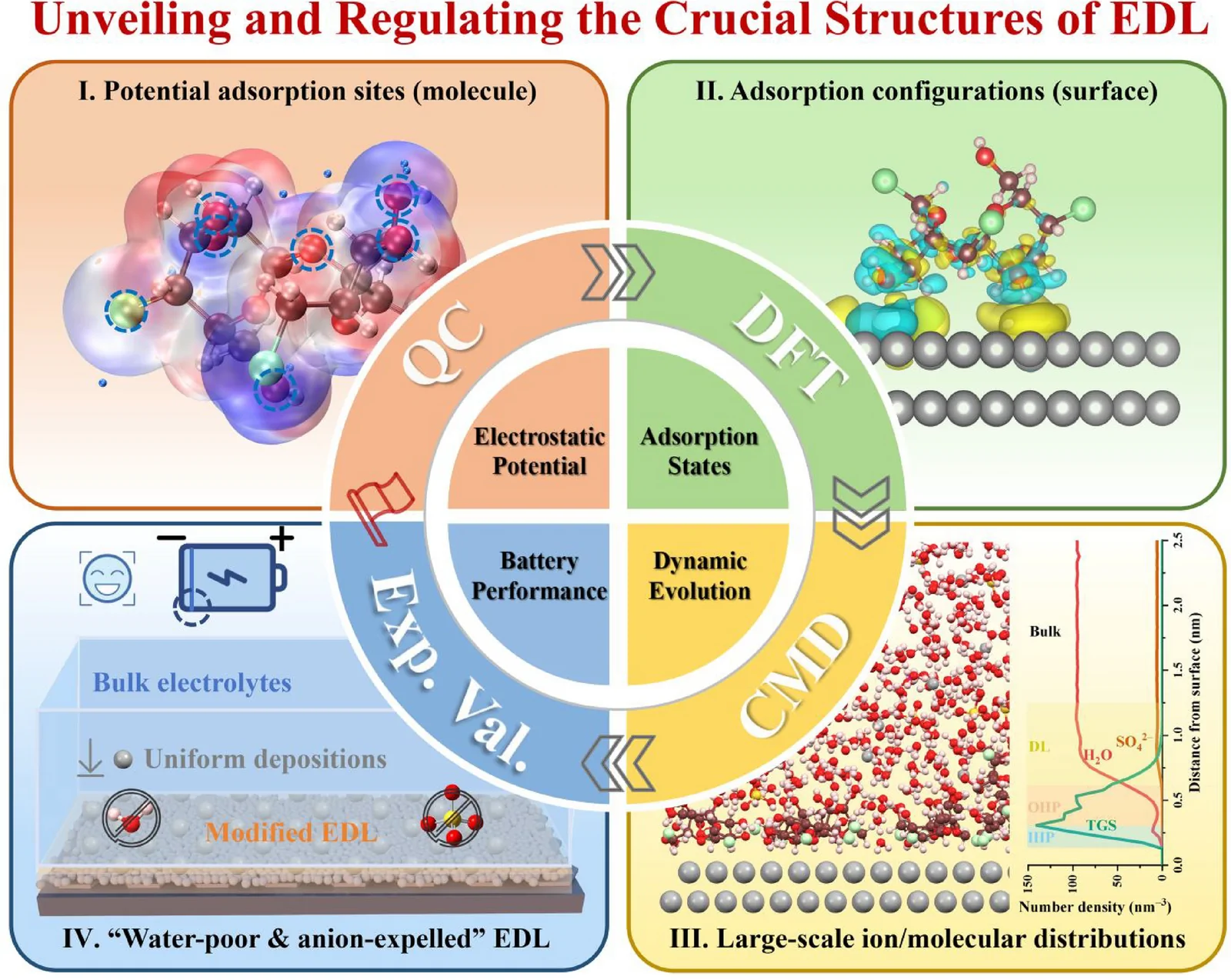
Schematic illustration of an advanced multiscale theoretical calculations framework and experimental validations to reveal regulated EDL structures. Exp. Val. is an abbreviation for experimental validations

In this study, we present an innovative approach to investigating EDL by employing a multiscale simulation platform that integrates quantum chemistry (QC), density functional theory (DFT), and classical molecular dynamics (CMD), further validated by characterization techniques and battery performance (Fig. 1). In detail, the potential adsorption sites for molecules on the Zn metal surface could be identified by QC calculations. Subsequently, DFT-based surface adsorption calculations are performed to characterize adsorption states, including specific adsorption sites, orientations, charge density, and corresponding adsorption energies, which provide insights into the preferential adsorption of certain molecules. Finally, CMD simulations are employed to reveal the dynamic processes of EDL formation and to model the molecular/ionic distributions near the AEI, offering a deeper understanding of the surface hydrogen bond (H-bond) network. Our integrated multiscale simulation platform provides a unique and comprehensive framework for interpreting EDL behavior, equipping researchers with valuable insights for efficient modification of EDL structure toward a robust Zn anode.

In virtue of this multiscale calculation framework, the EDL structure in typical zinc sulfate (ZnSO_4_) aqueous electrolytes is characterized as follows: the IHP consists of orderly adsorbed water dipoles and SO_4_^2−^ anions, which are tightly bound to the Zn metal surface. In contrast, the OHP is predominantly occupied by hydrated complexes, such as Zn^2+^(H_2_O)_6_ or Zn^2+^(H_2_O)_5_SO_4_^2−^, which are more loosely associated with the surface. Beyond the HP, the DL contains ions in equilibrium with the bulk electrolyte, contributing to the overall ionic concentration near the electrode interface. The molecular and ionic distributions revealed by these simulations align with the predictions of the classical BDM model. However, the presence of abundant free water molecules and SO_4_^2−^ anions within HP leads to undesirable parasitic reactions. These reactions accelerate the corrosion of the Zn anode and generate harmful hydroxide ions (OH^−^) within the IHP [31, 32], resulting in the formation of insulating and uneven by-products, such as Zn_4_SO_4_(OH)_6_·*x*H_2_O (ZSH) [33]. Besides, the deteriorative surface condition further exacerbates the tip effect, resulting in uncontrollable Zn dendrite growth, which degrades the long-term stability of the Zn anode [25].

To construct a “water-poor and anion-expelled” EDL structure to stabilize the Zn anode, we investigate the effect of 4,1',6'-trichlorogalactosucrose (TGS), a promising and cost-effective electrolyte additive, as a case study. The modulation of the EDL structure is intricately influenced by both substrate architecture and electrolyte composition. Previous strategies based on substrate architecture engineering, primarily by modifying surface charge density [12, 34], have been proven to be effective in regulating the EDL structure and reshaping the interface-localized distribution of ions, thereby promoting uniform Zn deposition and suppressing parasitic reactions [35–37]. In contrast, electrolyte design, especially through the incorporation of functional additives, provides a more versatile, solution-processable, self-adaptive, and dynamic approach for modulating the EDL at the electrode-electrolyte interface [31, 38–40]. Our additive-based EDL engineering strategy distinguishes itself by enabling targeted regulation of interfacial ion distributions without altering the electrode structure, thereby achieving enhanced Zn reversibility via a readily facile approach. Specifically, TGS is introduced to regulate the distribution and chemical environment of H_2_O and SO_4_^2−^ within the HP. The simulation results reveal that TGS spontaneously adsorbs into the IHP of the EDL, disrupting the aggregation of H_2_O molecules and creating a water-poor environment near the Zn surface. Simultaneously, the surface H-bond network is reconstructed due to the adsorption of TGS molecules. Additionally, SO_4_^2−^ anions are expelled from the IHP and OHP, effectively suppressing the formation of ZHS. By modifying the EDL with TGS, the overpotential increases, and the critical nucleation size decreases, promoting uniform Zn deposition and reducing dendrite formation. The efficacy of the modified EDL structure with TGS additive, given by this multiscale simulation platform, is demonstrated by the enhanced Zn anode performance. Encouragingly, Zn||Cu asymmetric cells using TGS electrolyte additive achieved stable cycling for over 1100 cycles at a current density of 1 mA cm^−2^, with an average CE of 99.49%. In parallel, Zn||Zn symmetric cells maintained a stable operation lifespan for over 4700 h at 1 mA cm^−2^, significantly outperforming cells with conventional ZnSO_4_ electrolytes. Moreover, full cells incorporating NaV_3_O_8_·1.5H_2_O cathodes also exhibited superior performance with TGS, achieving a high capacity retention of 90.4% after 800 cycles at 5 A g^−1^.

## Computational Methods

### Quantum Chemistry Calculations

Quantum chemistry calculations were conducted using the Gaussian 16 software package. Structural optimizations and frequency calculations for the electrostatic potential (ESP) were performed at the M06-2X/def2-TZVP level of theory [41, 42]. To accurately simulate the aqueous environment, the universal solvation model based on density (SMD) was employed [43], ensuring a precise representation of solvation effects. The independent gradient model based on Hirshfeld (IGMH) [44] was employed to investigate the interactions between TGS and H_2_O. This model provides an accurate framework for understanding interactions in chemical systems [44]. The molecular surface electrostatic potential was plotted using Multiwfn software [45, 46].

For solvation free energy calculations, geometry optimizations of the studied systems were executed using the M06-2X functional with the TZVP basis set. Subsequently, single-point energy calculations were performed at the M06-2X/def2-TZVPP level of theory. The solvation energy was derived from the single-point energy difference calculated using the M05-2X/6-31G* level of theory, both in the gas phase and within the SMD solvation model (using water as the solvent). Based on prior molecular dynamics and experimental studies, the coordination number of Zn^2+^ was constrained to six [47].

The free energy (*G*) of the solute in a solvent environment (at 298.15 K and 1 M concentration) was calculated using the following formula [48]:

$${\mathrm{G}}_{{\mathrm{s}}{\mathrm{olvent}}}\, {=}\, {\mathrm{G}}_{\mathrm{gas}}{+}{\mathrm{G}}_{\mathrm{solution}}{+1.89\, \mathrm{kcal}} \, {\mathrm{mol}}^{-{1}}$$

where ${\mathrm{G}}_{\mathrm{gas}}$ represents the gas-phase free energy of the solute at 1 atm, ${\mathrm{G}}_{\mathrm{solution}}$ denotes the free energy of solvation calculated using the implicit solvent model, and 1.89 kcal mol^−1^ accounts for the free energy change from 1 atm to 1 M standard state conditions. The relative free energies (Δ*G*) for the Zn^2+^ solvates, referenced to Zn^2+^(H_2_O)_6_, were defined as follows [48]:

$$\Delta G = G_{{[{\mathrm{Zn}}^{2 + } ({\mathrm{H}}_{2} {\mathrm{O}})_{x} ({\mathrm{n}})_{(6 - x)} ]}} - G_{{\left[ {{\mathrm{Zn}}^{2 + } \left( {{\mathrm{H}}_{2} {\mathrm{O}}} \right)_{6} } \right]}} + (6 - x)G_{{{\mathrm{H}}_{2} {\mathrm{O}}}} - (6 - x)G_{{\mathrm{n}}}$$

where *x* represents the coordination number of Zn^2+^, with *x* = 5, 6. This approach facilitates a comprehensive analysis of the solvation structures and energetics of Zn^2+^ across various solvation environments.

The binding energy (${\mathrm{E}}_{\mathrm{bind}}$) is calculated using the following formula:

$${\mathrm{E}}_{\mathrm{bind}} {=}\, {\mathrm{E}}_{\mathrm{AB}}-{\mathrm{E}}_{\mathrm{A}}-{\mathrm{E}}_{\mathrm{B}}-{\mathrm{E}}_{\mathrm{BSSE}}$$

where ${\mathrm{E}}_{\mathrm{AB}}$, ${\mathrm{E}}_{\mathrm{A}}$, and ${\mathrm{E}}_{\mathrm{B}}$ represent the total energies of the AB complex, the isolated species A, and the isolated species B, respectively. ${\mathrm{E}}_{\mathrm{BSSE}}$ denotes the basis set superposition error (BSSE) correction energy, which is applied using the counterpoise method [49].

### Density Functional Theory Calculations

The Vienna Ab-initio Simulation Package (VASP) software [50, 51] was utilized to conduct adsorption and HER barrier calculations. The Perdew-Burke-Ernzerhof (PBE) functional within the generalized gradient approximation (GGA) was employed [52]. The projector augmented-wave (PAW) method was applied to accurately describe the interactions between core and valence electrons [53]. An energy cutoff of 500 eV was set, and a Γ-centered k-point mesh grid of 2 × 2 × 1 was used for geometry optimization. Grimme’s DFT-D3 method was also implemented to account for van der Waals (vdW) interactions [54].

The computational model consisted of a Zn (002) surface slab composed of four Zn atomic layers, with the bottom two layers fixed. A vacuum layer of 15 Å was included to avoid interactions between periodic images. Atomic positions were relaxed until the energy converged to less than 10^−5^ eV, and the maximum force on any atom was reduced to less than 0.02 eV Å^−1^.

The adsorption energy (${\mathrm{E}}_{\mathrm{ads.}}$) is calculated using the following equation:

$${\mathrm{E}}_{\mathrm{ads.}}={\mathrm{E}}_{\mathrm{slab/adsorbate}}-{\mathrm{E}}_{\mathrm{slab}}-{\mathrm{E}}_{\mathrm{adsorbate}}$$

where ${\mathrm{E}}_{\mathrm{slab/adsorbate}}$ is the total energy of the slab with the adsorbate, ${\mathrm{E}}_{\mathrm{slab}}$ is the energy of the clean slab, and ${\mathrm{E}}_{\mathrm{adsorbate}}$ is the energy of the isolated adsorbate molecule.

The HER activity was investigated under acidic conditions, incorporating van der Waals interactions to provide an accurate description of the system. The Gibbs free energy change (Δ*G*_H_), a critical parameter for characterizing HER activity, is determined using the following equation [55]:

$$\Delta G_{{\mathrm{H}}} = \Delta E_{{\mathrm{H}}} + \Delta E_{{{\mathrm{ZPE}}}} - T\Delta S_{{\mathrm{H}}}$$

where $\Delta E_{{\mathrm{H}}}$ is the change in electronic energy, $\Delta E_{{{\mathrm{ZPE}}}}$ is the change in zero-point energy, T is the temperature, and $\Delta S_{{\mathrm{H}}}$ is the change in entropy. This comprehensive approach enables an accurate assessment of the adsorption properties and HER activity on the Zn (002) surface, providing valuable insights into the electrochemical behavior of the system.

### Bulk Classical Molecular Dynamics

Classical molecular dynamics simulations were conducted using GROMACS 2022.2 [56]. The OPC3 water model was utilized to simulate water molecules [57], while the Merz force field was employed for Zn ions [58], as these models provide optimal accuracy for our system. The general Amber force field (GAFF) parameters [59] for sulfate ions and TGS molecules were generated using the Sobtop program, with atomic charges derived from restrained electrostatic potential (RESP2) calculations [60] performed by Multiwfn [46]. The simulation system consisted of 4440 water molecules, 160 Zn ions, 160 sulfate anions, and 40 TGS molecules. Atomic charges of all ions were multiplied by a scale factor of 0.7 to correct the polarization effect of ions.

The simulation protocol commenced with energy minimization using the steepest descent method to eliminate any unfavorable interactions. Following this, the system was gradually heated from 10 to 298.15 K over a period of 100 ps, followed by an additional 100 ps at the target temperature to ensure stabilization. Pre-equilibration was performed under isothermal-isobaric (NPT) conditions at 1 bar for 10 ns. The production run involved a 20 ns simulation under the canonical ensemble (NVT).

A time step of 1 fs was employed for all simulations. In the NPT simulations, temperature control was achieved using a V-rescale thermostat [61] with a time constant of 1 ps, while pressure was maintained with the Berendsen barostat [62] with a time constant of 3 ps. For the NVT simulations, a Nosé-Hoover thermostat [63, 64] with a time constant of 1 ps was used. Electrostatic interactions were calculated using the particle-mesh Ewald (PME) method [65, 66], with a cutoff distance of 1.2 nm. System visualization and analysis of ion association states were carried out using VMD software [67].

### Interfacial Classical Molecular Dynamics

An interfacial model, illustrated in Fig. S23, was developed to investigate the EDL structures. This model comprises two Zn metal electrodes, an electrolyte containing an equivalent number of electrolyte species as those employed in bulk simulations, and a vacuum layer to replicate the interfacial environment.

The dimensions of the periodic simulation cells in the *x*- and *y*-directions are 55.12 Å × 55.69 Å for ZS electrolyte and 55.28 Å × 55.85 Å for ZS/TGS electrolyte, as recorded in Table S3. The electrolyte thickness separating the two Zn metal anodes is approximately 50.0 Å; specifically, for the ZS electrolyte, the thickness is 49.96 Å, while for the ZS/TGS electrolyte, it measures 53.64 Å. These values were obtained through equilibration simulations, with the detailed methodology illustrated in Fig. S22. The selected thickness is sufficiently substantial to ensure the manifestation of bulk electrolyte behavior in the central region of the simulation cell [68].

In these simulations, the Zn anodes were fixed in position, and the Lennard-Jones parameters for the Zn atoms were defined as *σ* = 2.44 Å and *ε* = 3.022 kJ mol^−1^, which have been shown to provide accurate representations of the interfacial interactions [32]. In the subsequent calculations, the left Zn anode is assigned a positive charge, while the right anode is negatively charged, in accordance with considerations of the potential of zero charge (PZC).

For the force field parameters governing the molecules and ions (Zn^2+^, SO_4_^2−^, H_2_O, and TGS), the same parameters as those utilized in the bulk electrolyte simulations were employed to ensure consistency across the simulations. Following the pre-equilibration simulations of the bulk electrolyte, Zn (002)-electrolyte-Zn (002) structures were constructed. Energy minimizations were performed using the steepest descent method to optimize the system configuration. Subsequently, the system was subjected to a heating protocol, gradually increasing the temperature from 10 to 600 K over a period of approximately 100 ps. This was followed by an additional equilibration period of 100 ps at the target temperature. The relatively high temperature of 600 K was selected to facilitate the formation of uniform and saturated EDL structures within the constraints of the limited simulation time [69].

For the interface simulations, pre-equilibration was conducted under NPT conditions at a pressure of 1 bar for a duration of 10 ns. The production run consisted of a 20 ns simulation performed under the NVT, employing the same thermostat and barostat configurations as those used in the bulk electrolyte simulations. 2D H_2_O number density distribution on the Zn (002) surface was analyzed by the Density Calculator program [70].

## Results and Discussion

### Solvation Structure and Adsorption Characteristics Investigations

**Fig. 2:**
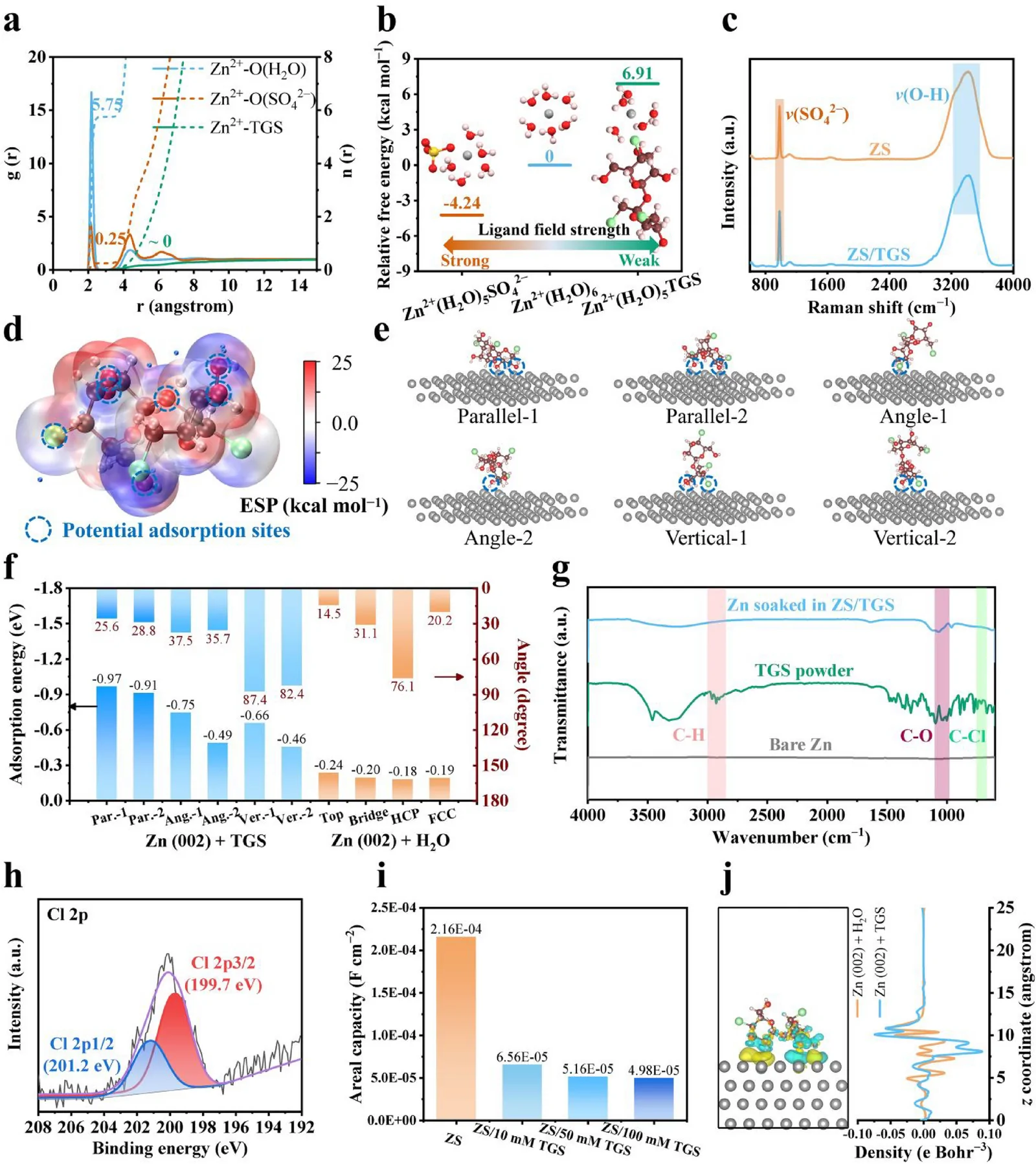
Solvation structure and adsorption characteristics investigations. **a** RDFs and CNs of bulk electrolytes after the introduction of TGS. **b** Relative free energy comparison of Zn^2+^(H_2_O)_5_SO_4_^2−^, Zn^2+^(H_2_O)_6_, and Zn^2+^(H_2_O)_5_TGS complexes. **c** Raman spectra show characteristic vibrational modes in different electrolytes. **d** ESP mapping of TGS, with blue dotted rings marking potential adsorption (nucleophilic) sites. **e** Various adsorption configurations of TGS on the Zn (002) surface. **f** Adsorption energy and adsorbate–surface angles for different configurations of TGS and H_2_O on the Zn (002) surface. **g** FTIR spectra of Zn foil soaked in ZS/TGS electrolyte for 3 days, compared with TGS powder, indicate the adsorption of TGS on the Zn surface. **h** XPS Cl 2p spectra of Zn foils soaked in the ZS/TGS electrolyte for 3 days. **i** Average differential capacitance comparison of Zn anodes in different electrolytes. **j** CDD distribution plots of Zn (002) + TGS and the 2D planar-average CDD plots (iso-value = 5 × 10^−4^ e Bohr^−3^). The yellow and cyan iso-surfaces represent regions of increased and decreased charge density, respectively

To modulate the EDL structure, TGS was selected as an electrolyte additive owing to its abundant polar functional groups, namely multiple hydroxyl (–OH) and chlorine (–Cl) moieties, and its relatively large molecular size (Fig. S2). These structural features are expected to promote the spontaneous adsorption of TGS onto the Zn metal surface, where it can exert a pronounced steric hindrance effect [71–73]. This interaction is anticipated to significantly influence the interfacial configuration and disrupt the local H-bond network. Furthermore, TGS serves as a model additive for the construction of a transferable multiscale theoretical platform, enabling the systematic regulation of EDL structures in electrochemical systems. Specifically, 2 M ZnSO_4_ (ZS) electrolytes with different concentrations of TGS (10/50/100 mM) were used to assemble Zn||Zn symmetric cells. Under the galvanostatic condition of 1 mA cm^−2^ and 1 mAh cm^−2^, the sample with 50 mM TGS exhibits an exceptionally stable cycle life exceeding 4700 h (Fig. S3). In comparison, the cell containing 10 mM TGS demonstrates an extended lifespan of approximately 4060 h, while it encounters a soft short circuit at around 3860 h [74]. Moreover, when the concentration of TGS is further increased to 100 mM, the cycling lifespan is markedly shortened to approximately 500 h. This degradation in performance is primarily attributed to a pronounced decrease in bulk ionic conductivity and a substantial increase in interfacial impedance, as evidenced by Figs. S4 and S5. Based on these observations, an optimized TGS concentration of 50 mM in ZS (ZS/TGS) was selected for further investigation. We first explored the bulk electrolyte solvation structures using CMD simulations, as outlined in Figs. S6 and S7. Fig. 2a presents the radial distribution functions (RDFs) and coordination numbers (CNs) for Zn^2+^ in the ZS/TGS electrolyte. Notably, negligible changes in RDFs and CNs are observed compared to that of the ZS electrolyte (Fig. S8), indicating that TGS does not alter the primary solvation shell of Zn^2+^. Specifically, the predominant solvation structures remain ~ 75% Zn^2+^(H_2_O)_6_ and ~ 25% Zn^2+^(H_2_O)_5_SO_4_^2−^ complexes in Fig. S9. The absence of TGS in the solvation shell can be rationalized by examining the relative free energy of Zn^2+^ solvation complexes based on ligand field effects (Fig. 2b) [48]. The formation of a Zn^2+^(H_2_O)_5_TGS complex is associated with a higher relative free energy, calculated to be 6.91 kcal mol^−1^ compared with the much lower free energy of − 4.24 kcal mol^−1^ of Zn^2+^(H_2_O)_5_SO_4_^2−^ complex. This significant difference in relative free energy compared with Zn^2+^(H_2_O)_6_ indicates that the sulfate ion (SO_4_^2−^) is more easily involved in the first solvation shell of Zn^2+^ ion [47, 75]. Further corroboration of the minimal impact of TGS on the solvation structure is provided by the Raman spectra and nuclear magnetic resonance (NMR) results, as shown in Figs. 2c and S10. There is nearly no chemical shift for the stretching vibrations of O–H and SO_4_^2−^ and the characteristic peaks of ^2^H, indicating that TGS would not change the coordination environment of Zn^2+^ ions, which is consistent with the results in the Fourier transform infrared (FTIR) spectra (Fig. S11) and the theoretical analysis above. In spite of the conclusion drawn above that TGS does not significantly affect the solvation structures of Zn^2+^, noticeable molecular aggregation of TGS was detected (Fig. S7b), which may impact the ion diffusion process [76]. As expected, the diffusion coefficients of Zn^2+^, SO_4_^2−^, and H_2_O in the bulk electrolyte decreased upon the introduction of TGS, as revealed by the mean squared displacement (MSD) analysis in Fig. S12, demonstrating the significant steric hindrance effect of aggregated TGS molecules. Notably, these findings are consistent with the previously observed decline in bulk ionic conductivity after introducing the TGS. Hereto, it is reasonable to conjecture that the TGS molecule may work on the surface because of the non-negligible steric hindrance effect.

To elucidate the molecular characteristics of TGS, we calculated its ESP using QC methods. Figs. 2d and S13 illustrate the ESP mappings of TGS and H_2_O molecules, where the ESP minima regions (blue points) located near the atoms with negative ESP values (blue areas), such as oxygen and chlorine atoms, could be identified as nucleophilic (potential adsorption) sites [77–79]. The ESP extrema of specific potential adsorption sites are recorded in Table S1. Subsequently, DFT calculations were performed to investigate various adsorption configurations on the Zn (002) surface, which was selected due to its lowest surface energy, indicating it as the most thermodynamically stable facet [77]. The adsorption states were categorized as ‘parallel’, ‘angle’, and ‘vertical’, corresponding to the molecular orientations parallel to, at an angle with respect to, or perpendicular to the surface, respectively (Fig. 2e, f). The results reveal that the TGS molecule reaches the most stable adsorption state when it is parallel to the Zn (002) surface, as indicated by the more negative adsorption energies (~ − 0.97 eV) and smaller adsorption angles (~ 25°) compared with other configurations (Table S2). Moreover, the adsorption energies of all TGS configurations were found to be higher than those of H_2_O at the TOP, Bridge, HCP, and FCC sites (Figs. 2f and S14), highlighting the stronger binding affinity of TGS molecules to the Zn metal surface. Noticeably, the adsorption sites of TGS and H_2_O molecules are both consistent with the predictions of QC calculations before (mark potential adsorption sites with blue dot rings).

With the revelation of the specific adsorption behavior and conditions for the TGS and H_2_O molecules on the Zn metal surface by QC and DFT calculations, we further performed spectroscopy analyses to verify the interaction between the TGS molecule and the Zn anode. Fig. 2g presents the FTIR spectra of the Zn anode soaked in the ZS/TGS electrolyte for 3 days. The adsorbed peaks at ≈2900, ≈1000, and 700−750 cm^−1^ correspond to the stretching vibrations of C–H, C–O, and C–Cl from the TGS molecule, respectively [80], explicitly indicating the spontaneous adsorption of TGS on the Zn anode in aqueous solutions. The X-ray photoelectron spectroscopy (XPS) results collected in Figs. 2h, S15, and S16 provide additional confirmation of TGS adsorption. For the Zn anode soaked in ZS/TGS electrolytes for 3 days (Fig. 2h), distinct Cl peaks are observed at the binding energies of 199.7 eV (Cl 2*p*_3/2_) and 201.2 eV (Cl 2*p*_1/2_) [81], elaborating the presence of chlorine atoms bonded to sp^2^ carbons. In addition, the introduction of TGS molecules reduces the contact angle of the electrolyte on the Zn foil from 78.3° to 67.4° (Fig. S17), signifying a marked improvement in surface wettability and the strong adsorption capability of TGS molecules on the Zn surface [82, 83]. Of note, the surface charge state of the Zn anode is identified by average differential areal capacity ($\overline{{C}}$), as shown in Figs. 2i, S18 and S19. The $\overline{{C}}$ is closely relevant to the EDL structure [84, 85], which can be derived from the following equation:

$$\overline{i} = \overline{C} \cdot v$$

where $\overline{i}$ is the average current density and ${\mathrm{v}}$ is the corresponding scan rate of the cyclic voltammetry (CV) curve. The introduction of TGS at varying concentrations leads to a notable decrease in $\overline{{C}}$ resulting in a water-poor EDL [85, 86]. Specifically, the capacitance drops significantly from 2.16 × 10^−4^ F cm^−2^ for the ZS electrolyte to 5.16 × 10^−5^ F cm^−2^ upon the addition of 50 mM TGS. However, further increase in the TGS concentration from 50 to 100 mM results in only a marginal change in capacitance (from 5.16 × 10^−5^ to 4.98 × 10^−5^ F cm^−2^), indicating that the adsorption of Zn surface becomes saturated with 50 mM TGS additive. Moreover, the decrease in zeta potential indicates a less negatively charged Zn surface with the presence of TGS (Fig. S20), likely driven by the adsorption of TGS molecules and their influence on the surface charge distribution or potential. On this basis, the charge density difference (CDD) distribution plots provide deeper insights into the adsorption characteristics of TGS and H_2_O on the Zn (002) surface, as showcased in Figs. 2i and S21. Much more dramatic charge exchanges between TGS and Zn (002) surface than those of H_2_O are witnessed, verifying the stronger chemisorption and surface charge transfer.

While QC and DFT calculations, supported by experimental results, have successfully elucidated the specific adsorption behavior of TGS molecules and proved the reorganization of EDL, inherent limitations are inescapable with regard to these approaches [87]. Specifically, experimental techniques provide only a macroscopic perspective of the AEI, whereas QC and DFT calculations are confined to the analysis of individual TGS and H_2_O molecules, overlooking the effects of molecular aggregation and orientation distribution [76]. Consequently, the EDL portrayed by these methods is incomplete. To overcome this limitation, CMD simulations were harnessed by the constant charge method (CCM) [88]. This approach endowed us with a systematic perspective on the interfacial behavior of TGS and H_2_O molecules on the Zn metal surface under realistic electrolyte conditions. By leveraging this computational framework, we aim to provide a comprehensive clarification of the structural and functional intricacies of the EDL and unravel the role of TGS additives in modulating interfacial properties.

### Dissection of the Electric Double Layers

**Fig. 3:**
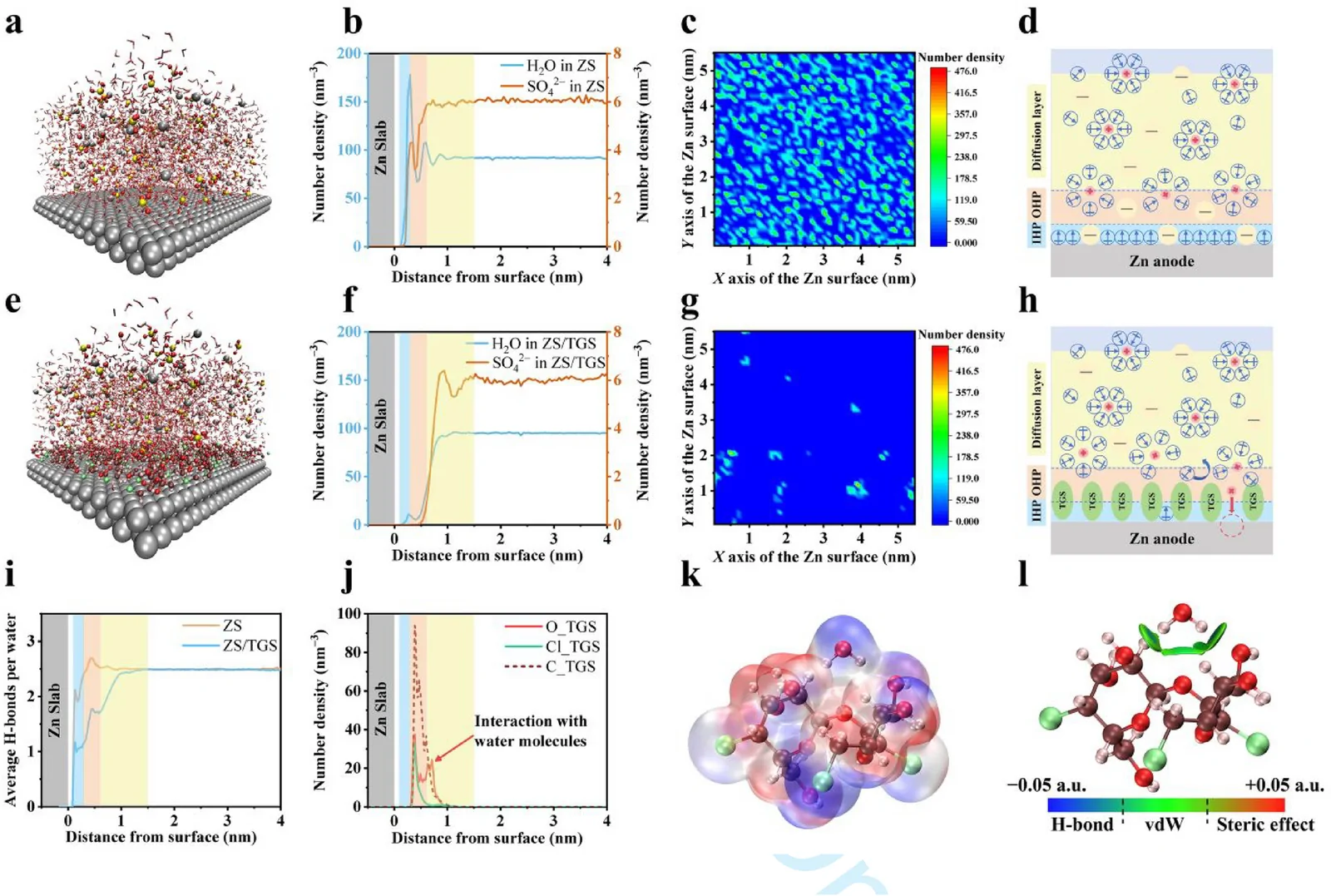
Interfacial behavior of electrolytes on the Zn (002) surface. CMD simulation snapshots of **a** ZS and **e** ZS/TGS electrolytes on the Zn (002) surface, illustrating distinct interfacial structures. Number density profiles of H_2_O and SO_4_^2−^ ions as a function of distance from the Zn metal surface in **b** ZS and **f** ZS/TGS electrolytes, with interfacial zones labeled as IHP (blue zone), OHP (orange zone), and DL (yellow zone). 2D number density distributions of H_2_O molecules on the Zn metal surface for **c** ZS and **g** ZS/TGS systems. Schematic diagrams of the EDL structures in **d** ZS and **h** ZS/TGS electrolytes. **i** Average H-bond number per water molecule as a function of distance from the Zn (002) surface. **j** Number density distributions of specific elements in TGS (O, Cl, and C) near the Zn (002) surface. **k** ESP mapping of the TGS + H_2_O system, consistent with the color bar of Fig. 2d. **l** IGMH analysis of TGS + H_2_O interactions, indicating vdW and weak H-bond interactions

Firstly, Fig. 3a–d illustrates the EDL structures of the Zn (002) surface in the ZS electrolyte, analyzed through CMD simulations (detailed simulation steps and structures are shown in Figs. S22 and S23, Table S3)*.* Fig. 3a shows a snapshot from CMD simulations, where the interfacial region exhibits distinct layering of H_2_O molecules and SO_4_^2−^ ions near the Zn surface. This interfacial structure is quantitatively characterized in Fig. 3b, which presents the number density profiles of H_2_O molecules and SO_4_^2−^ ions as a function of the distance from the Zn surface. Accordingly, there are three interfacial zones based on the classical BDM model: (I) H_2_O molecules and partial SO_4_^2−^ ions directly adsorbed on the Zn surface (IHP, blue zone); (II) dominated by loosely associated water and SO_4_^2−^ ions (OHP, orange zone); and (III) the diffuse layer (DL, yellow zone), where the ion concentrations gradually recover back to that of the bulk electrolyte. In the IHP, the tight and ordered adsorption of water molecules onto the Zn surface results in a pronounced first peak in the H_2_O number density, reaching its maximum value. This indicates strong electrostatic interactions between the H_2_O dipoles and Zn metal surface, leading to a well-organized interfacial water layer (Fig. S24). Moving into the OHP, the solvated Zn^2+^(H_2_O)_6_ ions disrupt the orderliness of water molecules, resulting in a more loosely bound configuration, as demonstrated by a rapid decline in the H_2_O number density. Concurrently, the larger OHP region allows more SO_4_^2−^ ions to penetrate and adsorb onto the Zn surface. As a result, the number density of SO_4_^2−^ ions exhibits a sharp increase from IHP to OHP and reaches a first peak in the number density profiles. At the boundary between the OHP and the DL, electrostatic interactions between H_2_O molecules and SO_4_^2−^ ions lead to a secondary peak in the number density profiles for both species [89, 90], corresponding to a region where water molecules and SO_4_^2−^ ions achieve a local maximum in their spatial distributions due to mutual electrostatic stabilization. In the DL, the concentration of H_2_O molecules gradually decreases as it transitions into the bulk electrolyte, where the structure becomes ordered. Similarly, the SO_4_^2−^ ion concentration experiences slight fluctuations, ultimately reaching the homogeneous bulk electrolyte value. Further insights into the spatial arrangement of interfacial water are provided by the 2D number density distribution of H_2_O molecules. As shown in Fig. 3c, a mass of water molecules is uniformly distributed across the Zn metal surface in the ZS electrolyte, indicating the isotropic water alignment on the Zn metal surface (Fig. S24). A conceptual summary of these findings is presented in Fig. 3d. In traditional ZS electrolytes, a significant accumulation of ordered H_2_O molecules and SO_4_^2−^ anions occurs in the IHP and forms a water-rich and anion-adsorbed EDL structure, dramatically promoting undesirable side reactions, including HER and the formation of by-products such as ZSH. As SO_4_^2−^ anions adsorb, they bring the negative charge closer to the electrode surface. In classical GCS models, the potential at the IHP is usually assumed to decrease relatively smoothly from the electrode potential to that of the bulk solution. However, the adsorbed anions create localized regions of high charge density. This can cause an immediate drop (or more pronounced “step”) in potential near the IHP because the electrode now faces a layer of fixed negative charges based on the BDM model (as depicted in Fig. S1). Therefore, the presence of specifically adsorbed anions can produce a more abrupt change in potential, often a steeper initial decrease followed by a more gradual decay through the diffuse layer, which could lead to non-uniform Zn^2+^ ion depositions [75, 91].

By contrast, Fig. 3e–h highlights the impact of TGS molecule adsorption on the Zn metal surface, leading to a reconstructed EDL and the creation of a water-poor interfacial environment. Figs. 3e and S25 demonstrate the adsorption and aggregation of TGS molecules in IHP and OHP. This structural transformation is quantitatively depicted in Fig. 3f. Due to the robust adsorption and aggregation of TGS on the Zn surface, the number density of H_2_O molecules in the IHP is significantly reduced. Similarly, the repulsion of SO_4_^2−^ ions away from the interfacial region leads to their lower density in the HP. The 2D number density distribution of water molecules, as shown in Fig. 3g, further highlights this TGS molecular aggregation effect. Compared to the relatively uniform distribution observed in the ZS system (Fig. 3c), the TGS-modified interface exhibits sparse and localized H_2_O adsorption, confirming the broken water layer structure and surface H-bond network [92]. To further evaluate molecular coverage, the surface coverage (*θ*) of TGS is defined by:

$$\theta = 1 - \frac{{n_{{H_{2} O}} ^{\prime } }}{{n_{{H_{2} O}} }}$$

where ${\mathrm{n}}_{H_{2}{\mathrm{O}}}$ and ${\mathrm{n}}^{\prime }_{H_{2}{\mathrm{O}}}$ represent the relative molecule number of H_2_O on the surface before and after TGS adsorption. The *θ* of TGS molecules reached 94.44%, indicating highly uniform adsorptions on the surface in a parallel configuration, which is consistent with the adsorption states predicted by previous DFT calculations. The schematic diagram in Fig. 3h summarizes the reconstructed EDL structure in the TGS-modified electrolyte. According to the Derjaguin-Landau-Verwey-Overbeek (DLVO) theory [93–95], the interactions between Zn^2+^ ion deposits in aqueous electrolytes are determined by the interplay between vdW attractive forces and the electrostatic repulsion arising from the EDL (Fig. S26). In ZS electrolytes, the strong EDL repulsion, driven by SO_4_^2−^ counterions near the HP, leads to scattered and loose platelet-like Zn deposition [96]. The introduction of TGS molecules effectively repels SO_4_^2−^ ions from the HP, reducing the surface charge density and redistributing the potential drop within the EDL (Fig. S27), which is consistent with the decrease in zeta potential. In other words, the energy barrier of the Zn^2+^ ion deposit could be reduced by lowering the net charges of particles. Furthermore, TGS flattens the potential profile and weakens the electrostatic repulsion between Zn^2+^ ion deposits, allowing the vdW force to dominate, which promotes dense and coherent deposition processes. These structural and electrostatic modifications improve the morphology of Zn^2+^ ion deposits, as discussed in the later section.

As is well known, the activity of H_2_O molecules primarily depends on the intensity of the H-bond network formed between the H and O atoms of adjacent H_2_O and directly affects the side reactions of H_2_O decomposition [97]. Fig. 3i quantifies the average number of H-bonds per water molecule on the Zn surface for both ZS and ZS/TGS electrolytes. In the presence of TGS, the number of H-bonds per water decreases significantly, dropping from 2.1 to 1.1 in the IHP and from 2.6 to 1.7 in the OHP, which reflects the disruption of the H-bond network at the EDL caused by TGS adsorption. This result is also observed from surface structure snapshots (Fig. S28), breaking the continuous H-bond network in the ZS/TGS electrolyte. Fig. 3j highlights that those electron-rich atoms (such as O and Cl) from the TGS molecule would effectively participate in the IHP and OHP region and modify the EDL structure, due to their strong affinity to the Zn surface (Fig. 2e). At the same time, partial O atoms in TGS could interact with water molecules to avoid them in the HP. To further investigate the interactions between TGS and H_2_O molecules, ESP mapping of the TGS + H_2_O system was performed, as shown in Fig. 3k. The ESP mapping reveals intensive interactions between TGS and H_2_O molecules. Specifically, the nucleophilic site of TGS (three oxygen atoms) can bind well to the electrophilic site of H_2_O molecules (two hydrogen atoms) and further change the electrostatic potential at the binding site. This is further supported by the binding energy calculations (Fig. S29), as shown that the TGS-H_2_O interaction (− 9.90 kcal mol^−1^) is much stronger than that of H_2_O-H_2_O (− 5.14 kcal mol^−1^). Moreover, the IGMH analysis is conducted to provide deeper insight into TGS-H_2_O interactions (Figs. 2l and S30) [44]. The IGMH visualization identifies vdW interactions and weak H-bonds as the key stabilizing factors between TGS and H_2_O molecules [98]. As a result, the complex interactions between TGS and H_2_O molecules significantly break the H-bond network of H_2_O at the interface and reduce the activity of free water, thus reconstructing a water-poor EDL with inhibited side reactions for the Zn anode.

To account for the potential-dependent behavior of the Zn metal anode, the Zn (002) surface was polarized from the PZC [99]. Charge distribution on the Zn (002) surface under varying polarization conditions was calculated using joint density functional theory (JDFT) [100, 101], as implemented in the JDFTx software [102]. Fig. S31 illustrates the variation in charge density on the Zn (002) surface as a function of potential relative to the PZC, serving as a foundation for understanding the interfacial behavior under different polarization conditions [31]. When the Zn surface potential is polarized to + 0.5 and − 0.5 V from the PZC, distinct adsorption phenomena are observed, as illustrated in Figs. S32-S35. Specifically, TGS molecules become continuously adsorbed and enriched on the Zn (002) surface under both positive and negative polarization conditions (Fig. S28). Figs. S33 and S34 also confirm the reduction in H_2_O and SO_4_^2−^ concentrations at the EDL, highlighting the role of TGS in mitigating the undesired side reactions. Further analysis of the H-bond network on the Zn surface under different polarization conditions is also performed (Fig. S35). In ZS electrolytes, the average number of surface H-bonds displays distinct characteristics based on the applied potential. At + 0.5 V *vs.* PZC, the first peak of the H-bond count is lower than the bulk H-bond value, consistent with observations at the PZC situation, which reflects the relatively loose H_2_O-H_2_O interactions near the positively charged surface. Conversely, at − 0.5 V *vs.* PZC, the first peak of the H-bond count is higher than the bulk H-bond value. This increase results from the accumulation of negative charge, which promotes the ordered and compact arrangement of water molecules and enhances H-bond density. To be noted, the adsorption of TGS molecules on both sides can effectively reduce the number of H-bonds on the surface and change the local EDL environment. At − 0.5 V *vs.* PZC, the first peak of the H-bond count is significantly reduced after introducing TGS molecules, which is lower than the bulk H-bond numbers.

In summary, the CMD simulations and subsequent multidimensional analyses reveal that the introduction of TGS into the ZS electrolyte leads to significant reconstruction of the EDL at the Zn metal surface. TGS molecules preferentially adsorb onto the Zn surface, displacing H_2_O molecules and SO_4_^2−^ anions. This adsorption reduces surface H-bond and redistributes the potential drop, creating a water-poor and anion-expelled EDL environment that suppresses parasitic reactions and enhances the stability and performance of the Zn anode. Our coupled multiscale simulation platform provides a clear depiction of the EDL structure, ion/molecular distribution, and interfacial behaviors, seamlessly integrating QC, DFT, and CMD. This approach offers valuable insights into strategies for EDL regulation theoretically. Subsequent experimental results will further validate the accuracy and predictive phenomena of these theoretical calculations.

### Evaluation of Anti-Corrosion and Deposition Kinetics of the Zn Anode

**Fig. 4:**
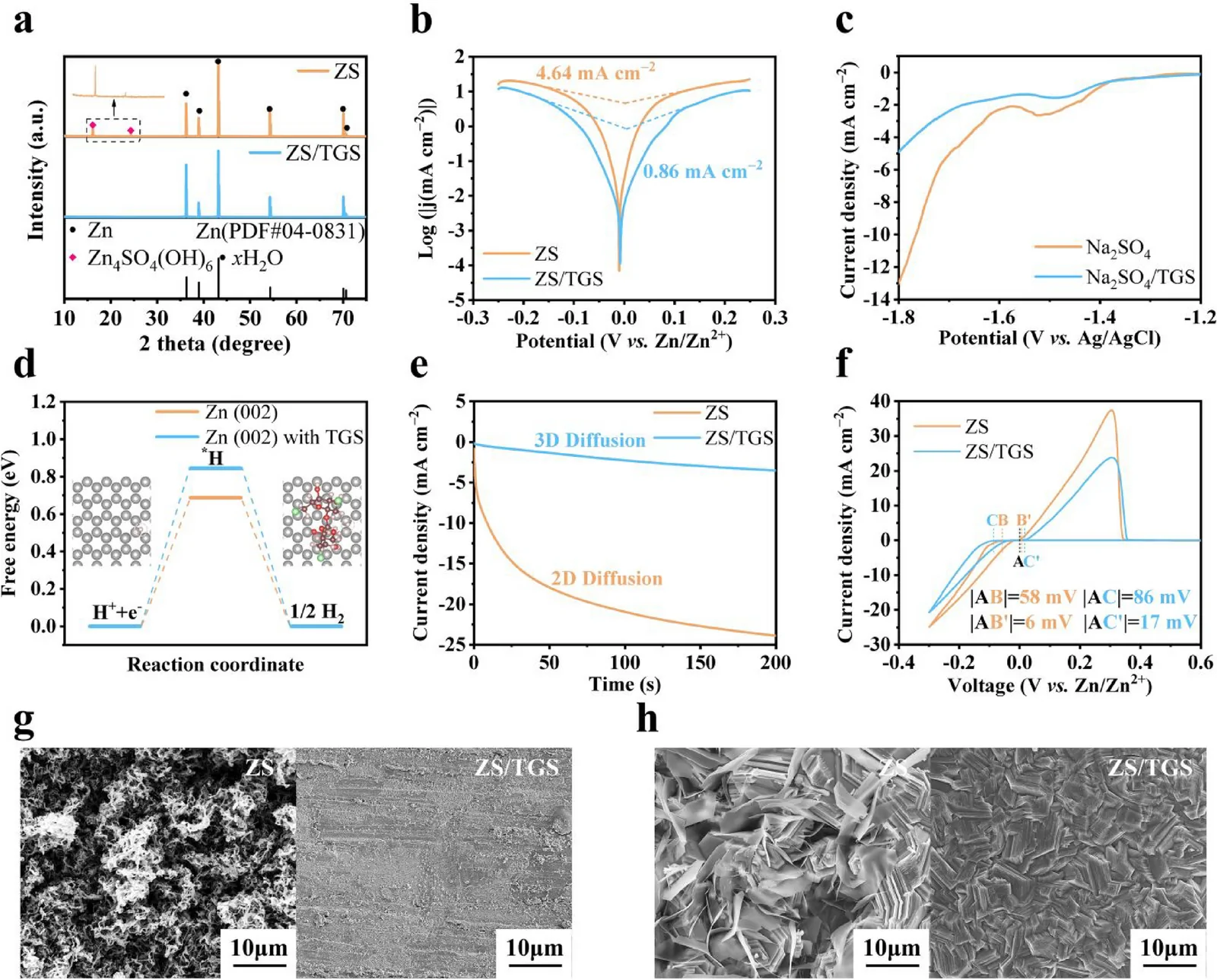
Evaluation of anti-corrosion and deposition kinetics of the Zn anode. **a** XRD patterns of the immersed Zn foils for 3 days, with the ZS sample showing peaks of ZSH corrosion by-products, absent in ZS/TGS. **b** Tafel plots of Zn||Zn symmetric cells. **c** LSV curves of three-electrode beaker cells indicate reduced hydrogen evolution in ZS/TGS compared to ZS. **d** Calculated HER energy barriers showing higher barriers for Zn (002) with TGS. **e** CA curves at an overpotential of −150 mV. **f** CV curves of Zn||Cu asymmetric cells. SEM images of Zn electrodeposited on Zn substrates at **g** 1 mA cm^−2^, 1 mAh cm^−2^ and **h** 4 mA cm^−2^, 2 mAh cm^−2^ in ZS and ZS/TGS electrolytes

Based on the modified EDL structure with the addition of TGS, the corrosion resistance and deposition kinetics of the Zn anode were evaluated experimentally (Fig. 4**)**. The scanning electron microscope (SEM) images and X-ray diffraction (XRD) patterns of the Zn foil soaked in ZS electrolyte for 3 days present obvious corrosion, characterized by randomly distributed irregular ZSH by-products and rough surface morphology, indicative of severe side reactions (Figs. 4a and S36) [103]. In sharp contrast, the Zn anode soaked in ZS/TGS electrolyte for the same condition exhibits a smoother and more uniform surface, without the distinct formation of by-products, confirming enhanced anti-corrosion of the Zn anode conferred by the TGS additive. Accordingly, the Tafel plots show a much lower corrosion current density for the ZS/TGS electrolyte compared to the ZS electrolyte (0.86 *vs.* 4.64 mA cm^−2^, Fig. 4b). The linear sweep voltammetry (LSV) curves also take on a significantly reduced current density under the negative polarization region (Fig. 4c), which is attributed to the amplified energy barrier of HER after TGS adsorption (from 0.69 to 0.84 eV) based on DFT calculations (Fig. 4d). These results demonstrate that a water-poor and anion-expelled EDL constructed by adsorbed TGS would slow down the HER rate and enhance the stability of the Zn anode in a mild/acid aqueous system.

The chronoamperometry (CA) tests are performed to probe into the effect of the modified EDL on Zn^2+^ ion deposition behavior. Different from the random 2D diffusion in the ZS electrolyte, the ZS/TGS electrolyte promotes a uniform 3D diffusion of Zn^2+^ ions at the interface [72, 104], effectively preventing Zn dendritic growth and ensuring a more stable Zn^2+^ ion deposition process (Fig. 4e). As pointed out in the previous theoretical calculations, TGS will inevitably incur a strong steric hindrance effect on the surface, which is confirmed by the enlarged charge transfer resistance (*R*_ct_) in the electrochemical impedance spectroscopy (EIS) spectra shown in Fig. S5 [71–73]. Additionally, the CV curves of Zn||Cu half cells demonstrate that the ZS/TGS electrolyte results in a postponed onset potential of Zn^2+^ ion deposition (Fig. 4f), indicating the significant polarization on the Zn anode. Furthermore, the activation energy (*E*_a_) of the ZS/TGS electrolyte (83.35 kJ mol^−1^) is slightly higher than that of the ZS electrolyte (77.29 kJ mol^−1^) in Figs. S37 and S38, indicating that the presence of adsorbed TGS within the EDL slows Zn^2+^ diffusion at the Zn-electrolyte interface and moderates the Zn^2+^ deposition kinetics [105, 106]. This regulated ion transport promotes a more uniform Zn^2+^ flux, leading to dense and homogeneous Zn deposition. These findings are consistent with the increased interfacial polarization observed in the presence of the TGS additive.

The electrodeposition morphology of Zn in ZS and ZS/TGS electrolytes was systematically analyzed under varying current densities and areal capacities, as depicted in Fig. 4g, h. The overpotential ($\eta_{{\mathrm{C}}}$) governing the electrodeposition process is expressed as [91]:

$$\eta_{{\mathrm{C}}} = \phi_{1} + \frac{2RT}{F}\ln j_{{\mathrm{C}}}$$

where $\phi_{1}$ represents the potential of the diffuse layer, ${\mathrm{R}}$ is the ideal gas constant, ${\mathrm{T}}$ is the absolute temperature, ${\mathrm{F}}$ is the Faraday constant, and ${\mathrm{j}}_{\mathrm{C}}$ is the exchange current density. The repulsion of SO_4_^2−^ ions in HP leads to a redistribution of anions further into the diffuse layer, effectively lowering the potential gradient in this region. As a result, the weaker potential drop in HP gives rise to an increase in the potential of the diffuse layer ($\phi_{1}$) in Fig. S27. The increased $\phi_{1}$ brings about a higher overpotential, which is beneficial to uniform and compact Zn^2+^ ion deposition as it reduces the critical nucleation size and increases nucleation density [71].

At a current density of 1 mA cm^−2^ and an areal capacity of 1 mAh cm^−2^, the electrodeposition morphology in ZS electrolyte exhibits a rough and porous mossy structure, indicative of severe HER and dendrite growth (Fig. 4g) [107]. The morphology of initial Zn nucleation critically influences the subsequent growth behavior, playing a pivotal role in dendrite formation and ultimately affecting the long-term cycling stability and lifespan of the battery [108, 109]. This irregular morphology can compromise the stability of the Zn anode and lead to a rapid short circuit because of strong EDL repulsion according to DLVO theory. In contrast, the Zn anode in the ZS/TGS electrolyte takes on a much smoother and more uniform surficial morphology under identical conditions, as exhibited due to the vdW force domination. At a higher current density of 4 mA cm^−2^ and an areal capacity of 2 mAh cm^−2^, the Zn^2+^ ion deposits in the ZS electrolyte display pronounced dendritic structures with large sharp-edge plates, while remaining smooth and compact in the ZS/TGS electrolyte, with a well-defined and uniform crystalline structure (Fig. 4h). Additional electrodeposition morphologies at higher current densities and areal capacities (5 mA cm^−2^, 2.5 mAh cm^−2^; 5 mA cm^−2^, 5 mAh cm^−2^) are presented in Figs. S39 and S40, which reveal similar trends. Accordingly, it is reasonable to claim that incorporating TGS into the ZS electrolyte moderates the Zn^2+^ ion deposition kinetics, leading to a homogeneous and dense growth of electrodeposition.

### Modified EDL Induced Superior Electrochemical Performance

**Fig. 5:**
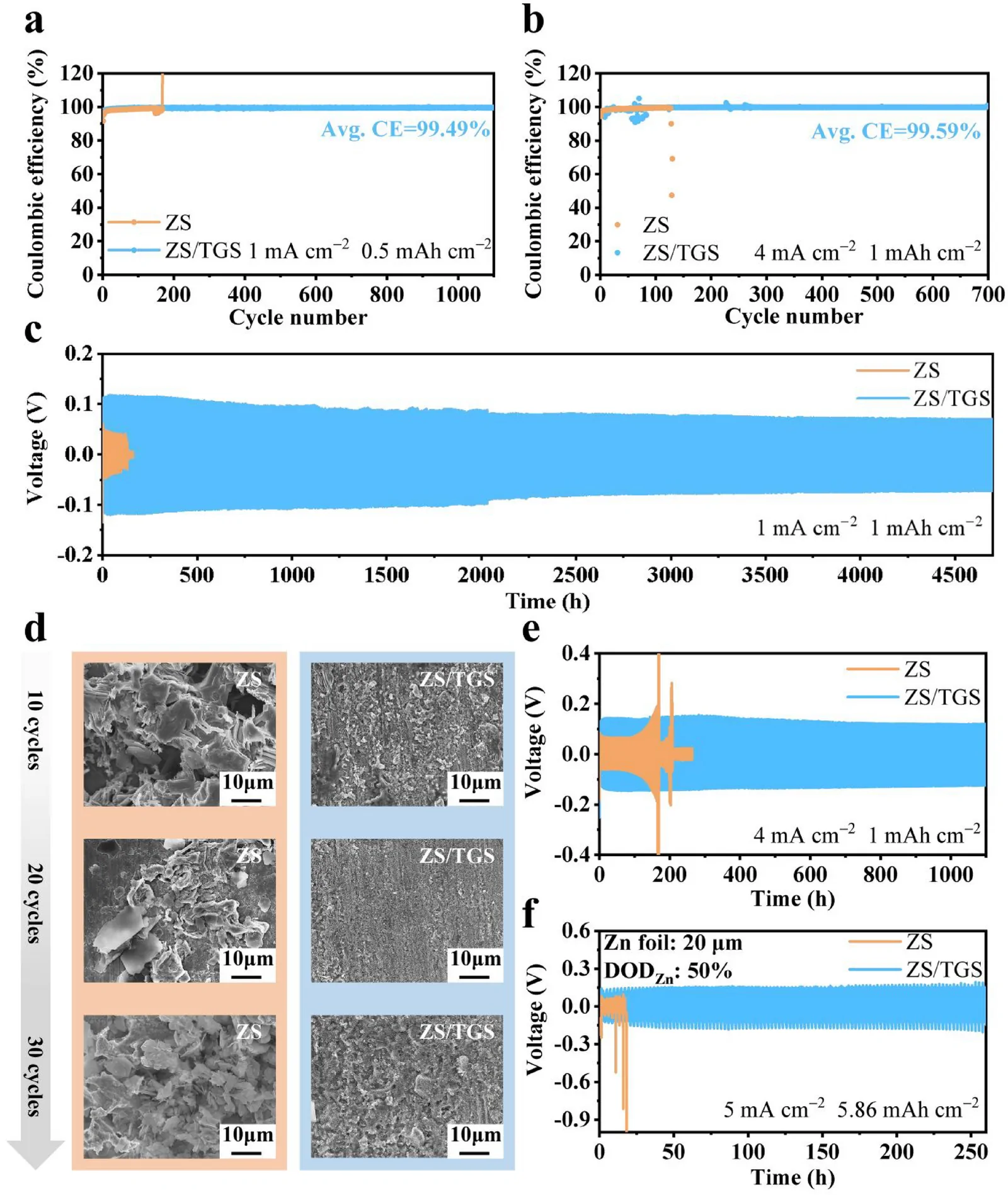
Reversibility evaluation of Zn anode. CE comparisons of Zn||Cu asymmetric cells at **a** 1 mA cm^−2^, 0.5 mAh cm^−2^ and **b** 4 mA cm^−2^, 1 mAh cm^−2^. **c** Voltage profiles of Zn||Zn symmetric cells galvanostatically cycled at 1 mA cm^−2^, 1 mAh cm^−2^, showing stable cycling in ZS/TGS over 4700 h. **d** SEM images of cycled Zn foils after 10, 20, and 30 cycles (1 mA cm^−2^, 1 mAh cm^−2^) in ZS (left, orange background) and ZS/TGS (right, blue background) electrolytes. Voltage profiles of Zn||Zn symmetric cells galvanostatically cycled at **e** 4 mA cm^−2^, 1 mAh cm^−2^ and at **f** 5 mA cm^−2^, 5.86 mAh cm^−2^ (DOD_Zn_ = 50%)

We then assessed the reversibility of the Zn anode in ZS and ZS/TGS electrolytes by measuring the CE in Zn||Cu asymmetric cells. As shown in Fig. 5a, at a current density of 1 mA cm^−2^ and an areal capacity of 0.5 mAh cm^−2^, the Zn||Cu cell with ZS electrolyte operates only 200 cycles and then reaches a short circuit. In sharp contrast, the cell utilizing ZS/TGS electrolyte exhibits stable cycling for over 1100 cycles, achieving an impressive average CE of 99.49%. Even at a higher current density of 4 mA cm^−2^ and an areal capacity of 1 mAh cm^−2^, the cell with ZS/TGS electrolyte can still stably cycle for up to 700 cycles with a superior CE of 99.59% (Fig. 5b), observably outperforming that of the cell with ZS electrolyte. The promotion in CE embodies optimized Zn plating/stripping reversibility achieved by TGS-regulated EDL structure. To further verify the long-term cycling stability of the Zn anode, we perform the galvanostatic charge/discharge tests of Zn||Zn symmetric cells in both electrolytes. As shown in Fig. 5c, the Zn anode in the ZS/TGS electrolyte demonstrates significantly prolonged cycling stability for over 4700 h compared to only 140 h in the ZS electrolyte, under a current density of 1 mA cm^−2^ and an areal capacity of 1 mAh cm^−2^. To gain deeper insights into the morphological evolution of the Zn anode, the Zn anodes cycled for 10, 20, and 30 cycles in ZS and ZS/TGS electrolytes at the same condition are characterized by SEM (Fig. 5d). The Zn anode cycled in ZS/TGS electrolyte delivers a uniform and smooth Zn plating/stripping process, further confirming the effect of EDL reconstruction in facilitating the homogeneous Zn^2+^ ion deposition by amplifying the overpotential (~ 100 mV at 1 mA cm^−2^ and 1 mAh cm^−2^). However, in the ZS electrolyte, the cycled Zn anode exhibits non-uniform plating/stripping, inducing noticeable voids and randomly distributed Zn sheets. Moreover, similar results are shared by the Zn anodes cycled under other conditions, as presented in Figs. S41-S43. The XRD patterns in Fig. S44 further confirm the formation of ZSH after 30 cycles at 1 mA cm^−2^ and 1 mAh cm^−2^ in the ZS electrolyte. Optical photographs of the ZS electrolyte post-cycling reveal noticeable dead Zn, while the ZS/TGS electrolyte remains transparent (Fig. S44b, c). At a higher current density of 4 mA cm^−2^, the Zn anode in the ZS/TGS electrolyte still operates stably for up to 1100 h, approximately six times longer than that in the ZS electrolyte (~ 180 h, Fig. 5e). Additional long-term cycling tests at various conditions (4 mA cm^−2^ and 2 mAh cm^−2^; 5 mA cm^−2^ and 1 mAh cm^−2^) also demonstrate the superior performance of the ZS/TGS electrolyte (Fig. S45). To evaluate the rate performance of symmetric cells, Zn||Zn cells were tested at current densities ranging from 0.5 to 10 mA cm^−2^, as shown in Fig. S46. As expected, the cell using the ZS electrolyte experienced a short circuit when the current density was returned to 0.5 mA cm^−2^. In contrast, the cell with the ZS/TGS electrolyte maintained stable cycling across all current densities, with a noticeably increased overpotential compared to the ZS electrolyte. In the under-limiting current region, the cells can operate safely without reaching the limit of mass transfer. In this regime, nucleation theory plays a dominant role, higher overpotential, along with the increased interfacial impedance observed for the ZS/TGS electrolyte, facilitates more stable Zn deposition and suppresses side reactions [71, 110]. Impressively, under a harsh circumstance of 50% Zn utilization rate (depth of discharge, DOD_Zn_), the Zn||Zn symmetric cell with ZS/TGS electrolyte shows extended lifespan to 260 h (Fig. 5f). In comparison, the Zn anode in the ZS electrolyte suffers from uneven nucleation and uncontrolled Zn/Zn^2^⁺ redox kinetics, accompanied by serious side reactions, leading to rapid active Zn consumption and perforation of the electrode, ultimately causing the cell to reach an open circuit within just 15 h. Finally, the cumulative plating capacity (CPC) at various current densities, 2.35 Ah cm^−2^ over 4700 h at 1 mA cm^−2^, 2.2 Ah cm^−2^ over 1100 h at 4 mA cm^−2^, and 1.65 Ah cm^−2^ over 660 h at 5 mA cm^−2^, demonstrates the superior performance of our system, surpassing most previously reported additive-based strategies (Fig. S47).

**Fig. 6:**
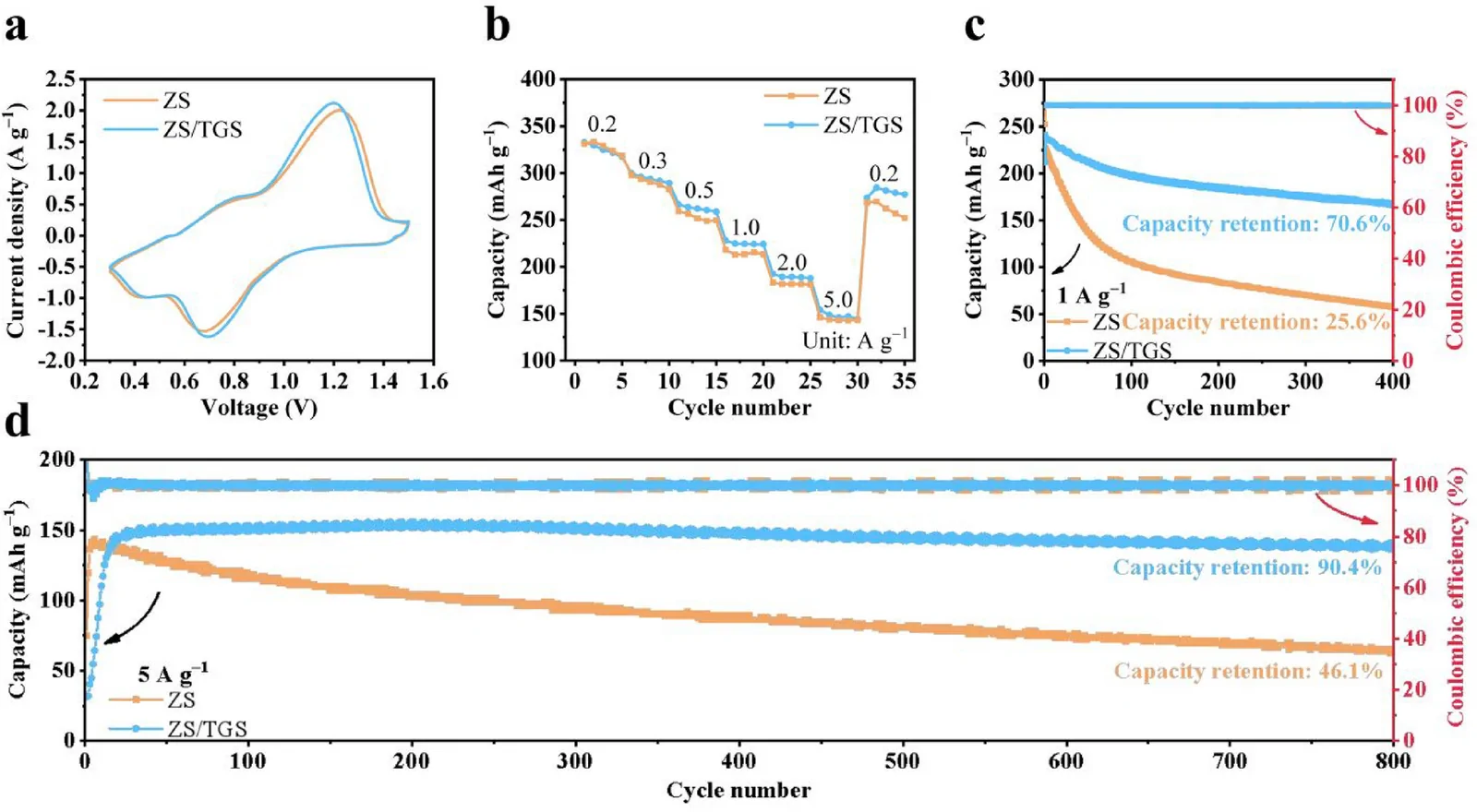
Electrochemical performance of Zn||NVO full cells in different electrolytes. **a** CV curves of Zn||NVO full cells. **b** Rate performance at varying current densities (initial five cycles excluded from rate analysis due to the activation process). Long-term cycling performance of Zn||NVO at **c** 1 A g^−1^ and **d** 5 A g^−1^, indicating superior durability and reversibility in the ZS/TGS electrolyte system

To assess the practicality of regulating the EDL in real-world applications, Zn||NaV_3_O_8_∙1.5H_2_O (NVO) full cells were assembled. The SEM image and XRD pattern of the NVO powders (Figs. S48 and S49) confirm a nanobelt morphology and a highly crystalline phase, which can be indexed to the P2_1_/m space group (PDF#16-0601). EIS results for Zn||NVO full cells (Fig. S50) reveal a charge transfer resistance comparable to that observed in symmetric cells, which is consistent with the previous analysis of interfacial reaction kinetics. The CV profiles of Zn||NVO full cells with different electrolytes (Fig. 6a) reveal similar redox peaks, indicating that TGS primarily functions on the electrode surface without directly participating in the redox reactions of the NVO cathode during charge and discharge processes. This confirms that the improvements in cell performance originate from the regulated EDL rather than changes in the cathode redox chemistry. Fig. 6b showcases the rate performance of the full cells. At low current densities (0.2 and 0.3 A g^−1^), both ZS and ZS/TGS cells exhibit comparable capacities, as side reactions associated with vanadium dissolution from the cathode remain negligible in both systems. However, as cycling progresses under higher current densities (0.5-5 A g^−1^), the ZS electrolyte suffers from persistent side reactions that progressively degrade the NVO cathode, leading to diminished capacity and inferior rate performance. In contrast, the ZS/TGS full cell consistently delivers higher capacity and superior rate capability, highlighting the effectiveness of the TGS-modulated interfacial environment. This enhancement is attributed to the preferential adsorption of TGS on both the anode and cathode surfaces, where it facilitates the formation of a “water-poor and anion-expelled” EDL. By suppressing water insertion and anion accumulation at the interface, vanadium dissolution is significantly mitigated, thereby preventing the formation of inactive by-products and enabling more stable electrochemical kinetics [111, 112]. Long-term cycling performance at 1 A g^−1^ (Fig. 6c) highlights the differences between the ZS and ZS/TGS systems. The ZS-based full cell undergoes an activation process within the first 2 cycles, reaching a peak specific capacity of 226 mAh g^−1^ before rapidly declining to 25.6% of the initial capacity after 400 cycles due to the severe side reactions at low rates [20]. In contrast, the ZS/TGS-based full cell retains 70.6% of its original capacity (168 mAh g^−1^) after 400 cycles, manifesting the stability provided by the TGS adsorption-regulated EDL structure. At a higher rate of 5 A g^−1^ (Fig. 6d), a longer activation process, typically spanning 5 to 30 cycles, is required. This behavior is attributed to intensify interfacial polarization, elevated Zn^2+^ flux, and increased overpotential, all of which collectively delay the formation of a stable EDL and uniform Zn deposition. Nevertheless, the ZS/TGS-based full cell demonstrates excellent long-term stability, retaining 90.4% of its capacity and delivering 141 mAh g^−1^ even after 800 cycles. By contrast, the ZS-based cell suffers from a drastic decline in specific capacity to 46.1% within 800 cycles, primarily due to irreversible capacity loss caused by severe side reactions. To evaluate practical applicability, a single-layer Zn||NVO pouch cell with cathode mass loadings of 20 mg cm^−2^ was assembled, as illustrated in Fig. S51. The 0.24 Ah single-layer pouch cell utilizing the TGS-containing electrolyte demonstrates an extended cycling lifespan of over 50 cycles at a small current density of 0.1 A g^−1^ (2 mA cm^−2^), retaining 71.8% of its initial capacity.

## Conclusions

This study establishes a multiscale computational framework integrating QC, DFT, and CMD to comprehensively elucidate the interfacial mechanisms governing the stability and reversibility of Zn anodes in ARZBs. By synergistically bridging ab initio molecular-level insights with experimental validation, this framework resolves the dynamic evolution of the EDL under operational conditions, offering unprecedented clarity on the interplay between interfacial chemistry and electrochemical performance. QC simulations identified critical adsorption sites on Zn surfaces, while DFT calculations quantified the energetics and configurations of adsorbate interactions, revealing the preferential adsorption of TGS molecules. CMD simulations further uncovered the EDL’s structural reorganization under realistic electrolyte conditions, demonstrating how TGS disrupts the native water-rich and anion-dense interfacial environment. The combined theoretical analyses established that TGS adsorption induces steric hindrance and site-specific interactions, effectively expelling free water and anions from the EDL. This restructuring suppresses parasitic reactions (e.g., hydrogen evolution, by-product formation) and promotes uniform Zn^2+^ ion deposition by homogenizing interfacial ion flux. Experimental validation corroborated these theoretical predictions: Zn||Zn symmetric cells with TGS-modified electrolytes achieved ultralong cycling stability (> 4700 h at 1 mA cm^−2^/1 mAh cm^−2^), while Zn||Cu asymmetric cells exhibited a superior CE (99.49% over 1100 cycles at 1 mA cm^−2^/0.5 mAh cm^−2^). Full cells paired with NVO cathodes retained 90.4% capacity retention after 800 cycles at 5 A g^−1^, underscoring the practical viability of the TGS additive. These results not only validate the predictive power of the multiscale framework, but also highlight its utility in guiding the rational design of electrolyte additives for next-generation ARZBs.

## Supplementary Information

Below is the link to the electronic supplementary material.

Supplementary file1 (DOCX 62834 KB)
