# Supplementary material for: Multiscale Theoretical Calculations Empower Robust Electric Double Layer Toward Highly Reversible Zinc Anode
Source: Nanomicro Lett. 2025 Dec 25;18:90. doi: 10.1007/s40820-025-01915-w (PMC12738512; doi:10.1007/s40820-025-01915-w)
Supplement: Supplementary file 1 — Supplementary file1 (DOCX 62834 KB) [file 40820_2025_1915_MOESM1_ESM.docx]

Supporting Information for

**Multiscale Theoretical Calculations Empower Robust Electric Double Layer Toward Highly Reversible Zinc Anode**

Yufan Xia^1,†^, Zhen Luo^1,†^, Shuang Chen^1^, Yang Xiang^1^, Gao Weng^1^, Hongge Pan^1,3^, Ben Bin Xu^4^, Mi Yan^1^, and Yinzhu Jiang^1,2,^***

^1^School of Materials Science and Engineering, Zhejiang University, Hangzhou 310027, P. R. China

^2^Future Science Research Institute, ZJU-Hangzhou Global Scientific and Technological Innovation Center, Zhejiang University, Hangzhou 311215, P. R. China

^3^Institute of Science and Technology for New Energy, Xi'an Technological University, Xi'an 710021, P. R. China

^4^Mechanical and Construction Engineering, Faculty of Engineering and Environment, Northumbria University, Newcastle upon Tyne, NE1 8ST, United Kingdom

^†^Yufan Xia and Zhen Luo equally contributed to this work.

***Corresponding authors. E-mail: [yzjiang@zju.edu.cn](mailto:yzjiang@zju.edu.cn) (Yinzhu Jiang)

**S1 Computational Methods and Experimental Section**

**S1.1 Joint Density Functional Theory Calculations**

Joint Density Functional Theory (JDFT) calculations were performed using the JDFTx software [S1], an open-source tool specifically designed for JDFT computations. GBRV pseudopotentials [S2] were employed for the Zn species, with exchange-correlation interactions described by the GGA-PBE functional [S3]. Electronic structure calculations utilized an energy cutoff of 20 Ry for wavefunctions and 100 Ry for charge density. The Brillouin zone was sampled using a k-point grid of 12 × 12 × 1 for the Zn (002) surface, and electronic smearing was applied via the Fermi method with a smearing width of 0.01 Ry.

The fluid environment was modeled using the LinearPCM approach, specifically the CANDLE variant [S4, S5], with water serving as the solvent and Na^+^ and F^−^ as the cation and anion in the continuum aqueous medium, respectively. This comprehensive setup enables accurate modeling of the electrochemical system, effectively capturing the complex interactions between the solid-state and solvent environments at both molecular and atomic levels.

Voltages of +2 V and −2 V relative to the PZC were applied, based on the results from JDFT calculations regarding surface charge density. As shown in Fig. S31, the surface charge varies quasi-linearly with applied potential, spanning from approximately −54.54 μC cm^−2^ at −2 V to +26.24 μC cm^−2^ at +2 V. Specifically, potentials of −0.5 V and +0.5 V relative to the PZC correspond to surface charges of −7.97 and +5.98 μC cm^−2^, respectively. To further elucidate the behavior of the Zn anode-electrolyte interface under both positive and negative polarization conditions, voltages of +0.5 V and −0.5 V were also considered, corresponding to a loss or gain of approximately 0.009174 and 0.012252 electrons, respectively, by each Zn atom on the Zn (002) surface. These calculations establish a foundational framework for subsequent simulations at various voltages, both in the presence and absence of TGS molecules.

**S1.2 Materials**

Zinc sulfate heptahydrate (ZnSO_4_·7H_2_O, AR), zinc foil (99.999%), and sodium chloride (NaCl, AR) were purchased from Sinopharm Chemical Reagent Co., Ltd. Sodium sulfate (Na_2_SO_4_, AR) was obtained from Shanghai Macklin Biochemical Technology Co., Ltd. Vanadium pentoxide (V_2_O_5_, 99.5%), 4,1',6'-trichlorogalactosucrose (C_12_H_19_Cl_3_O_8_, TGS, 98%), and N-methyl-2-pyrrolidone (NMP, 99.9%) were acquired from Aladdin. The separator used in the battery was glass microfiber (GF/D) purchased from Whatman. Super P carbon and poly(vinylidene fluoride) (PVDF) were procured from Switzerland Timcal and Sigma-Aldrich, respectively.

**S1.3 Electrolyte preparation and battery assembly**

2 M ZnSO_4_ (ZS) was prepared as the baseline electrolyte. The comparison electrolyte was obtained by adding 10, 50, and 100 mM TGS into the 2 M ZnSO_4_ solution and stirring until it completely dissolved. 50 mM TGS additive exhibits the best cycling life, denoted as ZS/TGS. The electrolytes of 1 M Na_2_SO_4_ and 1 M Na_2_SO_4_/TGS were used in the hydrogen evolution performance. Ultrapure water was used to prepare all electrolytes.

Zn||Zn symmetric cells were assembled in CR2025-type coin cells in the atmosphere, with Zn foils as both the anode and cathode. The assembling processes of Zn||Cu asymmetric cells and Zn||NaV_3_O_8_·1.5H_2_O (NVO) full cells were the same as Zn||Zn symmetric cells, except that the cathodes were changed to Cu foil and NVO, respectively. The pouch cell was assembled with one 8 × 5 cm^2^ NVO cathode, one 8 × 5 cm^2^ anode (thickness: 100 μm), and one 9 × 6 cm^2^ separator.

**S1.4 Synthesis of NVO cathode**

The NVO powder was synthesized according to a reported facile solution reaction method. Typically, V_2_O_5_ (2 g) was dissolved into 30 mL of 2 M NaCl aqueous solution. After violently stirring for 96 h at 30 ℃, the suspension was thoroughly washed with deionized water several times. Finally, the black-red product was obtained by drying at 80 ℃ for 12 h in air.

**S1.5 Materials characterizations**

The electrolytes were characterized by Fourier transform infrared spectroscopy (FTIR, Thermo Scientific Nicolet iS20), Raman spectroscopy (Raman, HORIBA Scientific LabRAM HR Evolution, 532 nm laser), and nuclear magnetic resonance spectrometer (^2^H NMR, Bruker 400 MHz). The morphology of the Zn anodes was identified by scanning electron microscope (SEM, JSM-IT800, Japan), with an operation voltage of 20 kV and operation current of 75 μA under the SHL mode with a UHD detector. The working distance (WD) was set as 8 mm. X-ray diffraction (XRD) patterns of electrodes were conducted using a Bruker D8 diffractometer with a scanning range of 10° to 90°. X-ray photoelectron spectroscopy (XPS) was obtained by Thermo Scientific K-Alpha equipped with an Al K*α* X-ray source (12 kV, 6 mA).

**S1.6 Electrochemical measurements**

All fabricated cells were tested by the Neware BTS-5 test system after resting for 6 h. The NVO cathode was prepared by mixing NVO powder, Super P carbon, and PVDF with a mass ratio of 7:2:1 in 700 μL NMP and then casting onto Ti foil (10 μm) with a doctor blade. The cathode was dried at 80 ℃ for over 12 h in a vacuum oven with an average mass loading of 5 mg cm^–2^. All the electrodes of the coin cell were cut into disks with a diameter of 16 mm.

Cyclic voltammetry (CV), linear polarization tests, linear sweep voltammetry (LSV), chronoamperometry (CA), and electrochemical impedance spectroscopy (EIS) were performed on the CHI760E electrochemical workstation. The CV curves of Zn||Zn cells were carried out with different scan rates (from 2-10 mV s^–1^) between –15 to 15 mV, whereas those of Zn||Cu cells were at 2 mV s^–1^ between –0.3 to 0.6 V. The Zn||NVO full cells were cycled between 0.3-1.5 V at a scan rate of 0.2 mV s^–1^. Linear polarization tests were performed at a scan rate of 0.01 V s^–1^ using Zn||Zn cells. LSV results were recorded with a three-electrode beaker cell, where Zn foil is the working electrode, Ti foil is the counter electrode, and Ag/AgCl is the reference electrode. CA curves were measured at a fixed overpotential of –150 mV. EIS spectra were conducted with a frequency range between 0.01-10^6^ Hz. Zeta potential measurements were performed using a Zetasizer Nano ZS, Malvern Instruments. The zeta potentials of the Zn anode in different electrolytes were derived from the electrophoretic mobility based on the Smoluchowski formula. All measurements were averaged over two individually performed experiments and conducted in triplicate for each sample.

The ionic conductivity ($\text{σ}$) of different electrolytes was calculated by:

$$\text{σ}\text{=}\frac{\text{L}}{\text{SR}}$$

where $\text{L}$ and $\text{S}$ denote the distance between the two carbon-coated platinum electrodes and the surface area of the working electrode, respectively. The bulk resistance $\text{R}$ is determined from the high-frequency intercept of the EIS spectrum. Given that the conductivity ($\text{σ}$) and resistance ($\text{R}$) of a 3 M KCl standard solution at 25 ℃ are 86.22 mS cm^−1^ and 12.88 Ω, respectively, the conductivity of the testing solution can be calculated using the above equation by substituting the corresponding measured resistance value.

The activation energy ($\text{E}_{\text{a}}$) can be quantitatively evaluated based on the temperature-dependent EIS, as described by the Arrhenius equation:

$$\frac{\text{1}}{\text{R}_{\text{ct}}}\text{=}\text{A}\text{e}^{\frac{\text{-}\text{E}_{\text{a}}}{\text{RT}}}$$

where $\text{R}_{\text{ct}}$ represents the charge-transfer resistance, $\text{A}$ is the pre-exponential (frequency) factor, $\text{R}$ is the universal gas constant, and $\text{T}$ is the absolute temperature.

**S2 Supplementary Figures**


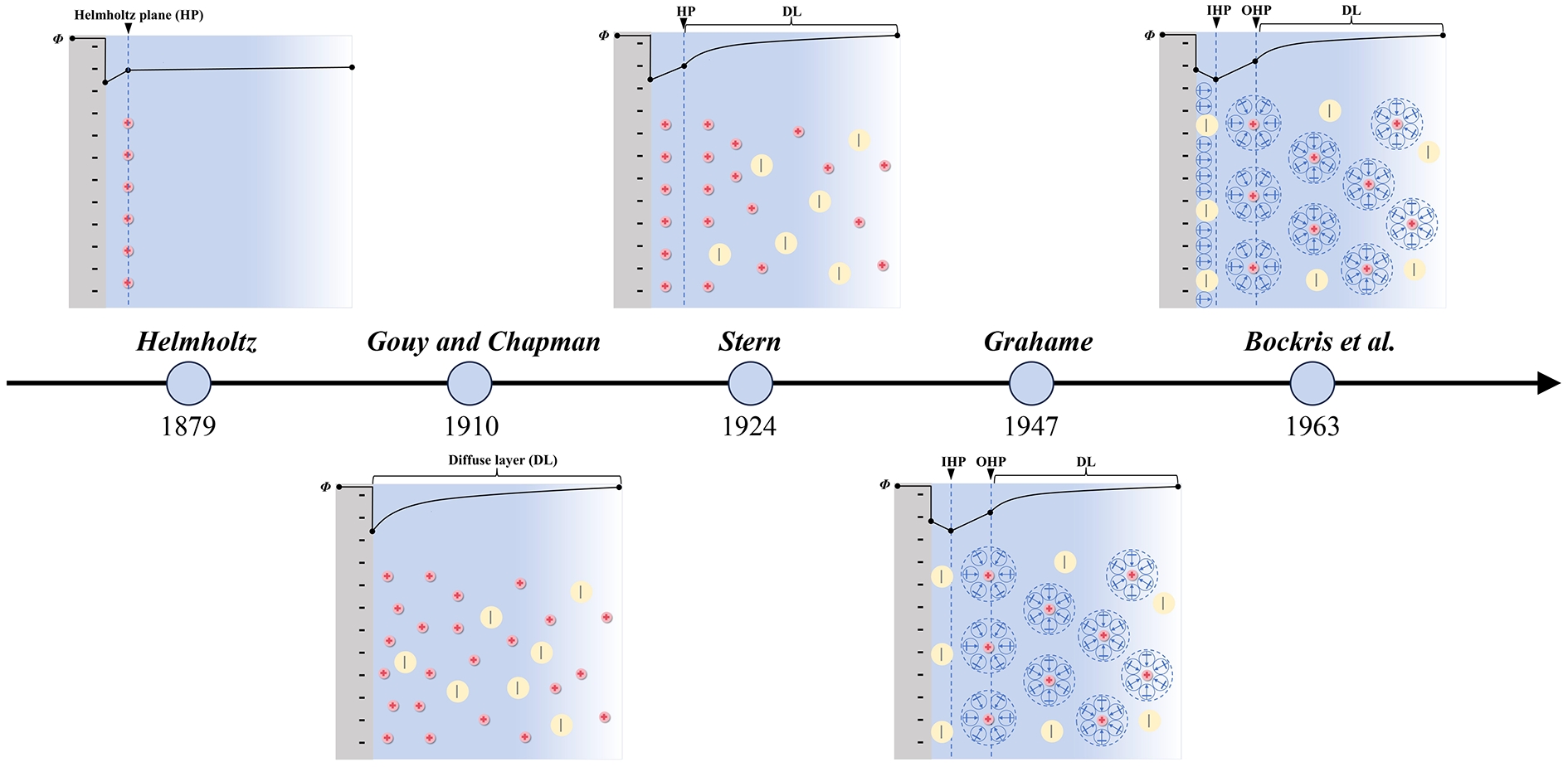


**Fig. S1** The history of the development of the classic EDL model


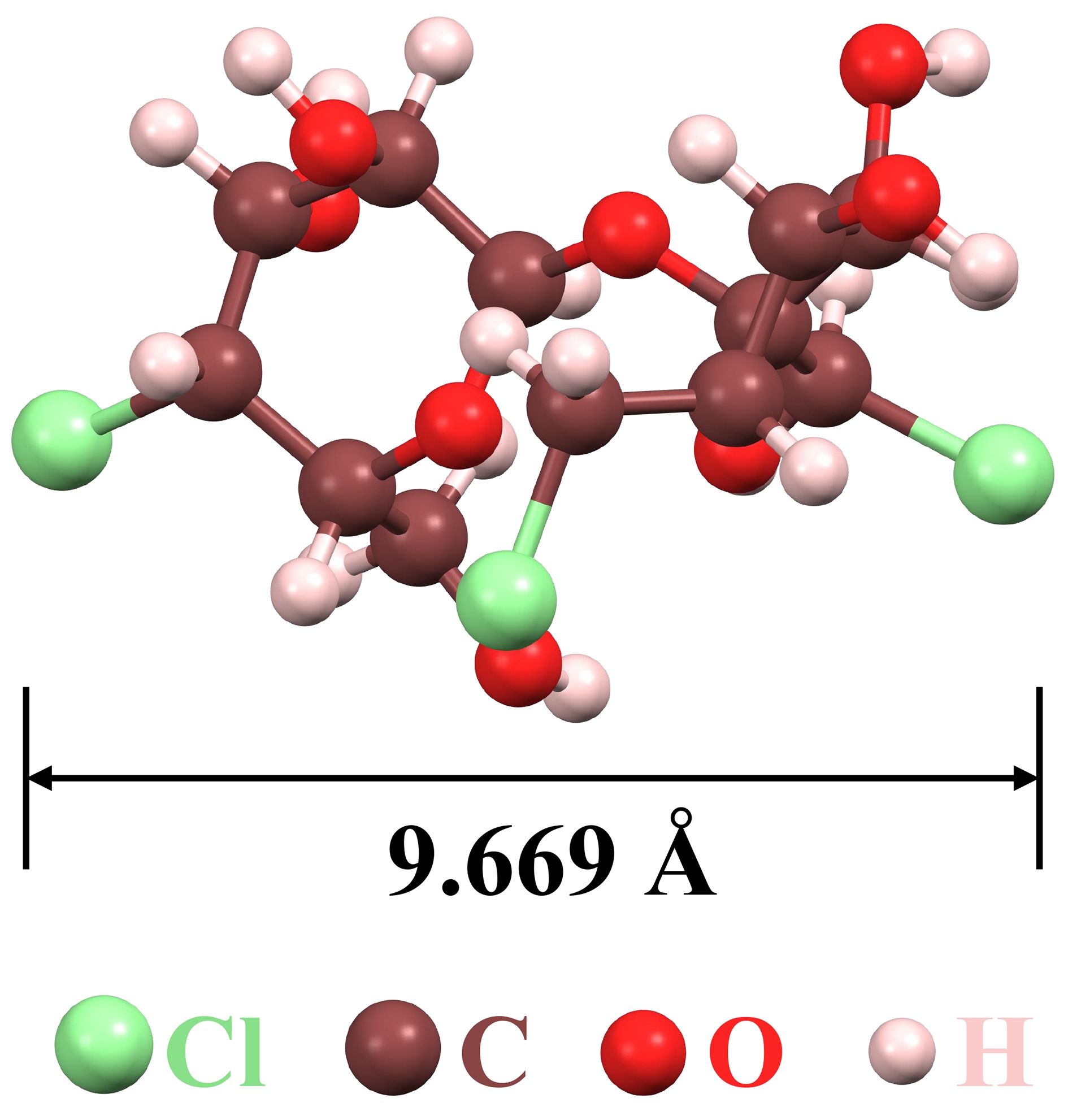


**Fig. S2** The molecular structure of TGS


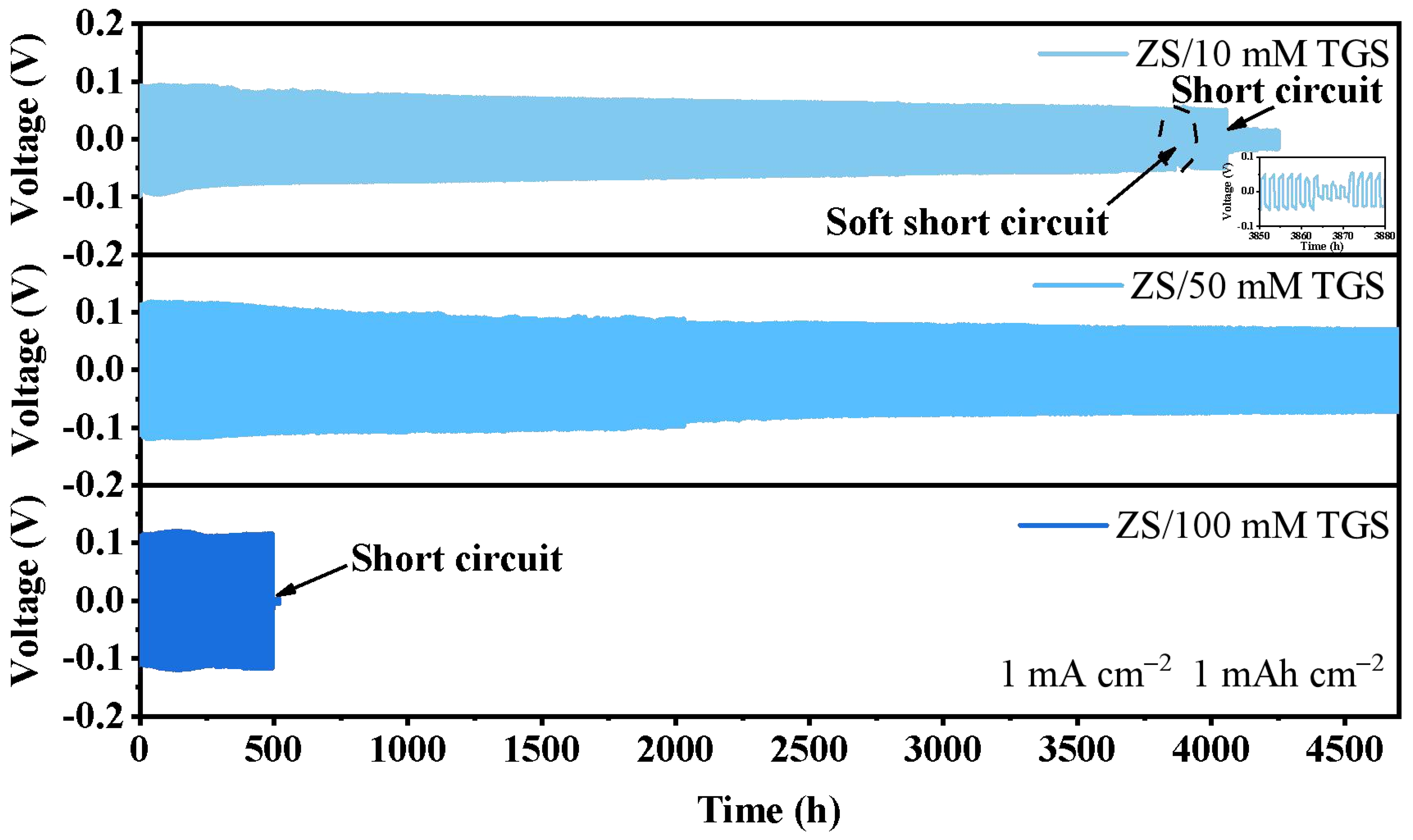


**Fig. S3** Voltage profiles of Zn||Zn symmetric cells during galvanostatic cycling at a current density of 1 mA cm^−2^ and an areal capacity of 1 mAh cm^−2^, comparing the effects of different TGS concentrations (10 mM, 50 mM, and 100 mM). The cell with 10 mM TGS experiences a soft short circuit followed by a complete short circuit, while the cell with 100 mM TGS short-circuits after approximately 500 h. In contrast, the cell with 50 mM TGS demonstrates superior stability for the whole 4700 h. These results highlight 50 mM TGS as the optimal concentration for achieving the best cycling stability in Zn||Zn symmetric cells


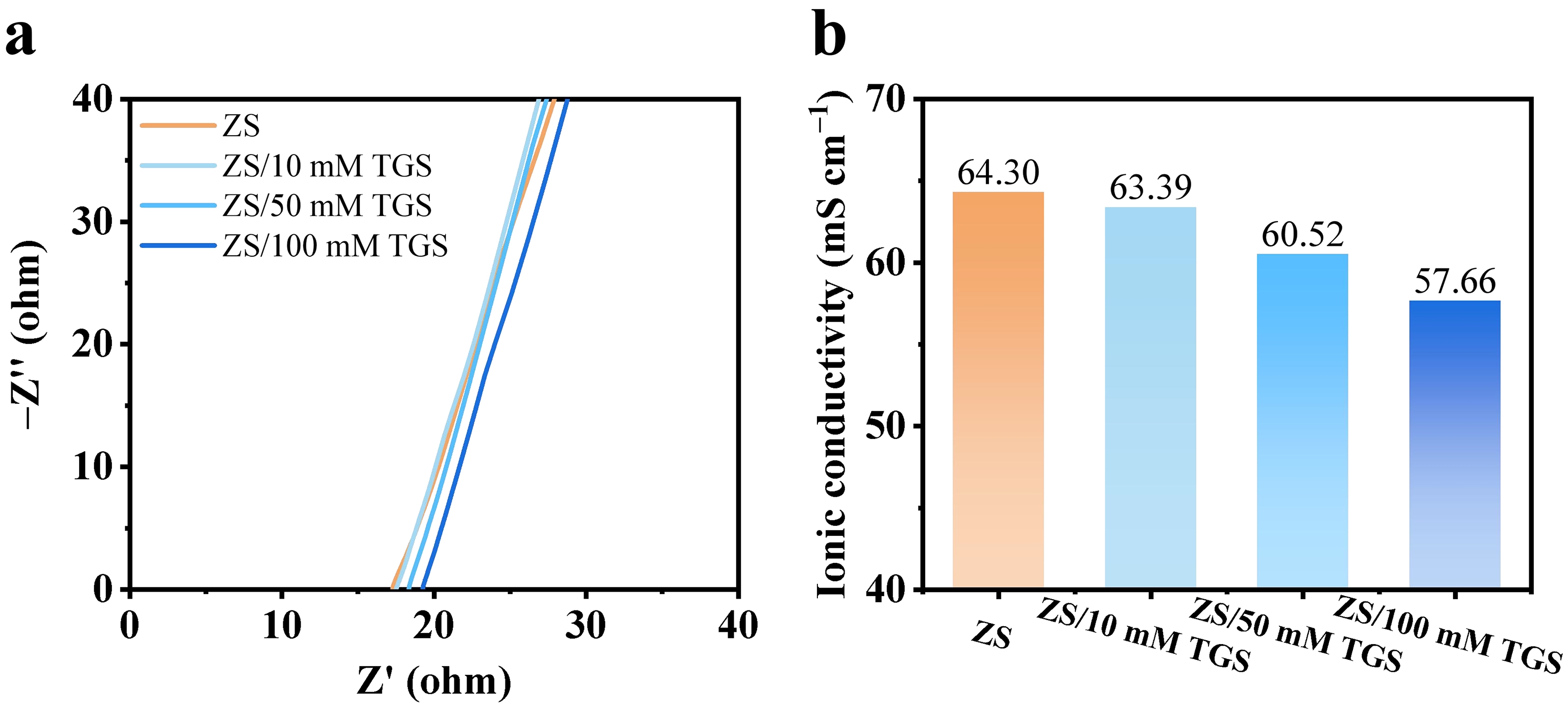


**Fig. S4 a** EIS spectra and **b** ionic conductivities of ZS, ZS/10 mM TGS, ZS/50 mM TGS, and ZS/100 mM TGS electrolytes


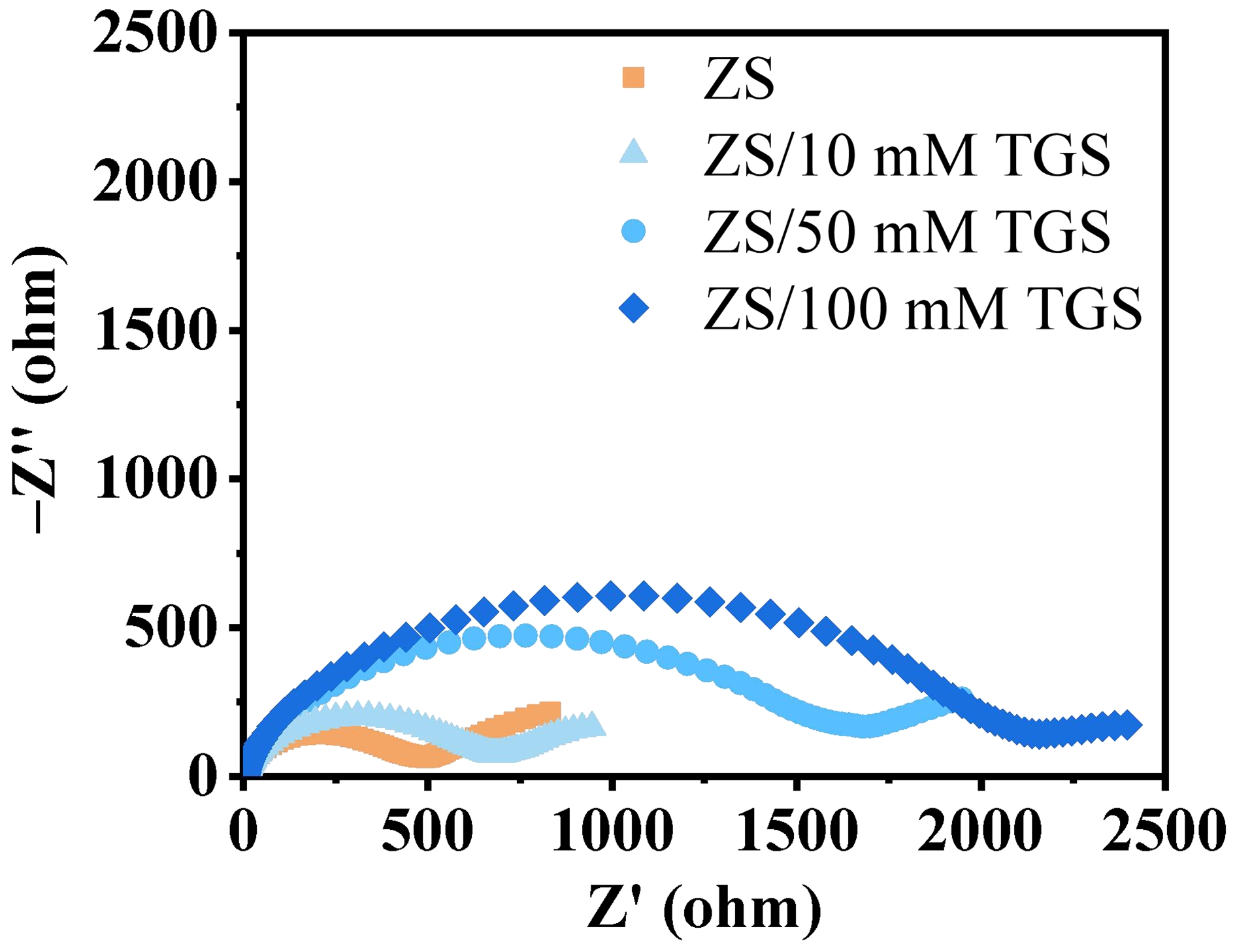


**Fig. S5** EIS spectra of Zn||Zn symmetric cells in ZS, ZS/10 mM TGS, ZS/50 mM TGS, and ZS/100 mM TGS electrolytes at 298.15 K


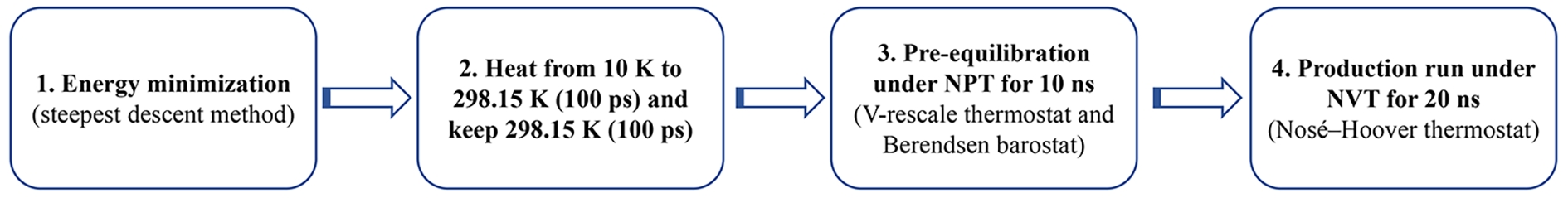


**Fig. S6** Workflow of the classical molecular dynamics (CMD) simulations for the bulk electrolyte system and this comprehensive process ensures the system reaches equilibrium and generates reliable simulation data


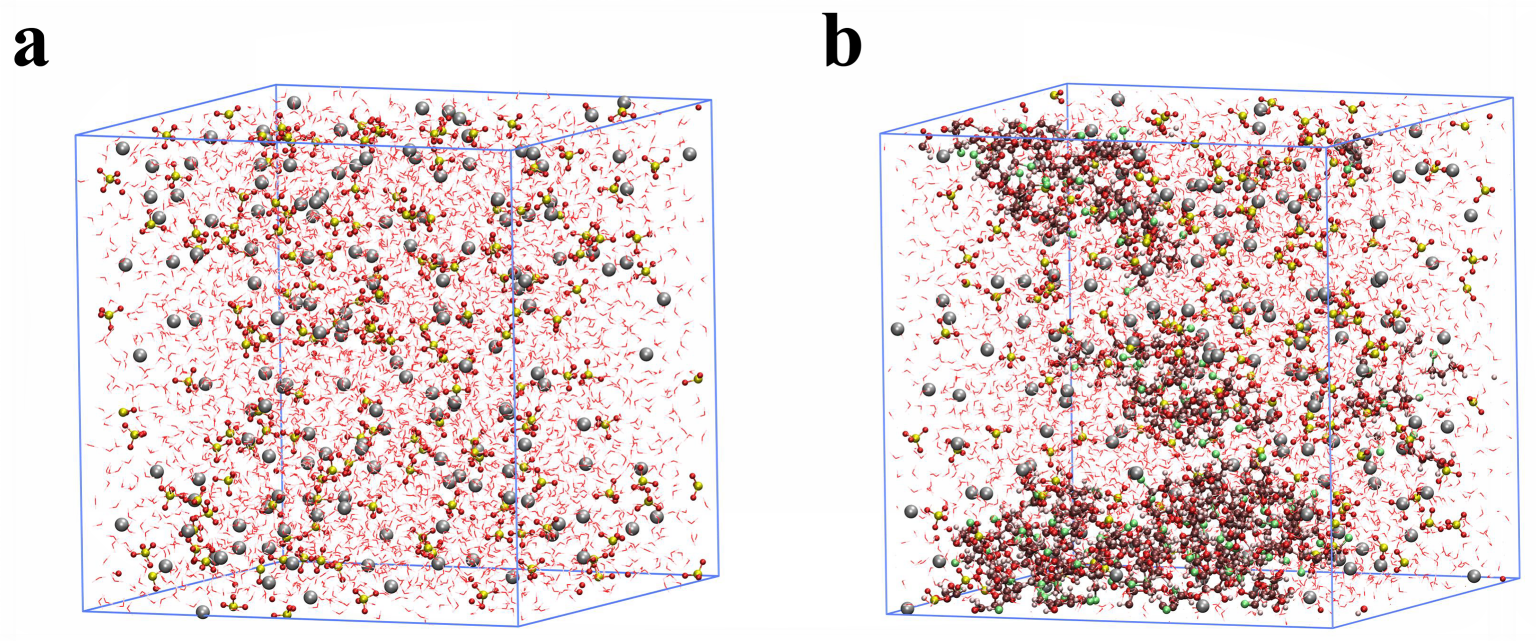


**Fig. S7** MD simulation snapshots of **a** ZS electrolyte and **b** ZS/TGS electrolyte. In Fig. S7a, the sulfate ions are uniformly distributed, confirming the accuracy of the simulation and avoiding artifacts caused by inaccurate charge representations. In Fig. S7b, a noticeable aggregation of TGS molecules is observed, indicating their clustering within the electrolyte and the non-negligible steric hindrance effect


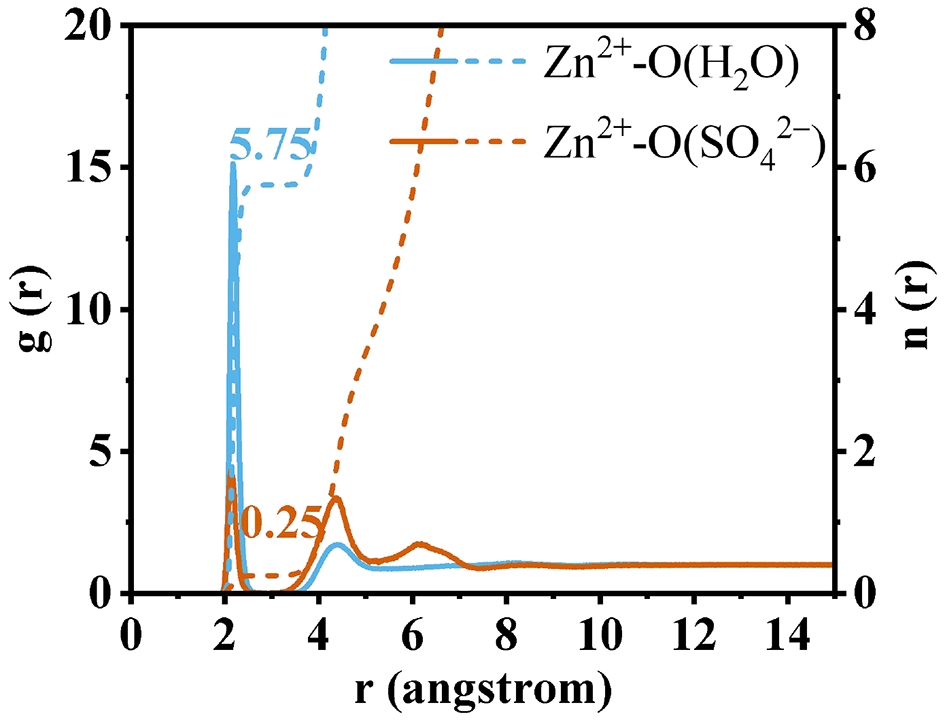


**Fig. S8** Radial distribution functions (RDFs) and coordination numbers (CNs) analysis of bulk ZS electrolyte. The coordination number of Zn^2+^ ions is calculated to be approximately 6, confirming the high accuracy of the molecular dynamics simulations


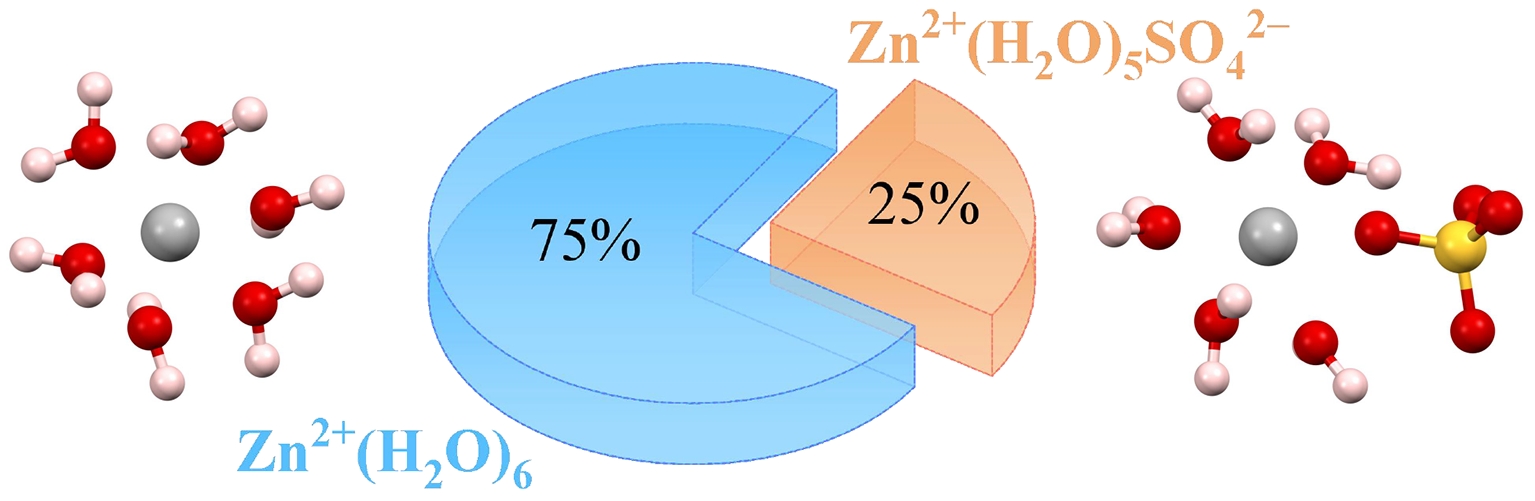


**Fig. S9** Solvation structures of Zn^2+^ ions in 2M ZnSO_4_ electrolytes. The pie chart illustrates the fractional distribution of solvation structures, with ~75% of Zn^2+^ ions existing as Zn^2+^(H_2_O)_6_ and ~25% forming Zn^2+^(H_2_O)_5_SO_4_^2−^ complexes


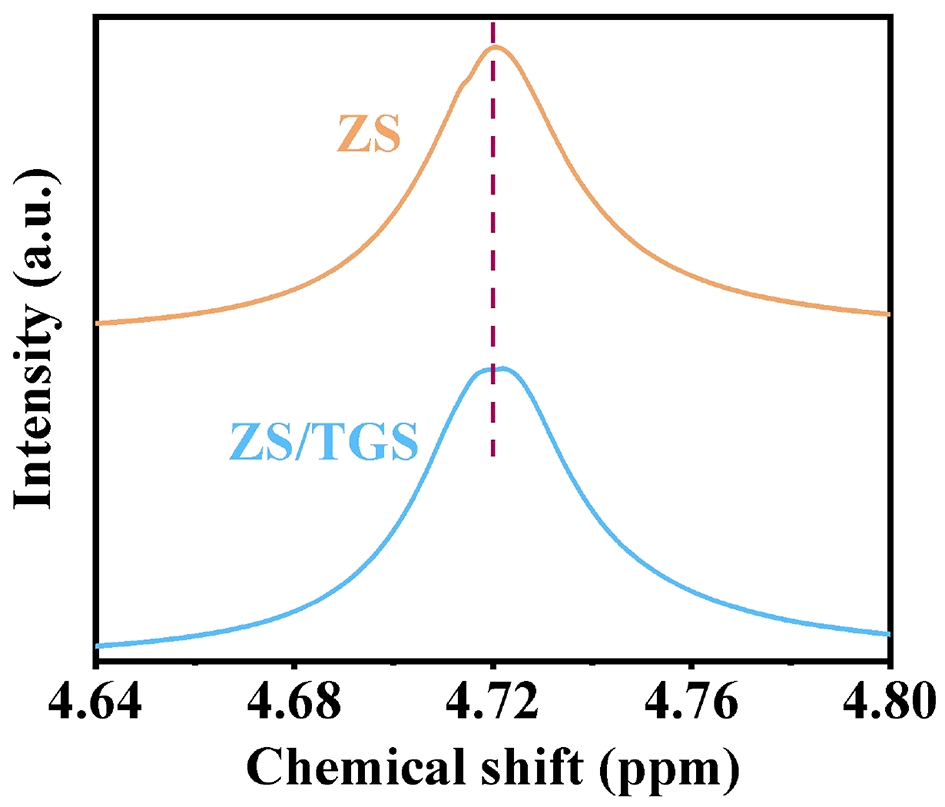


**Fig. S10** ^2^H NMR spectra of different electrolytes


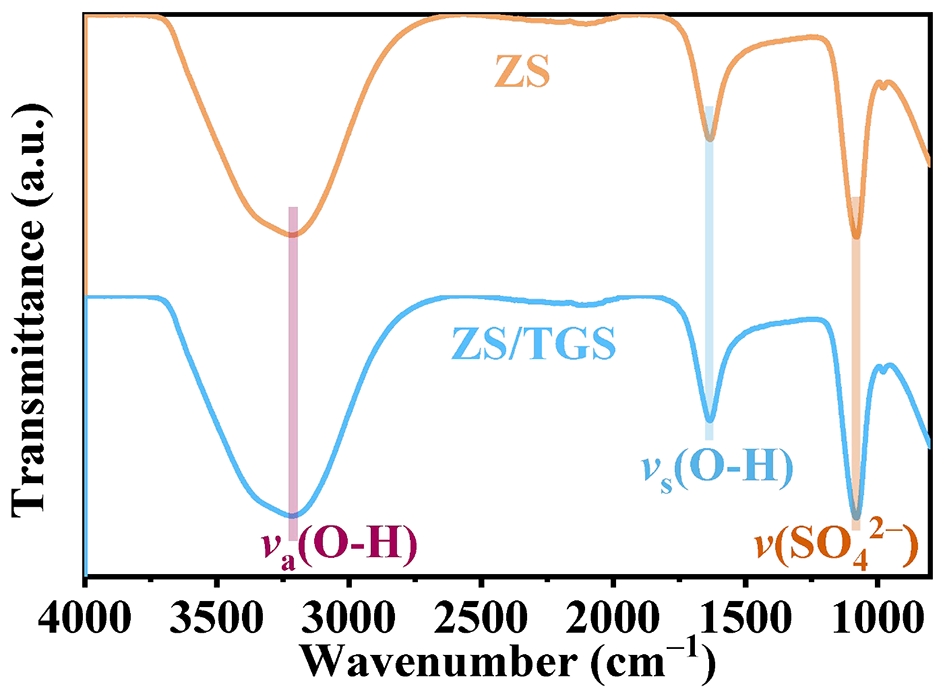


**Fig. S11** Fourier transform infrared (FTIR) spectra of ZS and ZS/TGS electrolytes


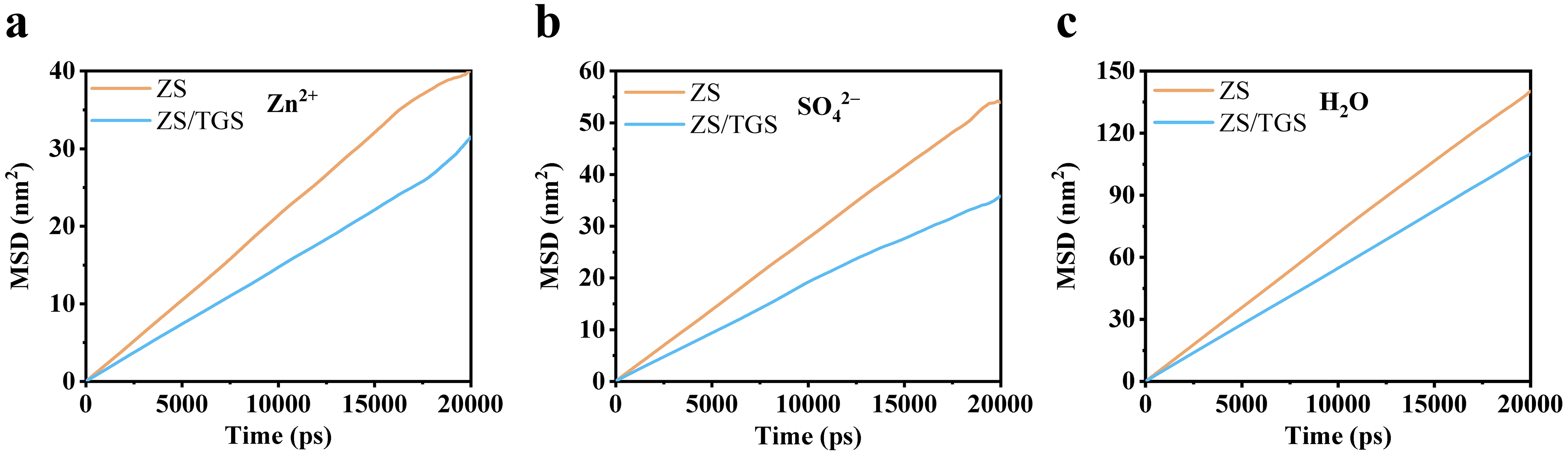


**Fig. S12** Mean squared displacement (MSD) analysis of **a** Zn^2+^ ions, **b** SO_4_^2−^ ions, and **c** H_2_O molecules in ZS and ZS/TGS electrolytes


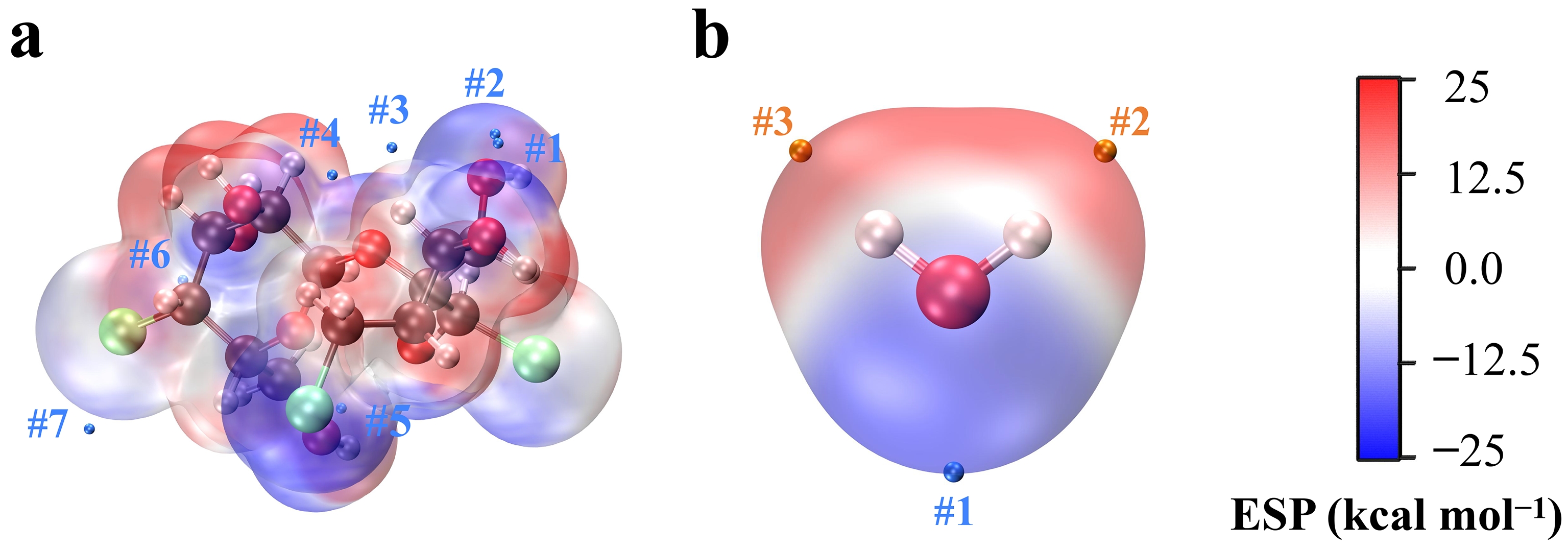


**Fig. S13** Electrostatic potential (ESP) mappings of **a** TGS molecule and **b** H_2_O molecule obtained through QC calculations. The blue points represent surface ESP minima, while the orange points represent ESP maxima. The ESP extreme values corresponding to these points are summarized in Table S1


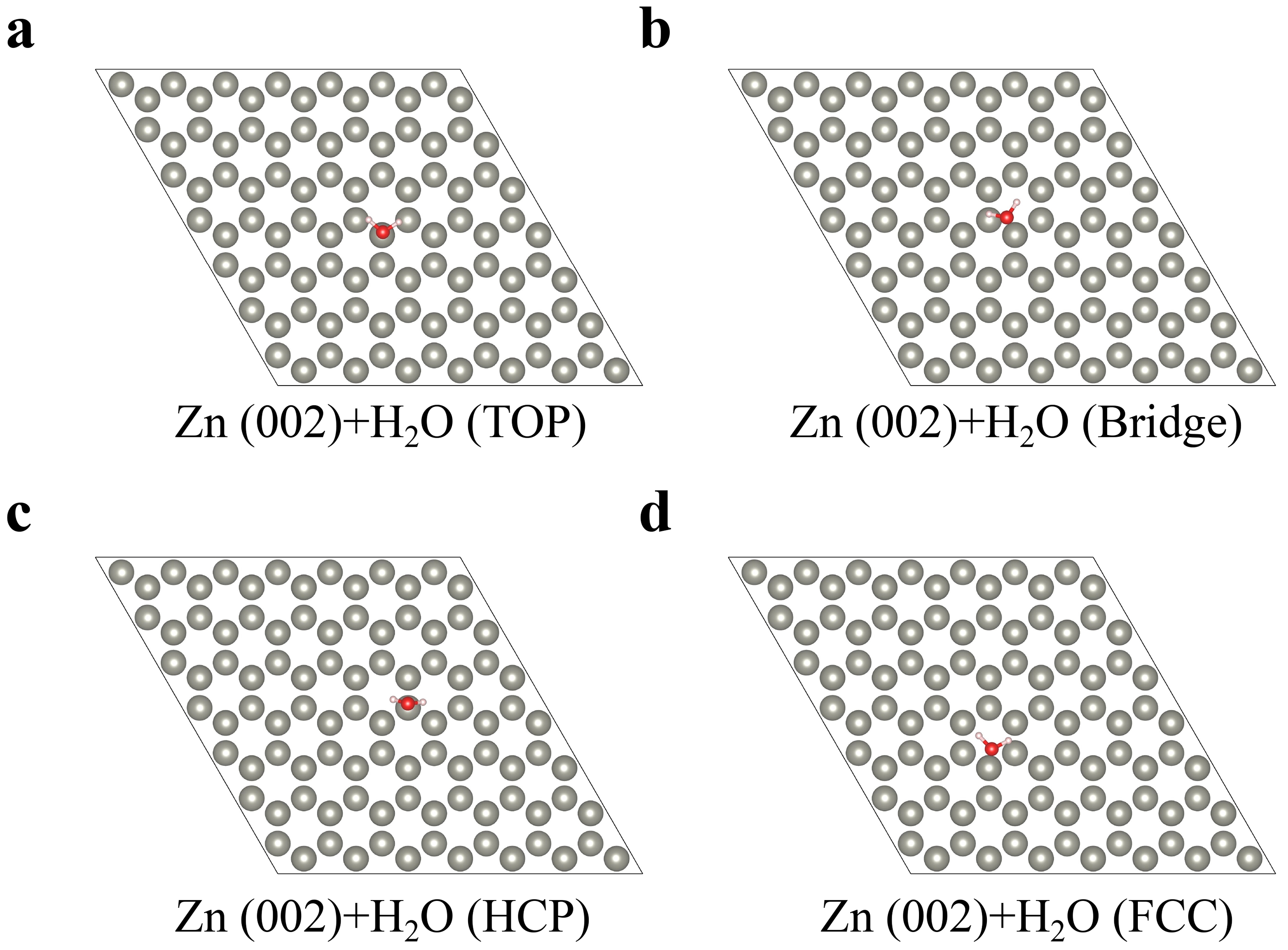


**Fig. S14** Different adsorption configurations of H_2_O molecules on the Zn (002) surface: **a** top site, **b** bridge site, **c** hexagonal close-packed (HCP) site, and **d** face-centered cubic (FCC) site


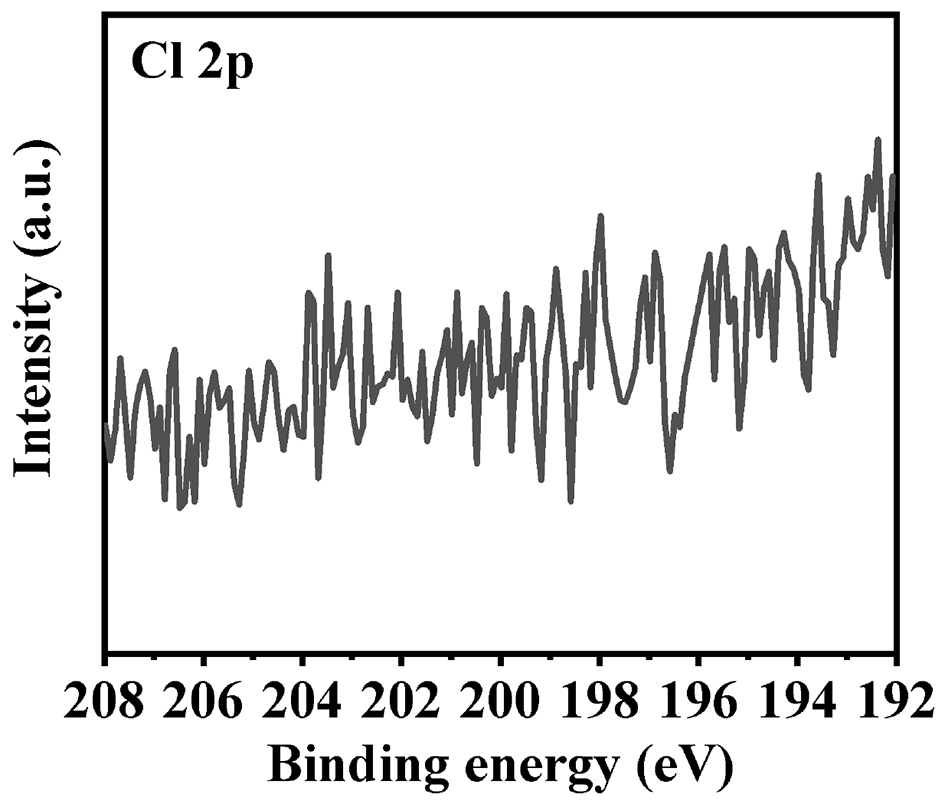


**Fig. S15** X-ray photoelectron spectroscopy (XPS) Cl 2p spectra of Zn foils soaked in the ZS electrolyte for 3 days


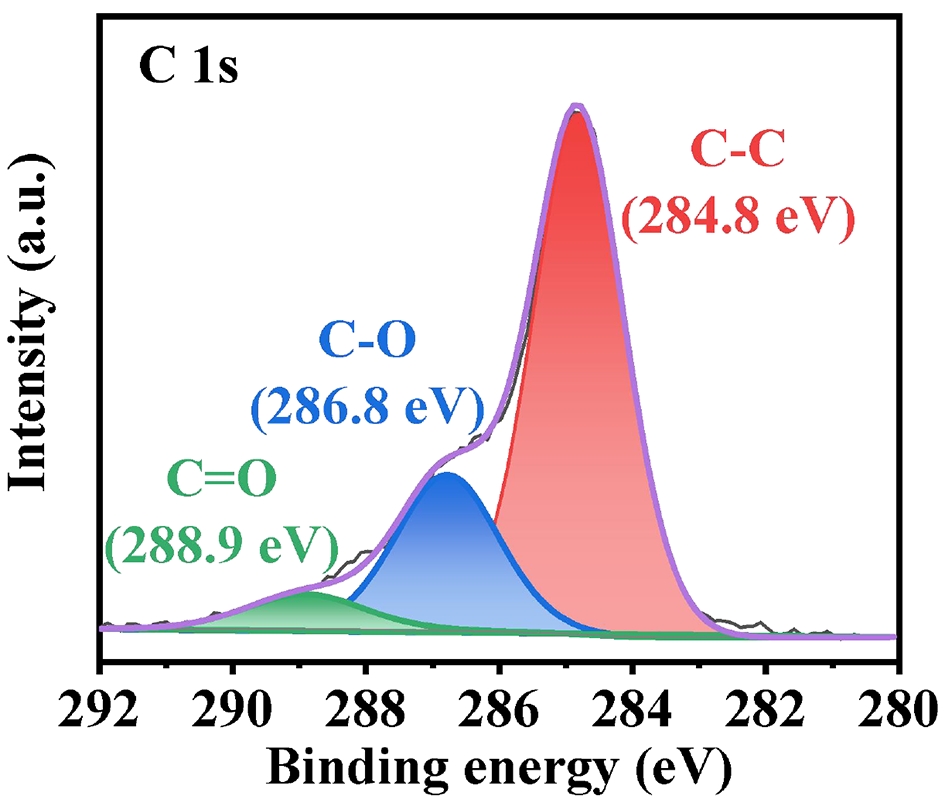


**Fig. S16** XPS C 1s spectrum of Zn foil soaked in ZS/TGS electrolyte for 3 days


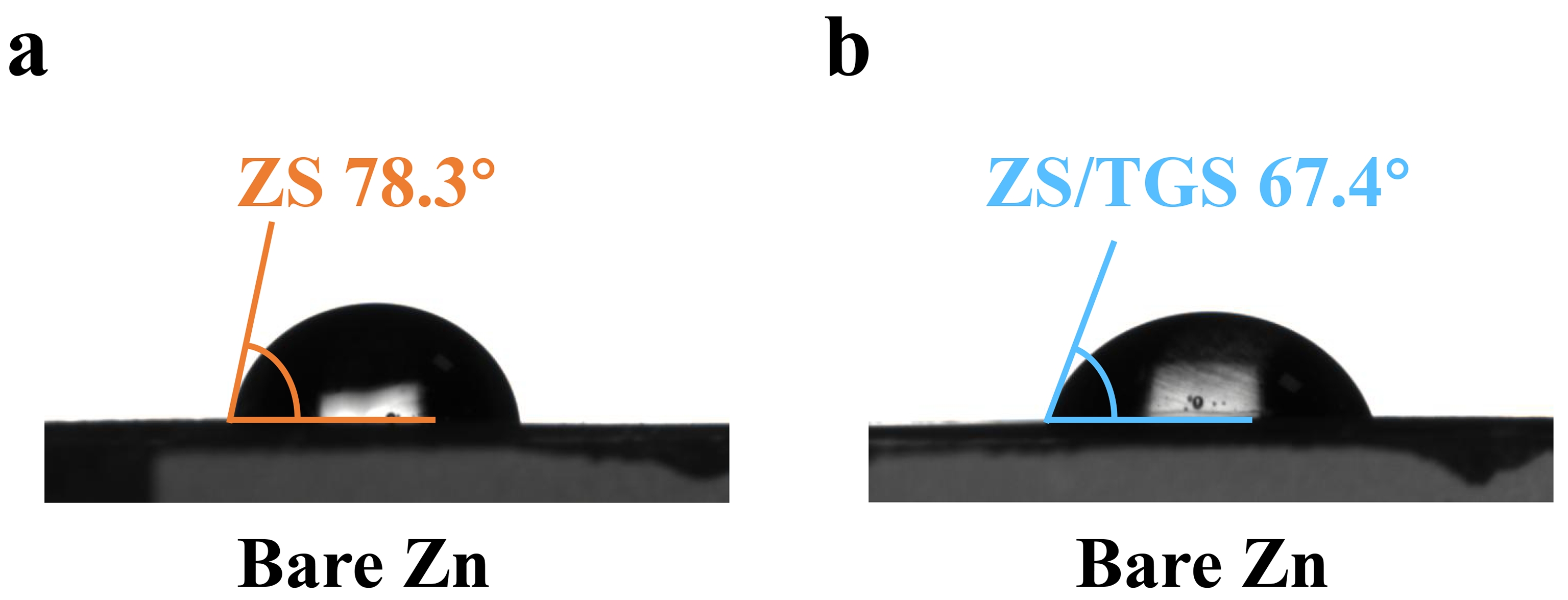


**Fig. S17** Contact angle measurements of **a** ZS electrolyte and **b** ZS/TGS electrolyte on bare Zn foil


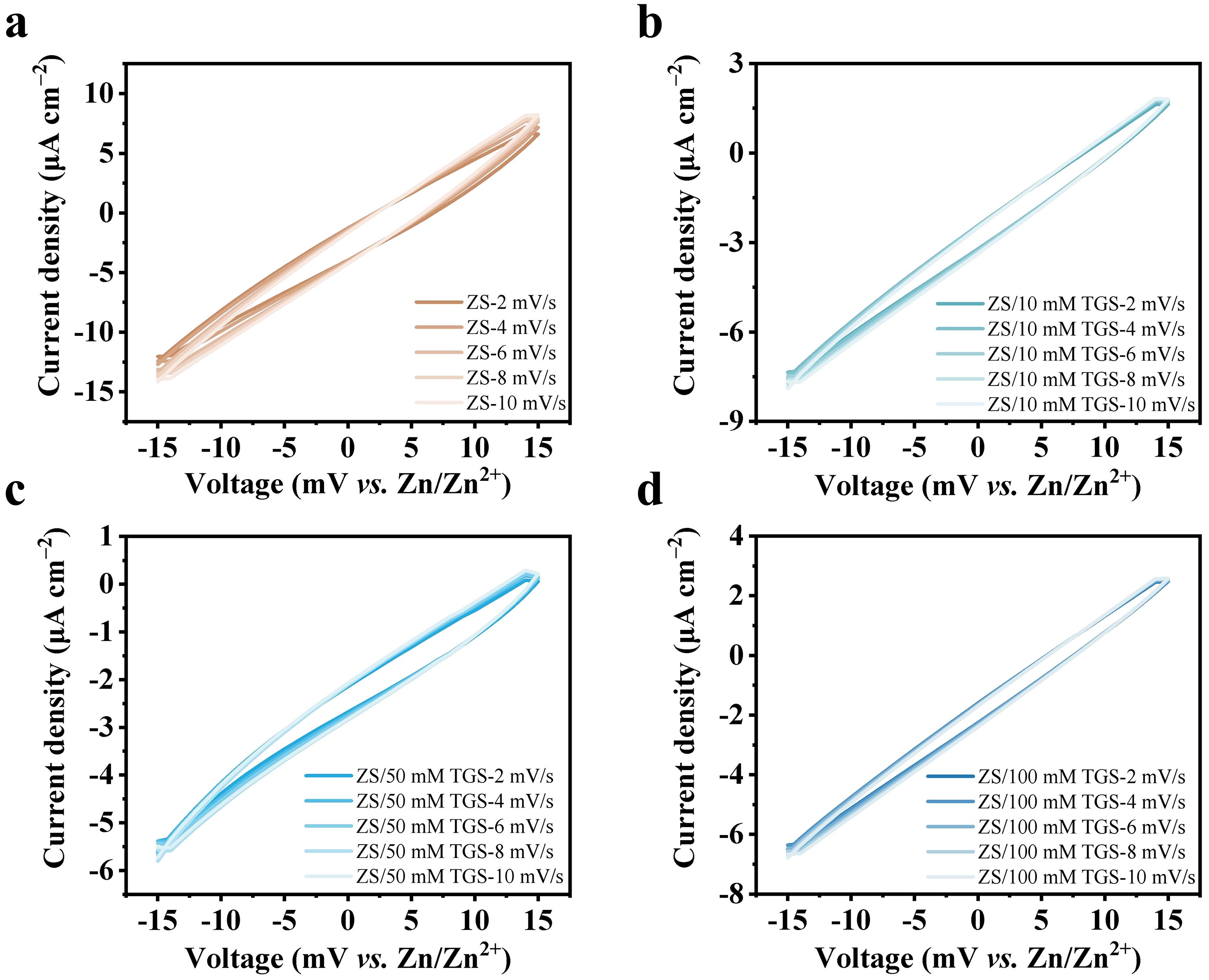


**Fig. S18** Cyclic voltammetry (CV) curves of Zn||Zn symmetric cells at scan rates ranging from 2 to 10 mV s^−1^ in **a** ZS electrolyte, **b** ZS/10 mM TGS electrolyte, **c** ZS/50 mM TGS electrolyte, and **d** ZS/100 mM TGS electrolyte


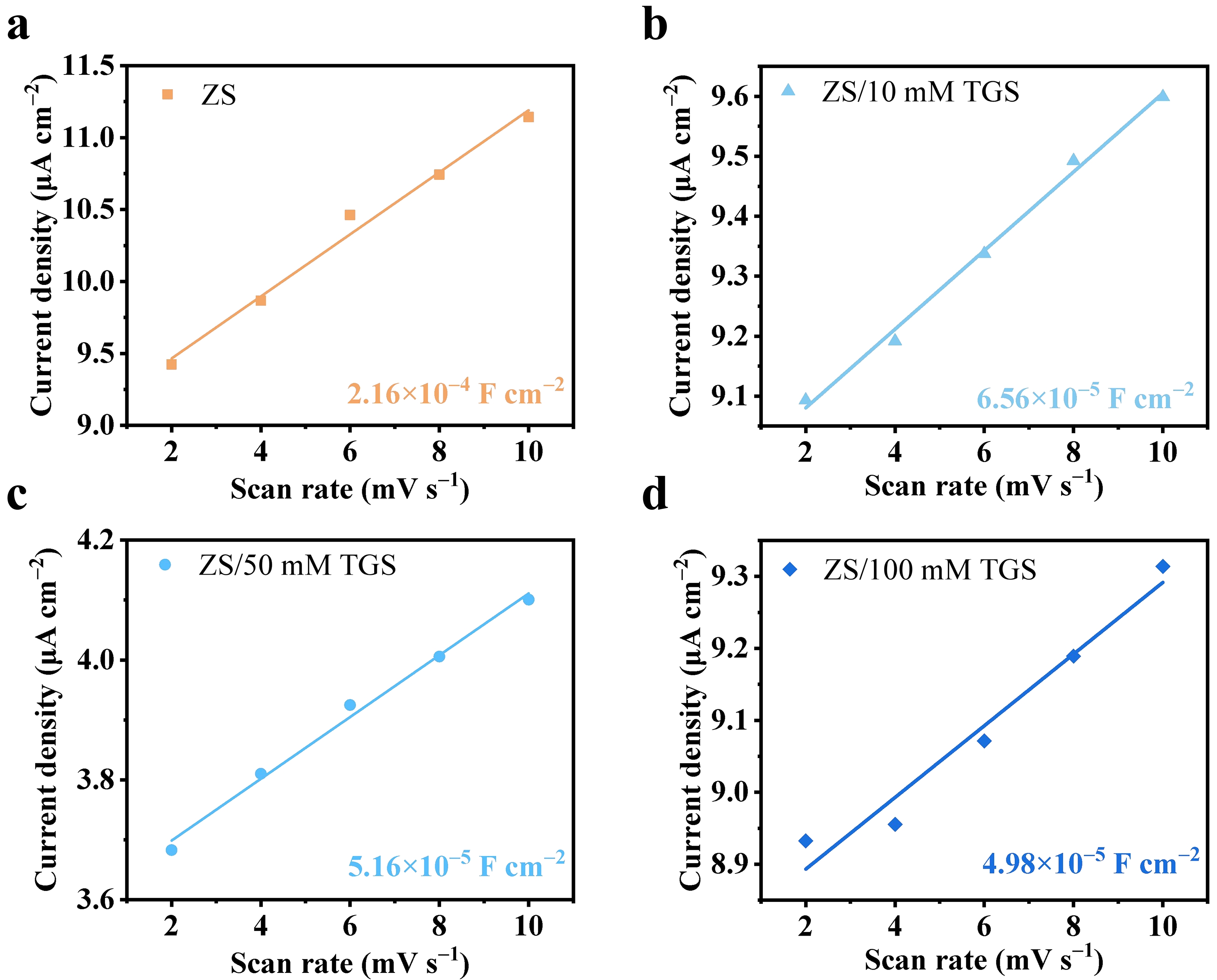


**Fig. S19** The corresponding current density versus scan rate plots in **a** ZS electrolyte, **b** ZS/10 mM TGS electrolyte, **c** ZS/50 mM TGS electrolyte, and **d** ZS/100 mM TGS electrolyte reveal the average differential capacitance of the Zn anode


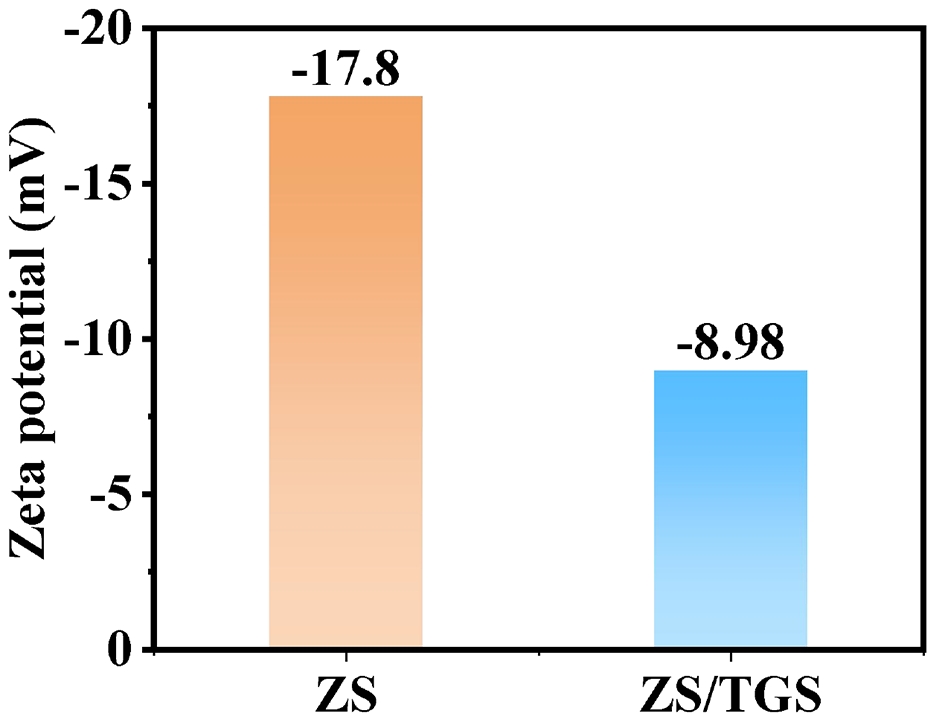


**Fig. S20** Zeta potential of ZS and ZS/TGS electrolytes


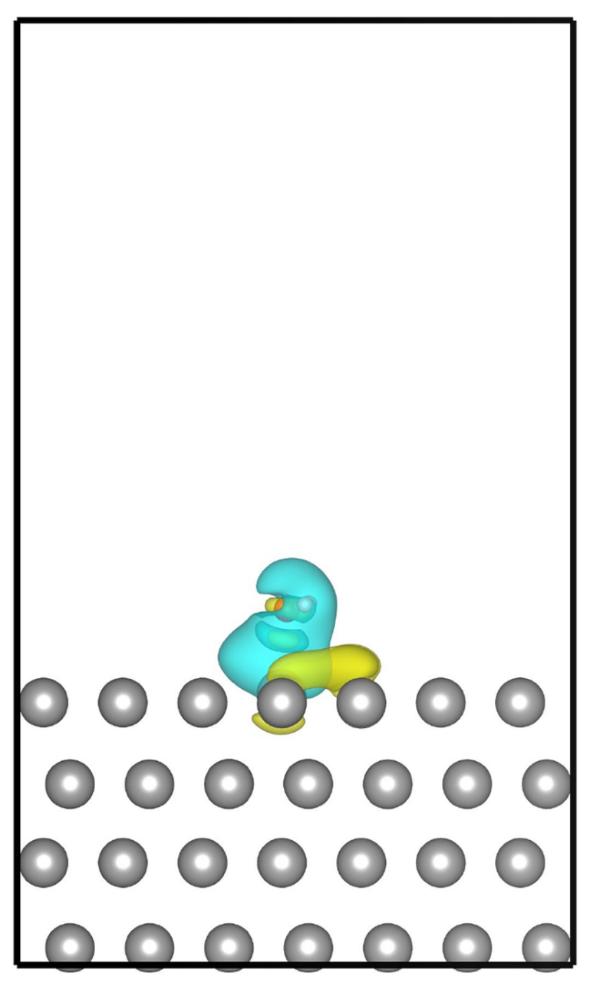


**Fig. S21** Charge density difference (CDD) distribution plot of Zn (002) + H_2_O (iso-value = 5 × 10^−4^ e Bohr^−3^). The yellow and cyan iso-surfaces represent regions of increased and decreased charge density, respectively


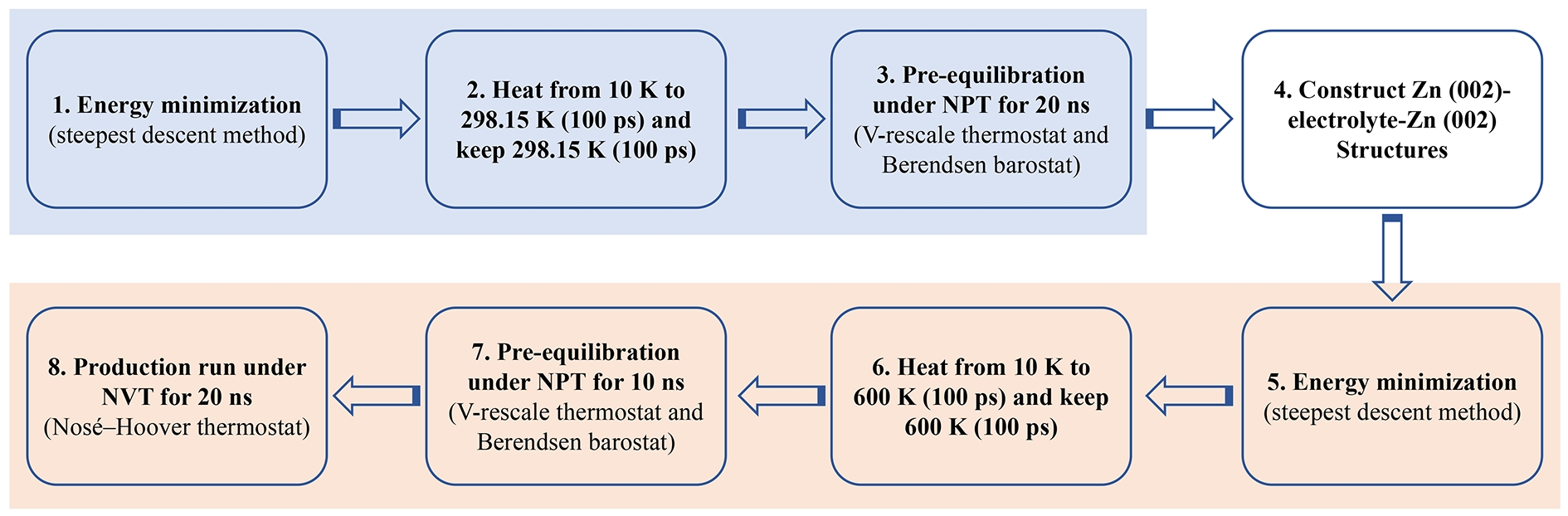


**Fig. S22** Workflow of CMD simulations for the interfacial electrolyte system. The process is divided into two stages: pre-equilibration of the bulk electrolyte (blue background) and simulation of the Zn (002)-electrolyte-Zn (002) interface (orange background)


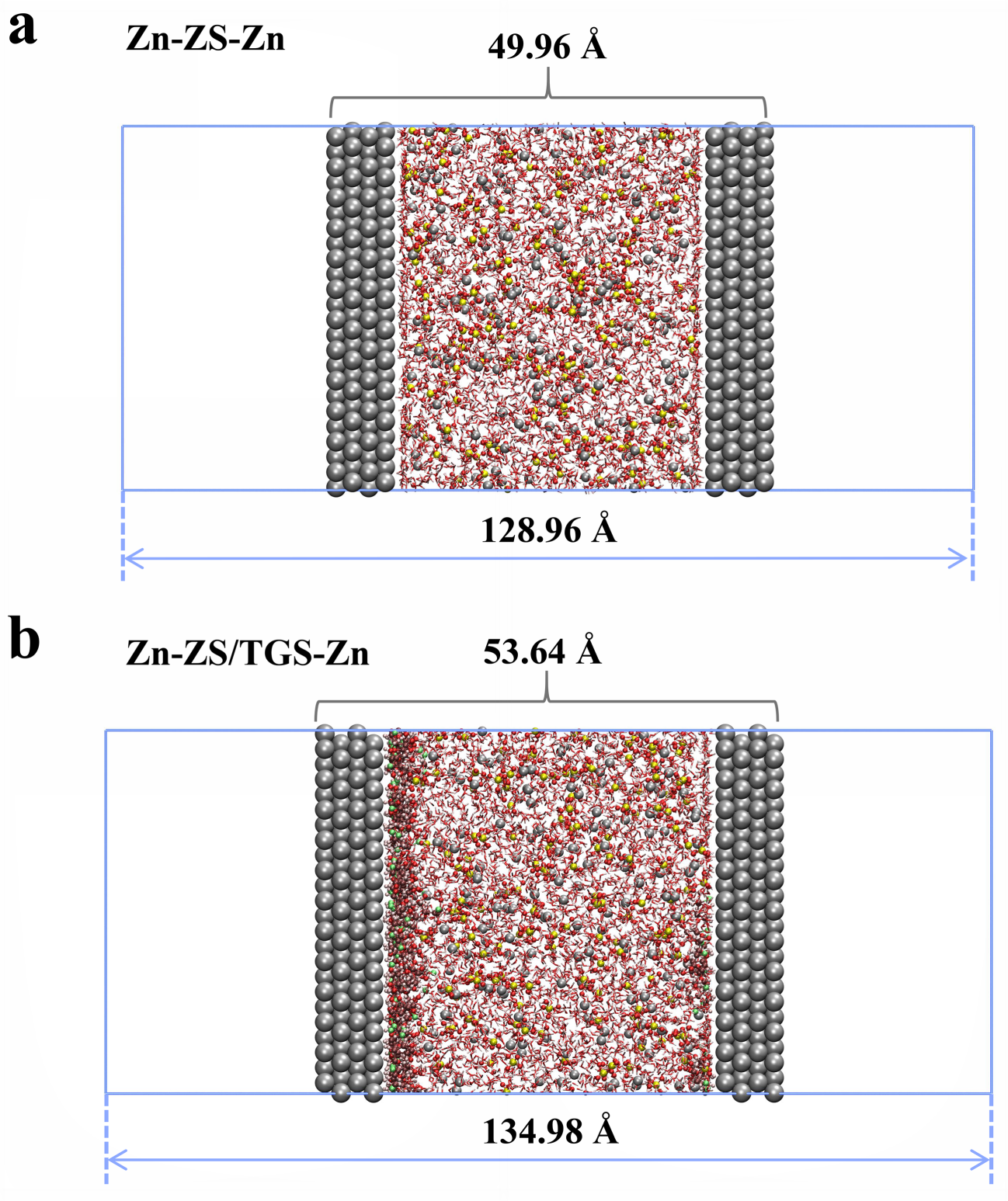


**Fig. S23** Simulated Zn (002)-Electrolytes-Zn (002) structures for **a** Zn-ZS-Zn and **b** Zn-ZS/TGS-Zn systems


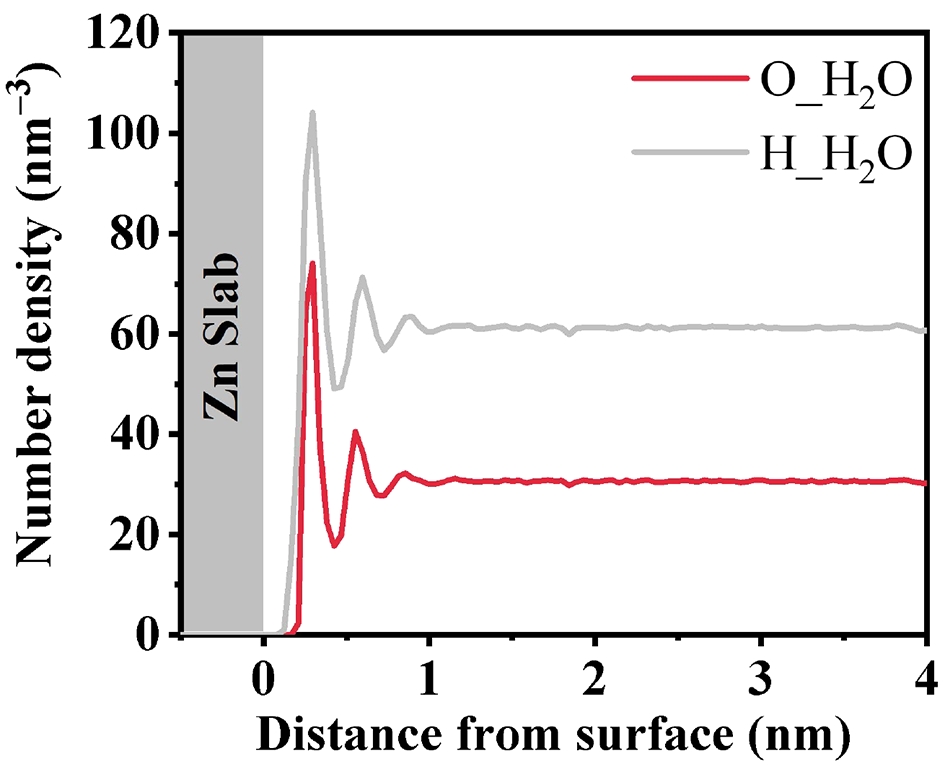


**Fig. S24** Number density distributions of specific elements in H_2_O (O and H) near the Zn (002) slab


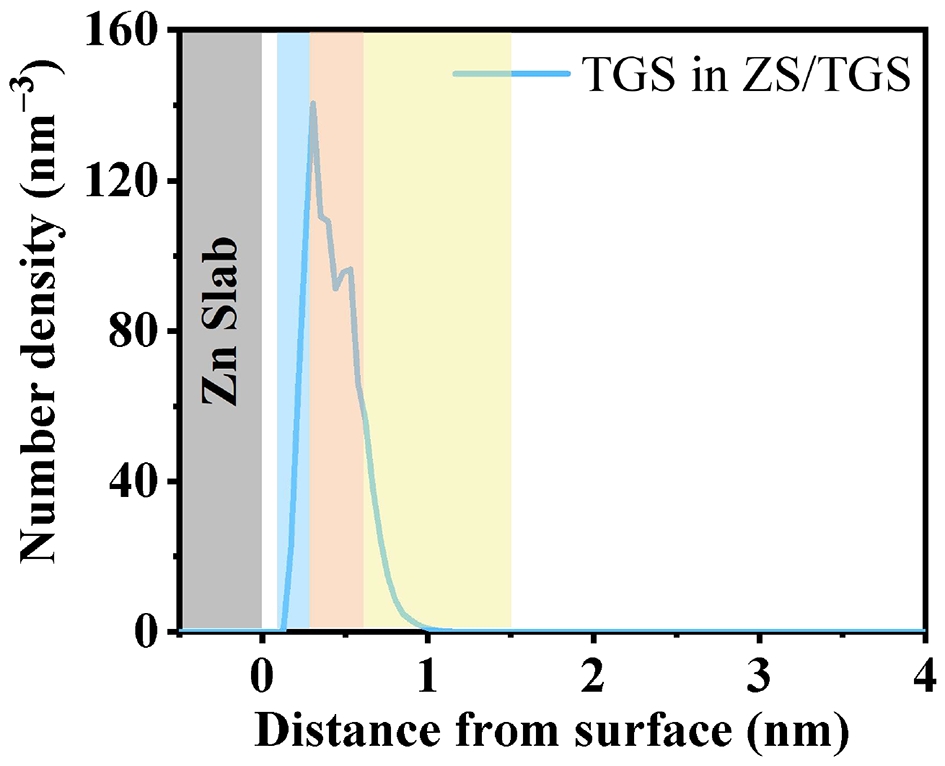


**Fig. S25** The number density of TGS molecules in the Zn-ZS/TGS system as a function of distance from the Zn slab surface. The shaded regions represent the interfacial zones: the IHP (blue background), the OHP (orange background), and the DL (yellow background)


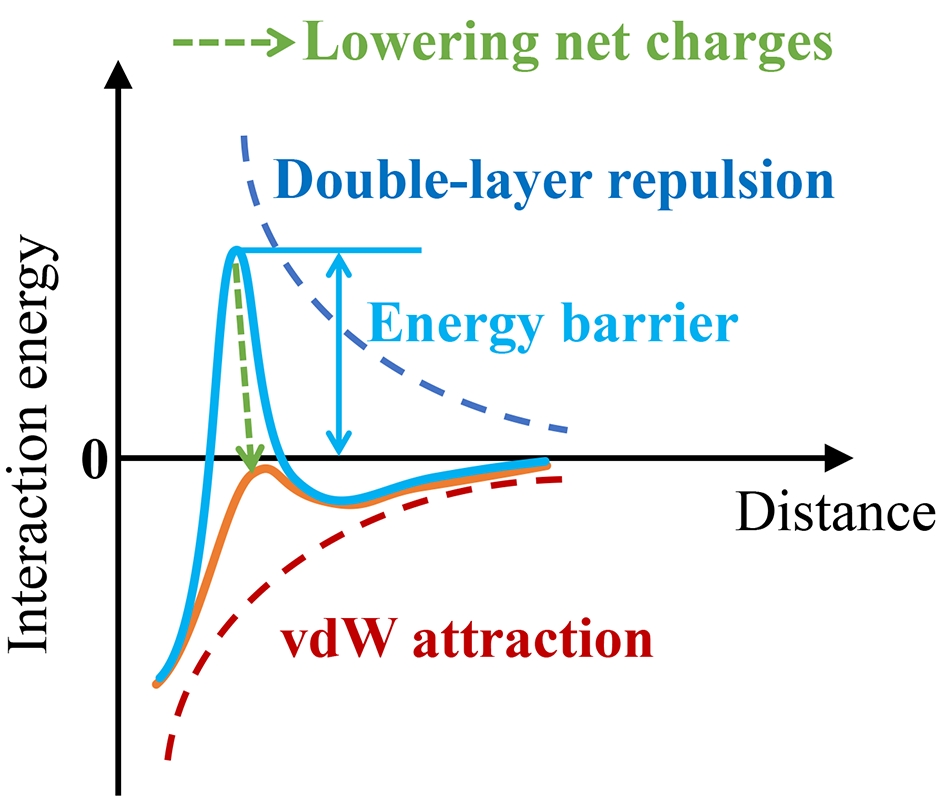


**Fig. S26** Schematic diagrams of the interaction energy and distance curve according to the Derjaguin-Landau-Verwey-Overbeek (DLVO) theory [S6, S7]


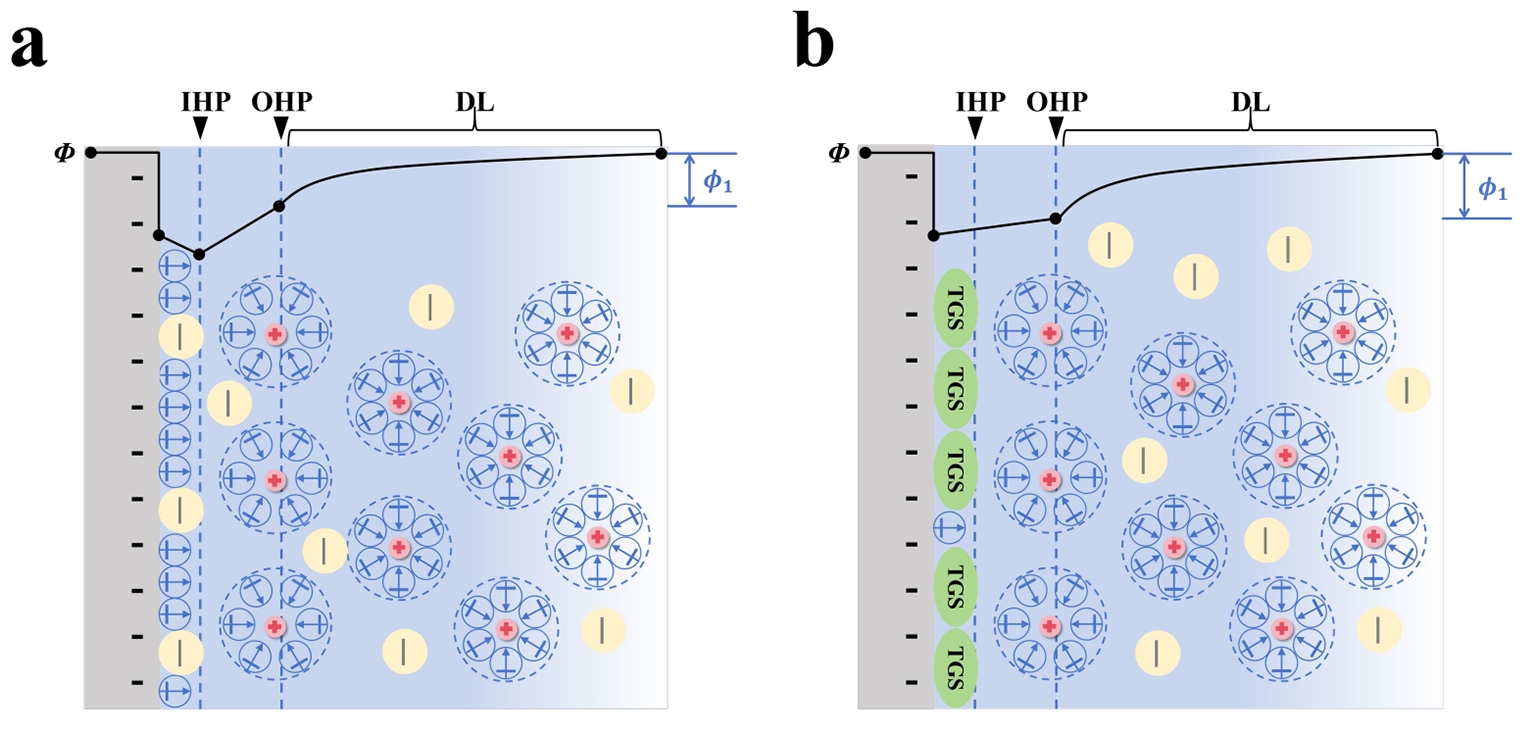


**Fig. S27** Schematic diagrams of the EDL structures and corresponding potential drops as a function of their position in **a** ZS and **b** ZS/TGS electrolytes


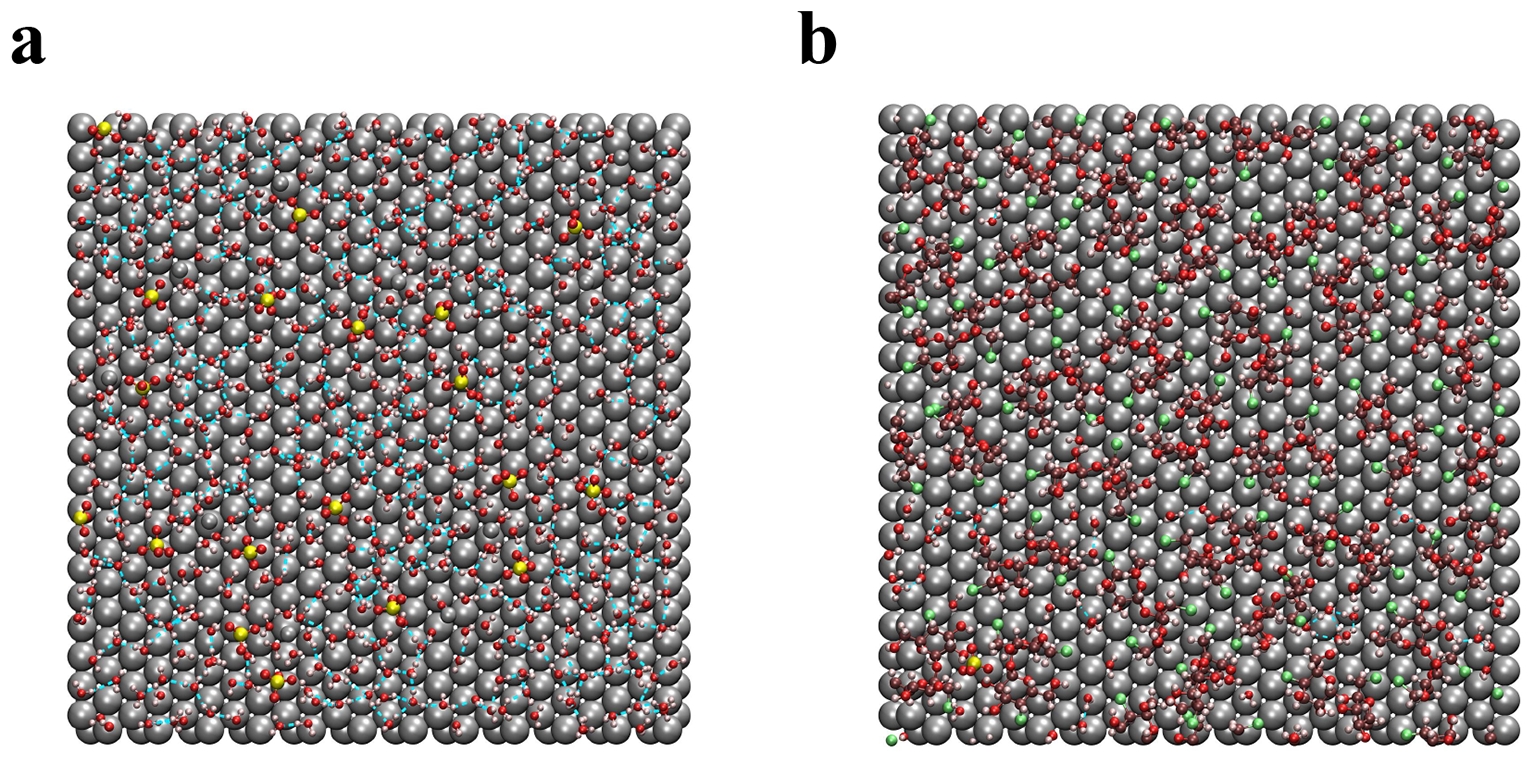


**Fig. S28** Vertical view of the Zn (002)-electrolyte interface in **a** Zn-ZS and **b** Zn-ZS/TGS systems. The blue dotted lines represent hydrogen bonds (H-bonds). In the Zn (002)-ZS system, the Zn metal surface exhibits numerous continuous H-bonds among water molecules, forming a stable hydration layer. In contrast, in the Zn (002)-ZS/TGS system, the addition of TGS effectively displaces surface water molecules, breaking the continuous hydrogen bond network


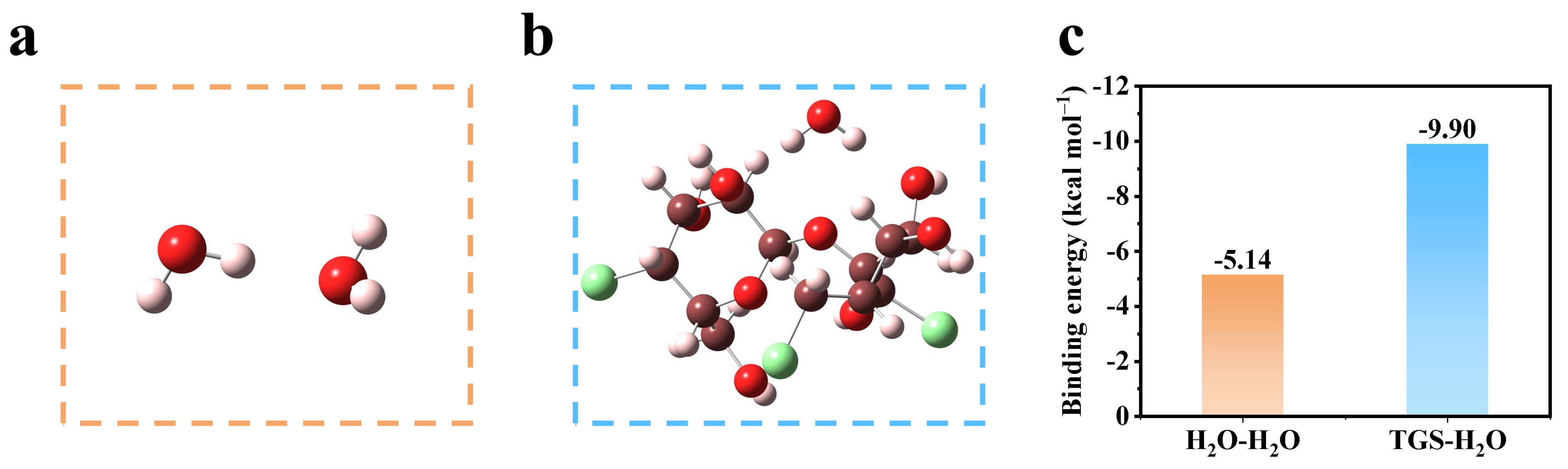


**Fig. S29** **a** H_2_O-H_2_O interaction structure, **b** TGS-H_2_O interaction structure, and **c** binding energies comparison from QC calculations


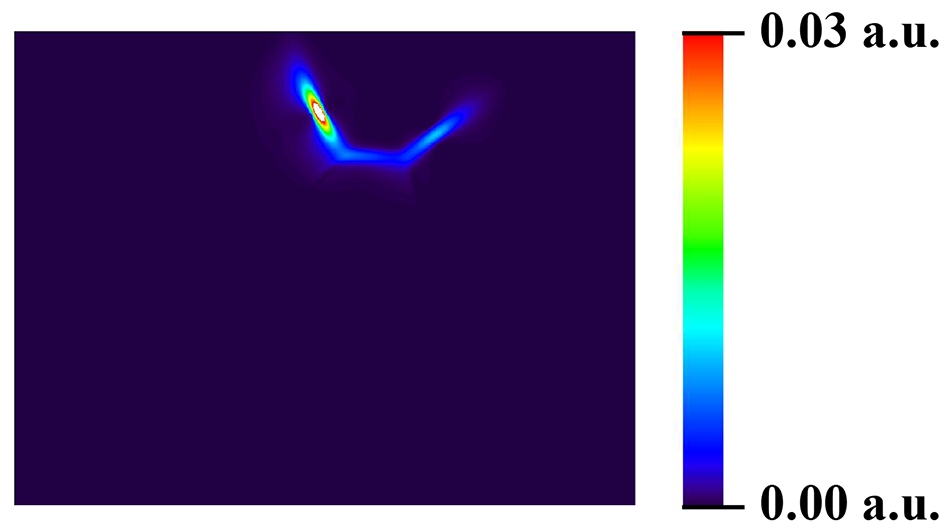


**Fig. S30** 2D plane map of the TGS-H_2_O interaction, highlighting van der Waals (vdW) and weak H-bond interactions using the independent gradient model based on Hirshfeld partition (IGMH) analysis


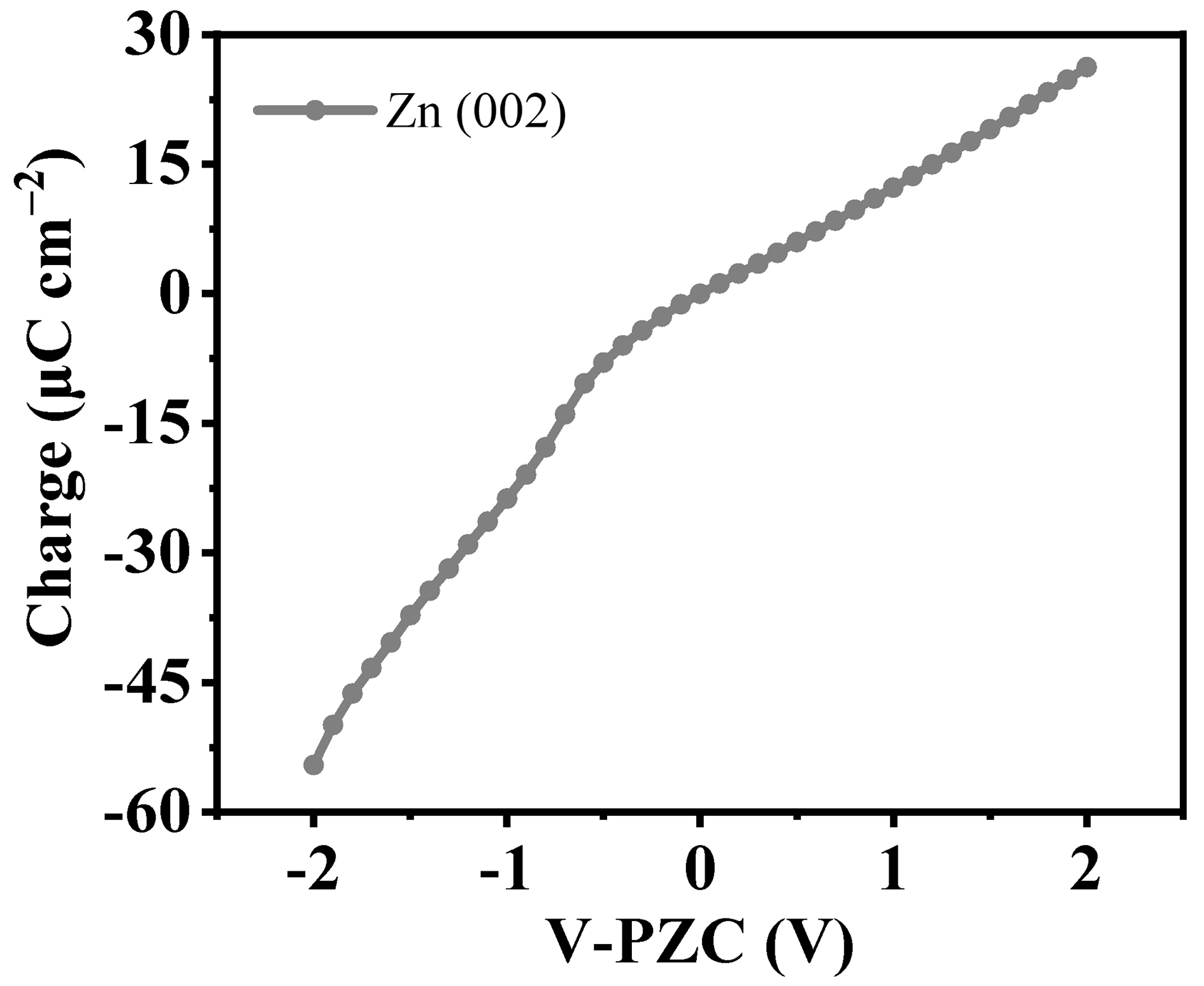


**Fig. S31** Surface charge as a function of potential for the Zn (002) surface. The curve illustrates the variation in surface charge density with potential relative to the potential of zero charge (PZC)


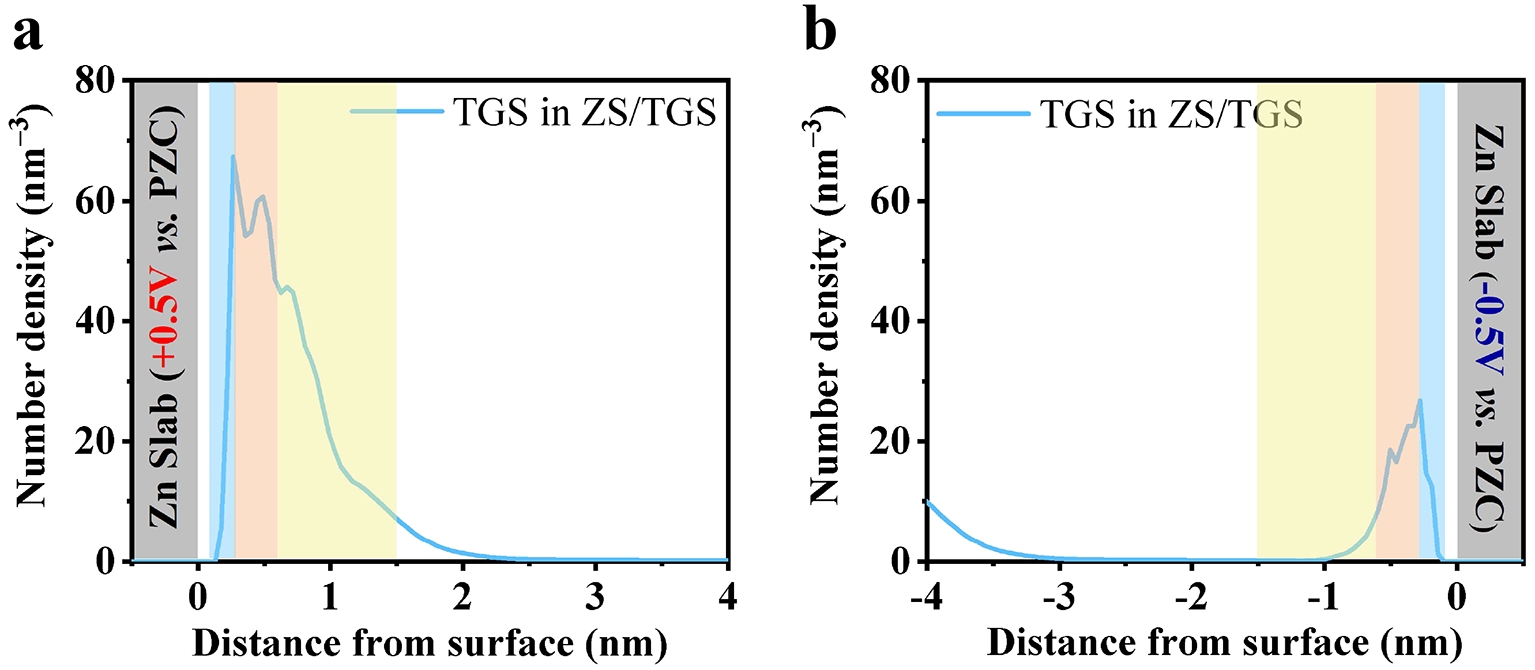


**Fig. S32** The number density of TGS molecules in the Zn (002)-ZS/TGS-Zn (002) system as a function of distance from the Zn slab under different applied potentials: **a** +0.5 V *vs*. PZC and **b** −0.5 V *vs*. PZC


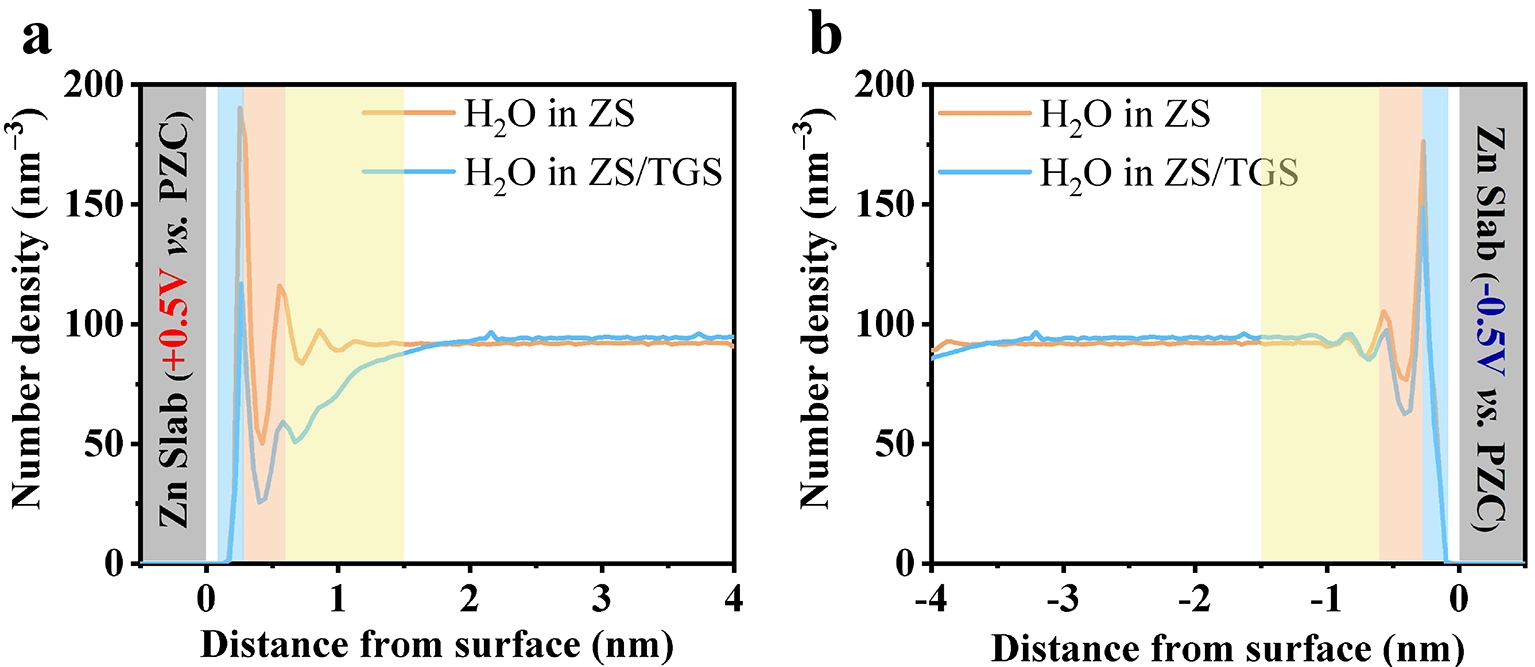


**Fig. S33** The number density of H_2_O molecules as a function of distance from the Zn slab under different applied potentials: **a** +0.5 V *vs*. PZC and **b** −0.5 V *vs*. PZC


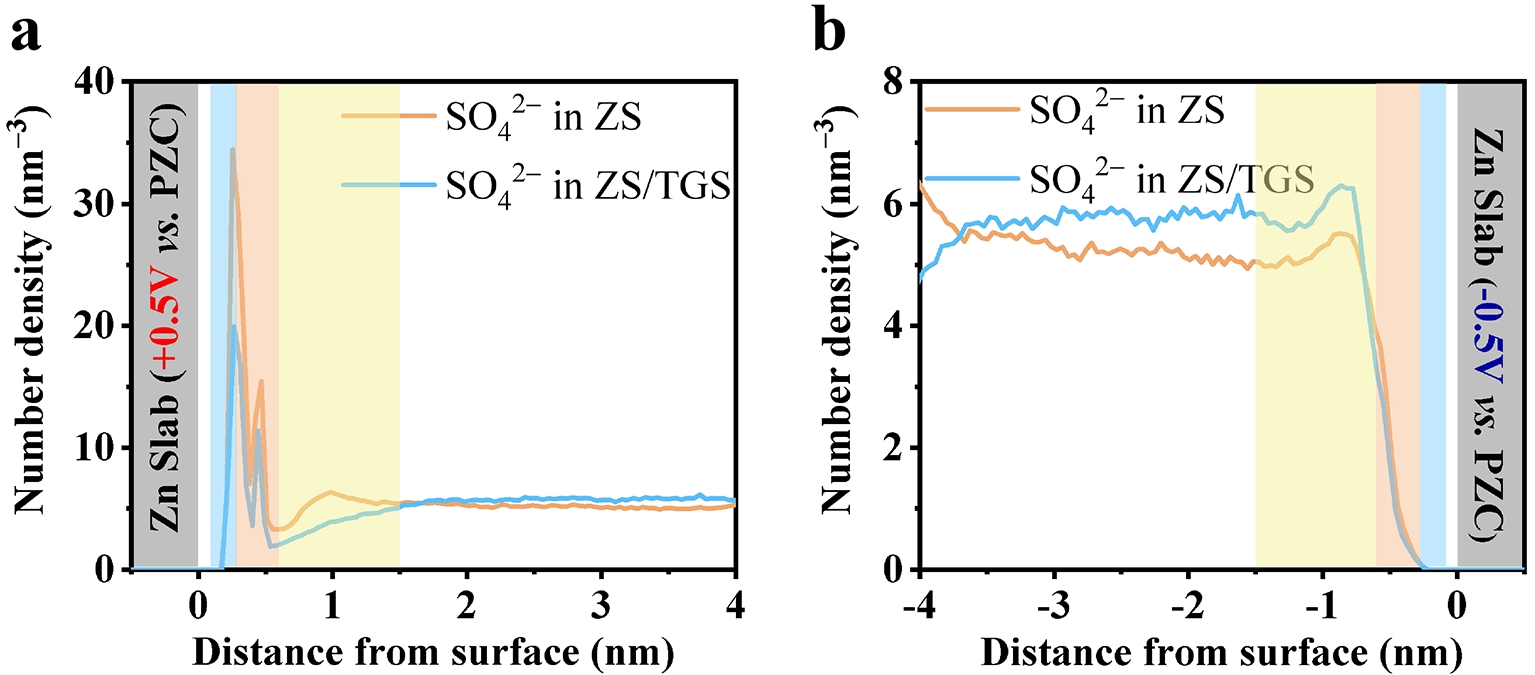


**Fig. S34** The number density of SO_4_^2−^ molecules as a function of distance from the Zn slab under different applied potentials: **a** +0.5 V *vs*. PZC and **b** −0.5 V *vs*. PZC


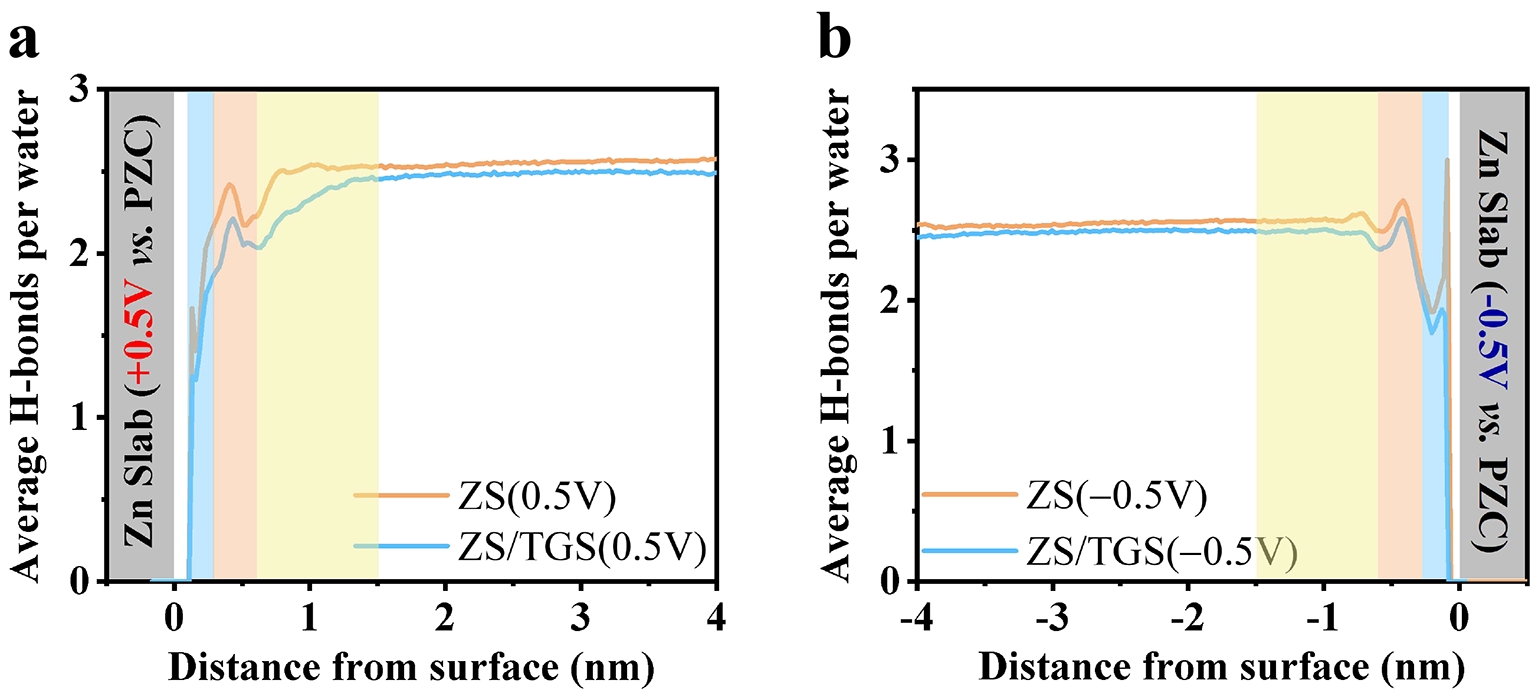


**Fig. S35** Average H-bond number per water molecule as a function of distance from the Zn slab for **a** +0.5 V *vs*. PZC and **b** −0.5 V *vs*. PZC


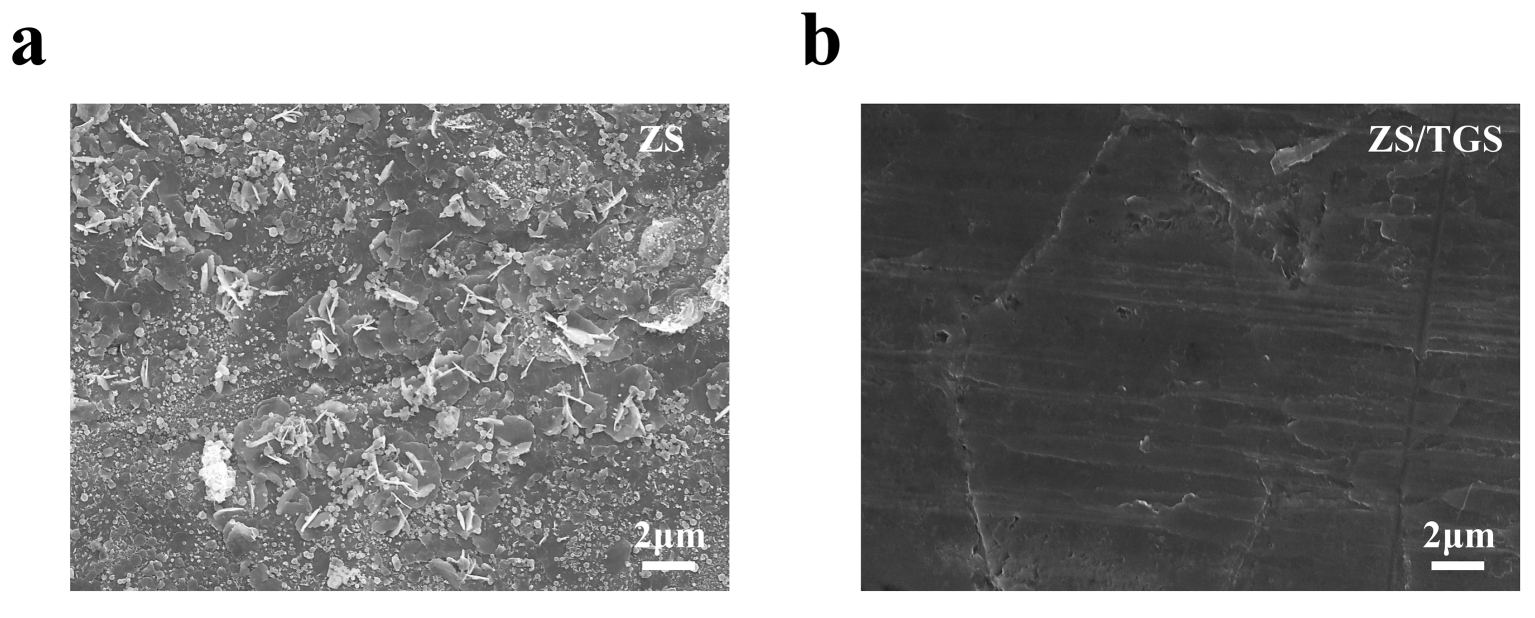


**Fig. S36** Scanning electron microscopy (SEM) images of Zn foils immersed in **a** ZS and **b** ZS/TGS electrolytes for 3 days


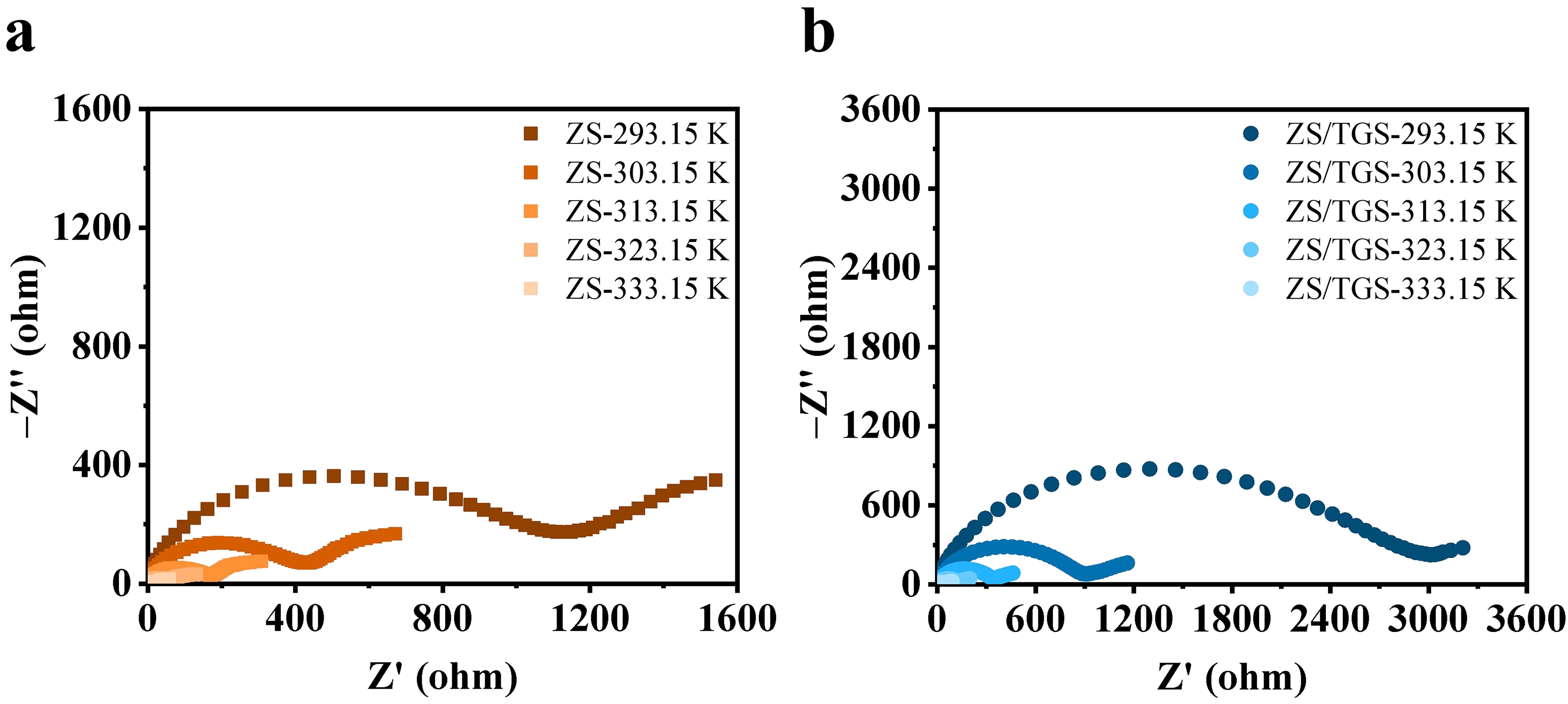


**Fig. S37** EIS spectra of Zn||Zn symmetric cells in **a** ZS electrolyte and **b** ZS/TGS electrolyte at different temperatures


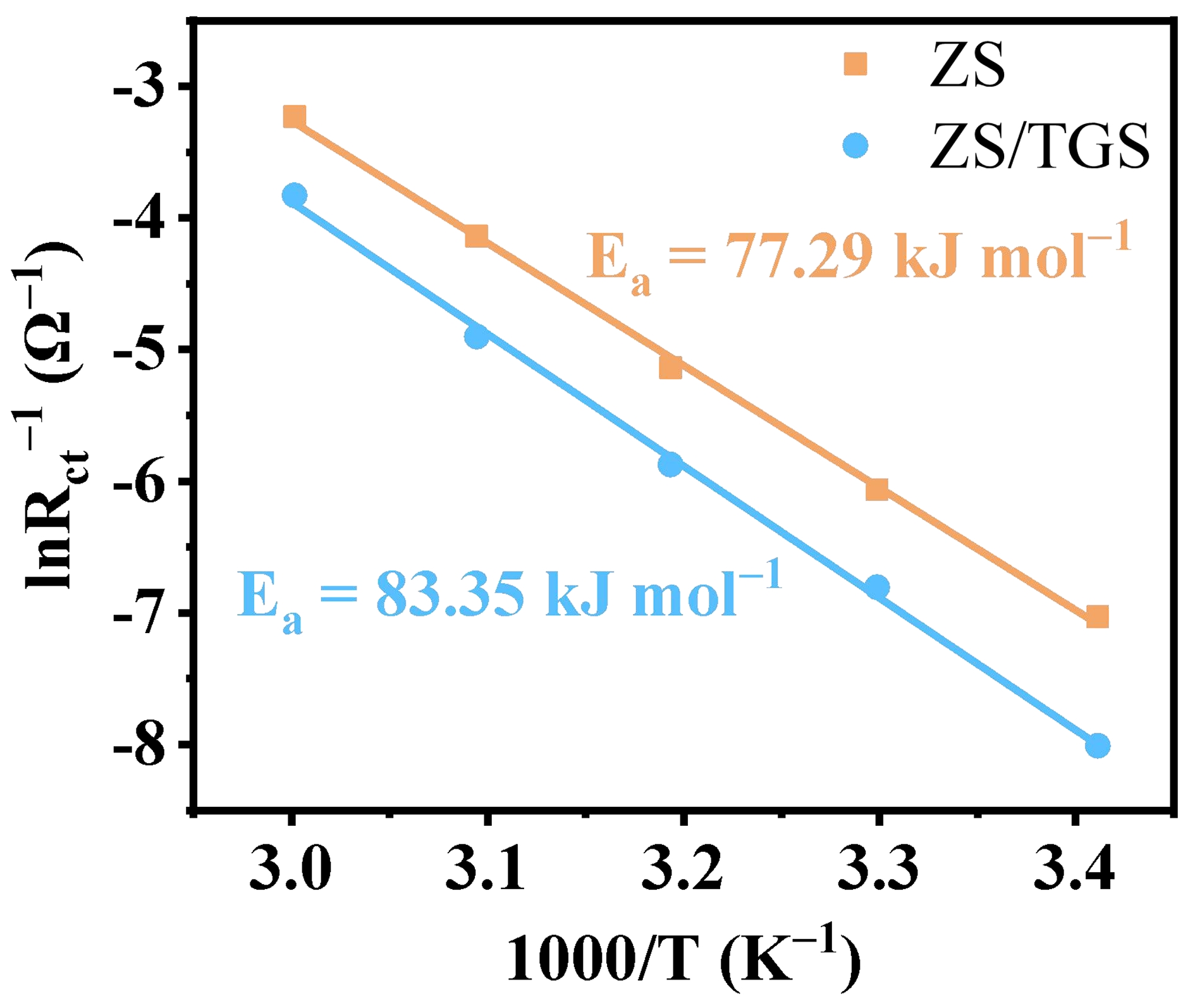


**Fig. S38** Activation energy calculation for ZS electrolyte and ZS/TGS electrolyte using the Arrhenius equations


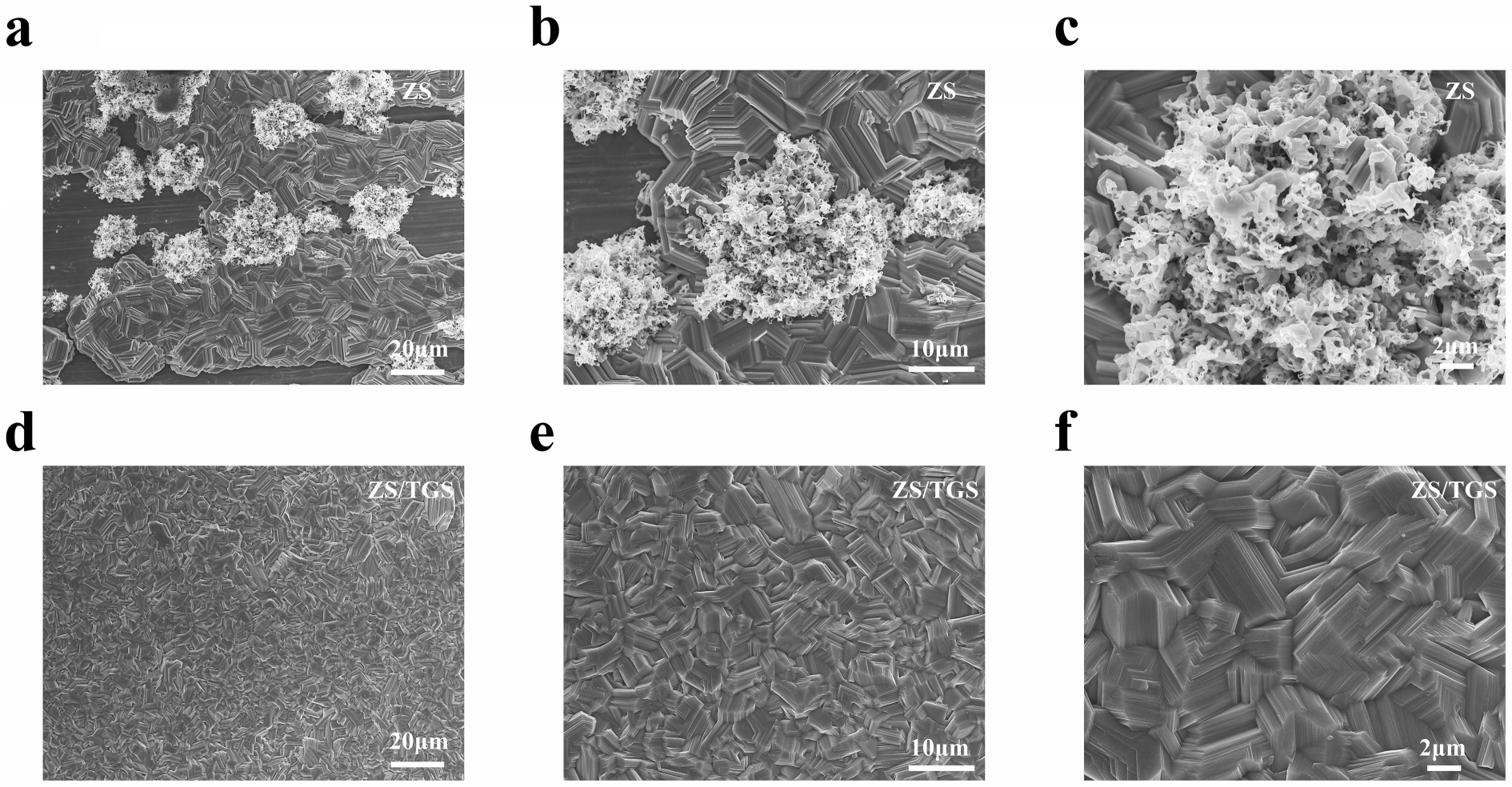


**Fig. S39** SEM images of Zn electrodeposited on Zn substrates at a current density of 5 mA cm^−2^ and an areal capacity of 2.5 mAh cm^−2^ in **a-c** ZS and **d-f** ZS/TGS electrolytes


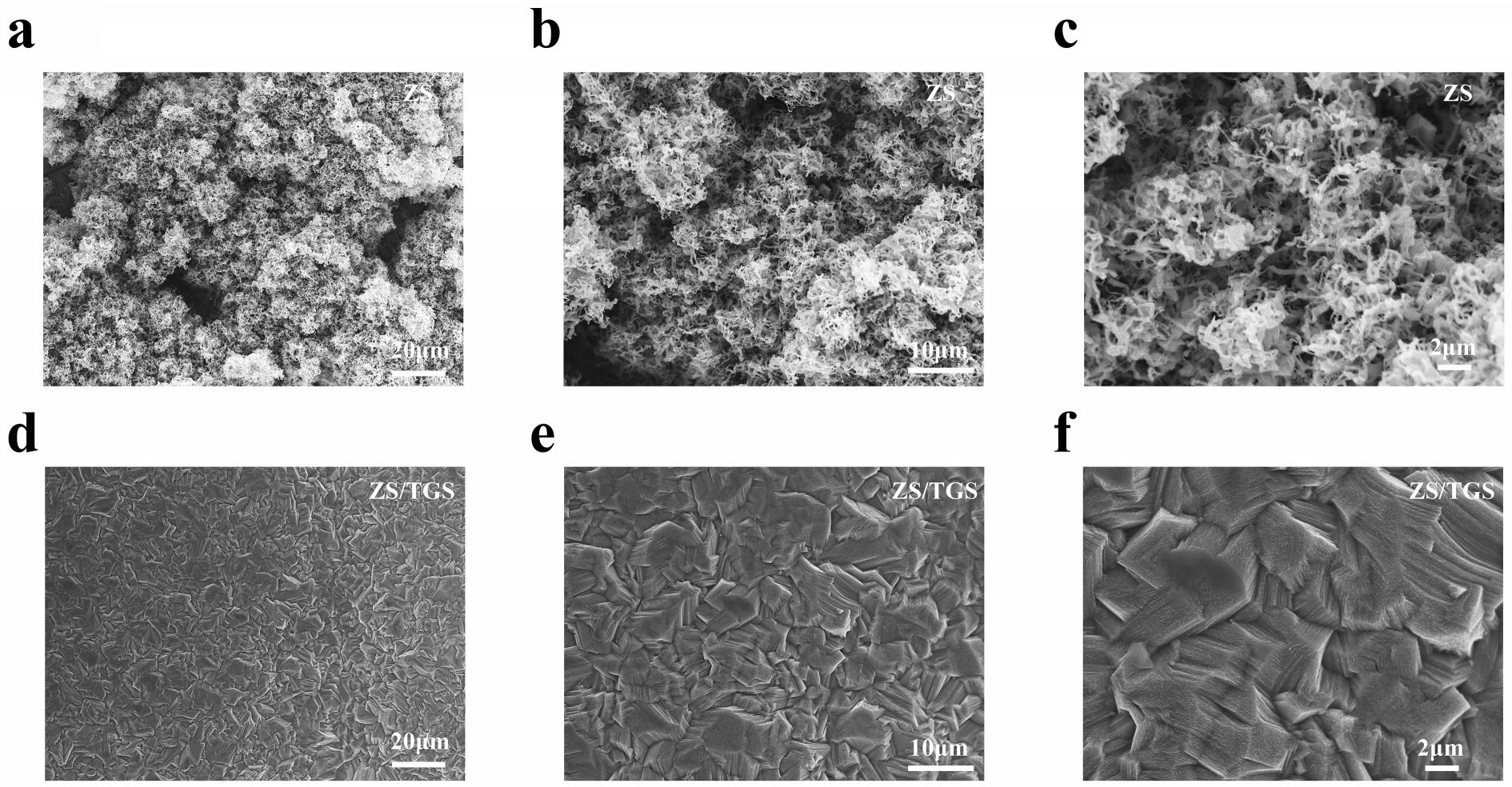


**Fig. S40** SEM images of Zn electrodeposited on Zn substrates at a current density of 5 mA cm^−2^ and an areal capacity of 5 mAh cm^−2^ in **a-c** ZS and **d-f** ZS/TGS electrolytes


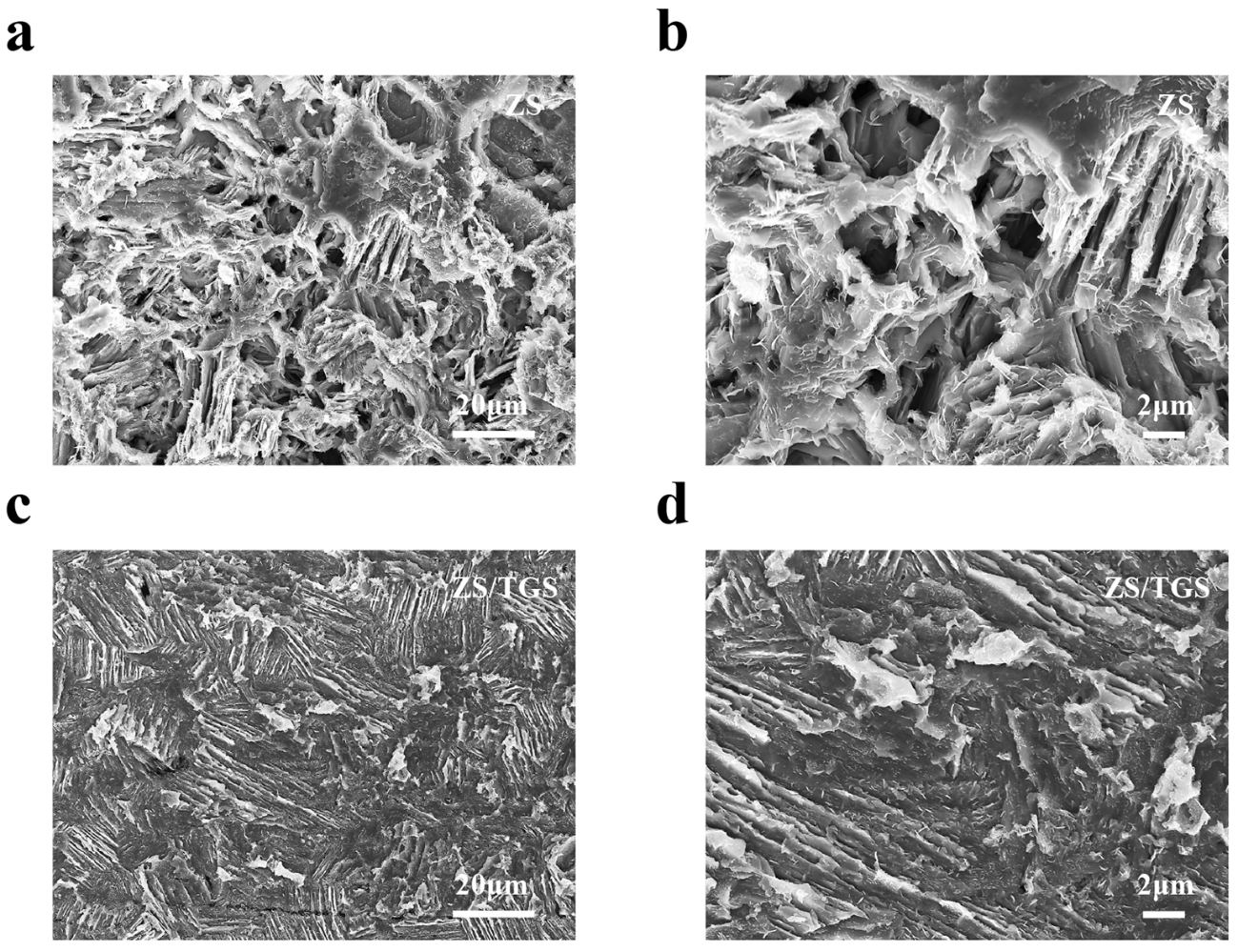


**Fig. S41** SEM images of cycled Zn foils after 10 cycles at a current density of 4 mA cm^−2^ and an areal capacity of 2 mAh cm^−2^ in **a, b** ZS and **c, d** ZS/TGS electrolytes

**
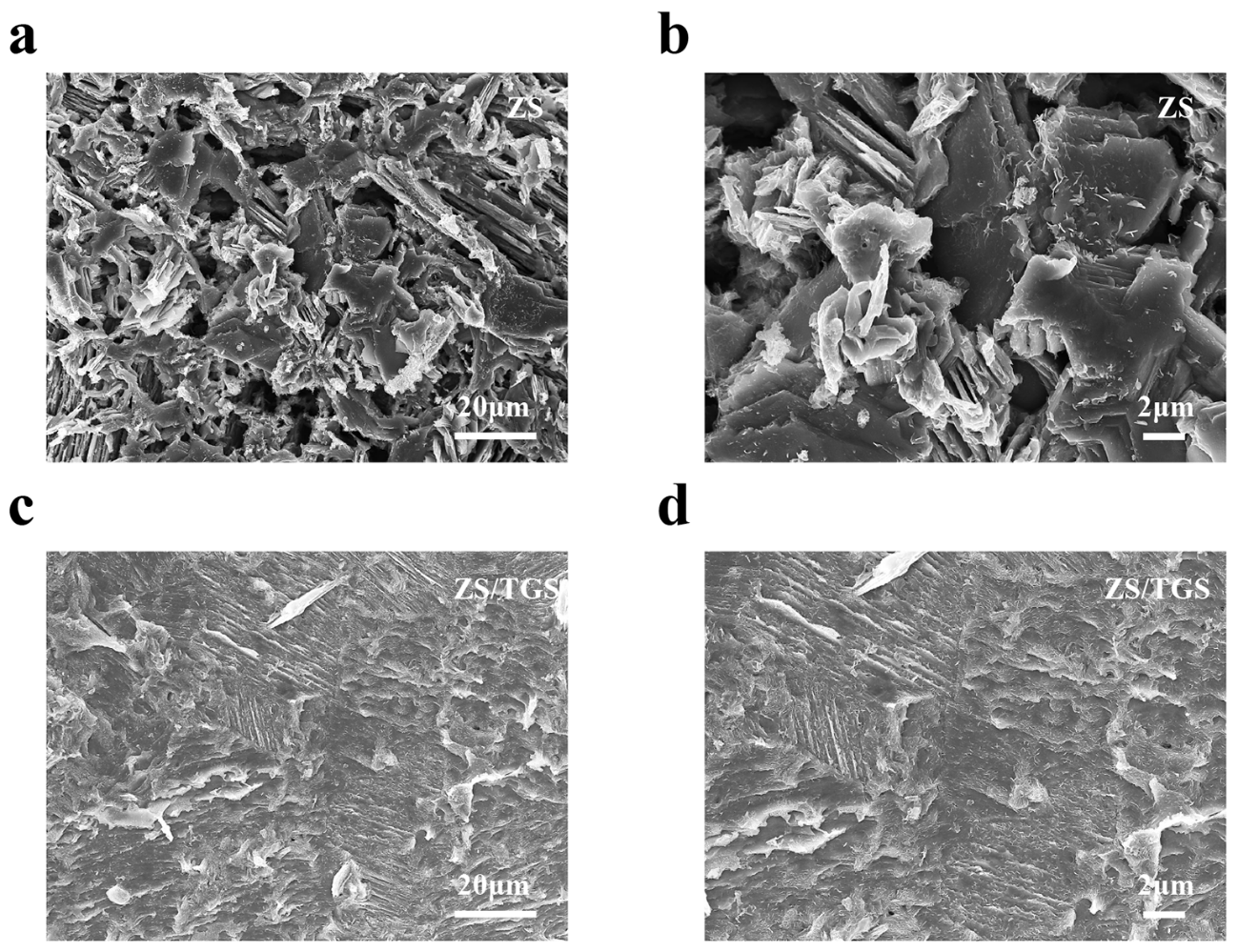
**

**Fig. S42** SEM images of cycled Zn foils after 20 cycles at a current density of 4 mA cm^−2^ and an areal capacity of 2 mAh cm^−2^ in **a, b** ZS and **c, d** ZS/TGS electrolytes

**
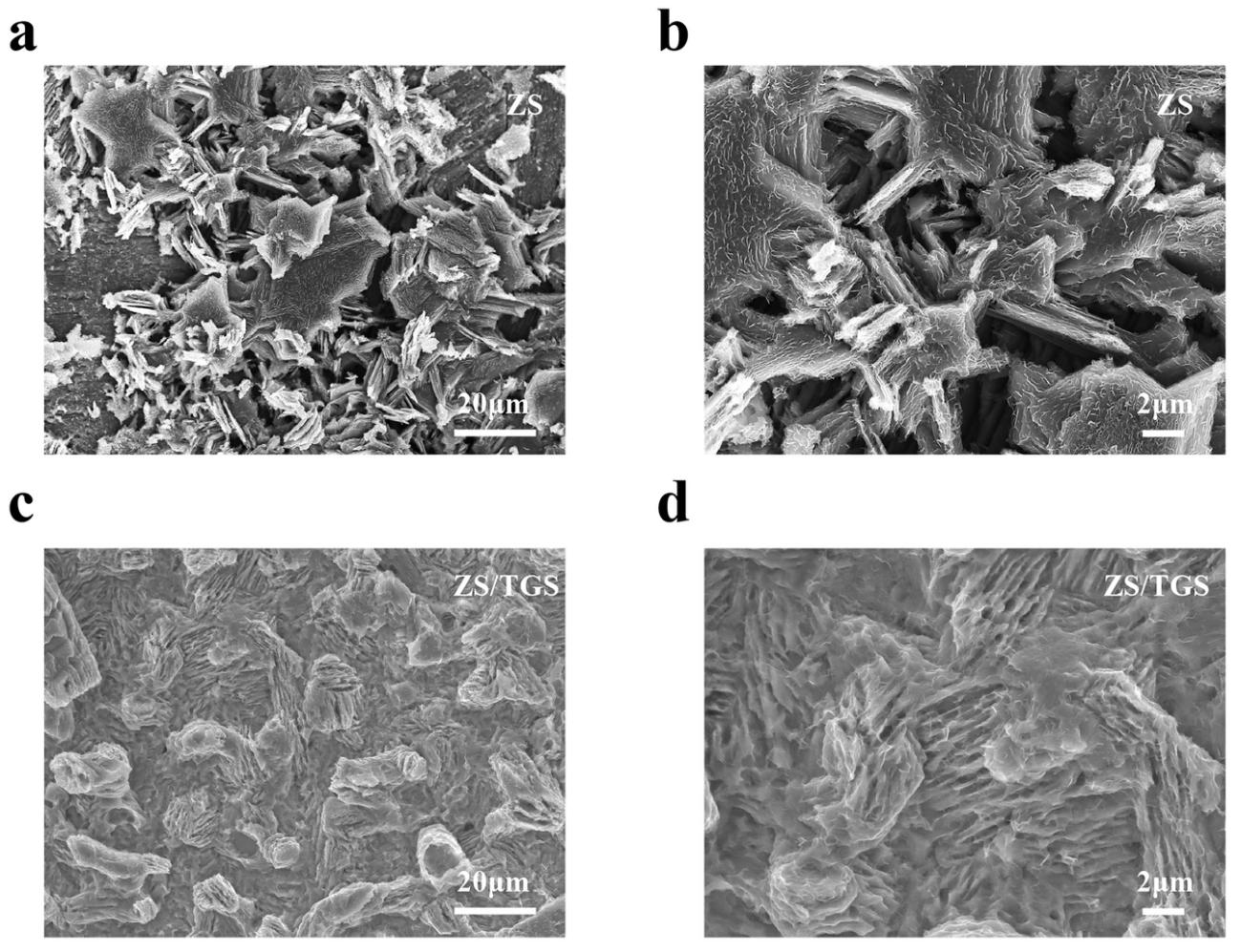
**

**Fig. S43** SEM images of cycled Zn foils after 30 cycles at a current density of 4 mA cm^−2^ and an areal capacity of 2 mAh cm^−2^ in **a, b** ZS and **c, d** ZS/TGS electrolytes


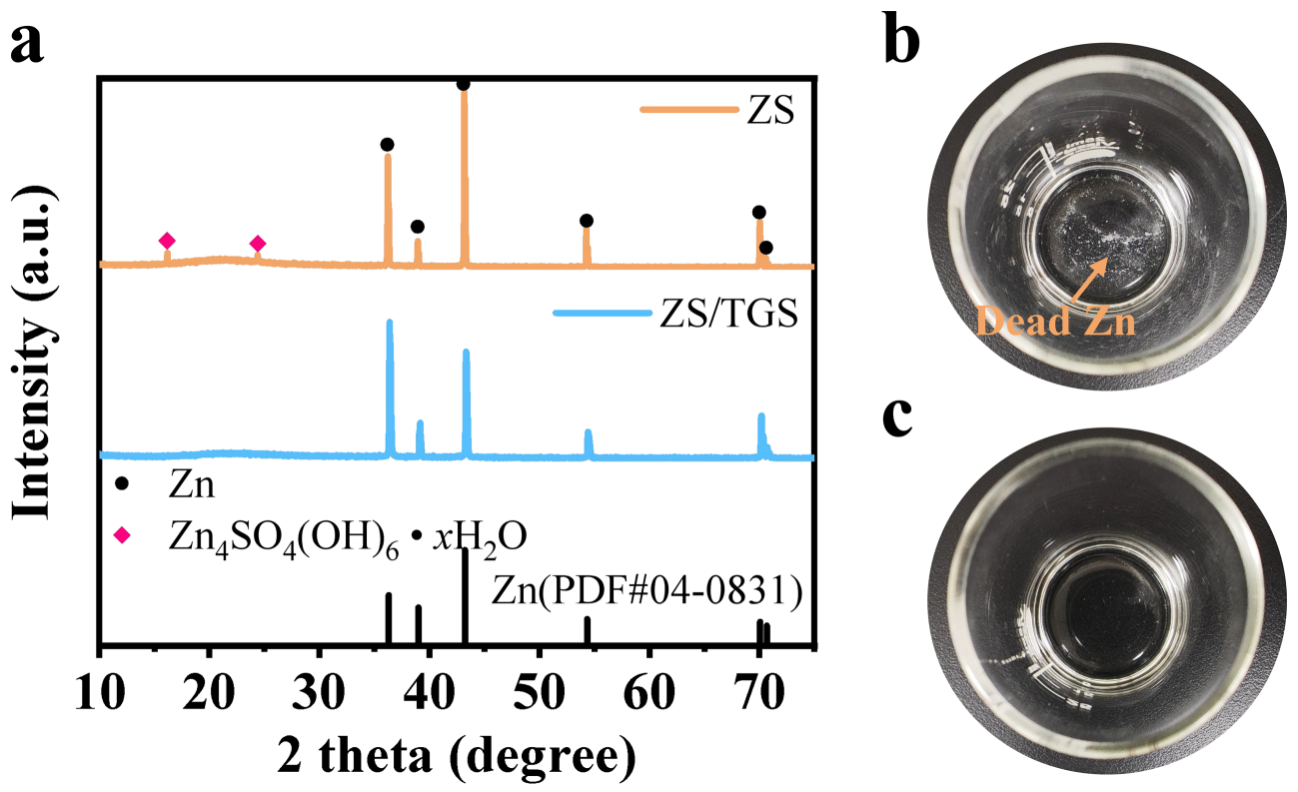


**Fig. S44** **a** X-ray diffraction (XRD) pattern of cycled Zn foils in ZS and ZS/TGS electrolytes after 30 cycles at a current density of 1 mA cm^−2^ and an areal capacity of 1 mAh cm^−2^. The ZS system shows peaks corresponding to Zn_4_SO_4_(OH)_6_∙*x*H_2_O (pink diamonds), indicating the formation of undesirable by-products, whereas the ZS/TGS system displays only Zn peaks. **b, c** Optical photographs of the cycled electrolytes in beaker cells reveal significant dead Zn accumulation in the ZS electrolyte **b**, while the ZS/TGS electrolyte **c** remains clear and transparent, further demonstrating the superior performance and reduced byproduct formation in the TGS-containing electrolyte


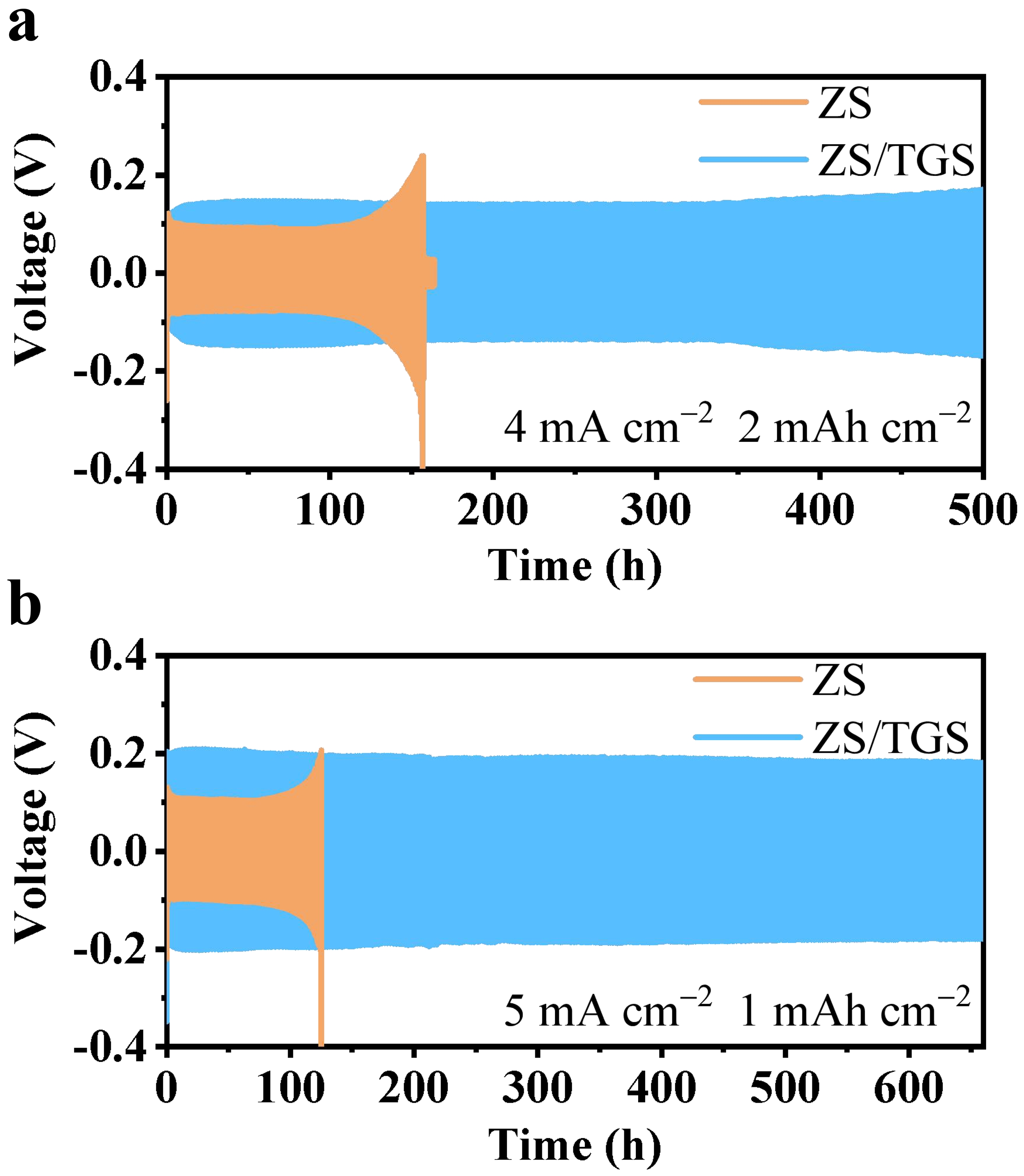


**Fig. S45** Voltage profiles of Zn||Zn symmetric cells during galvanostatic cycling in ZS and ZS/TGS electrolytes. **a** Cycling at a current density of 4 mA cm^−2^ and an areal capacity of 2 mAh cm^−2^. **b** Cycling at a current density of 5 mA cm^−2^ and an areal capacity of 1 mAh cm^−2^


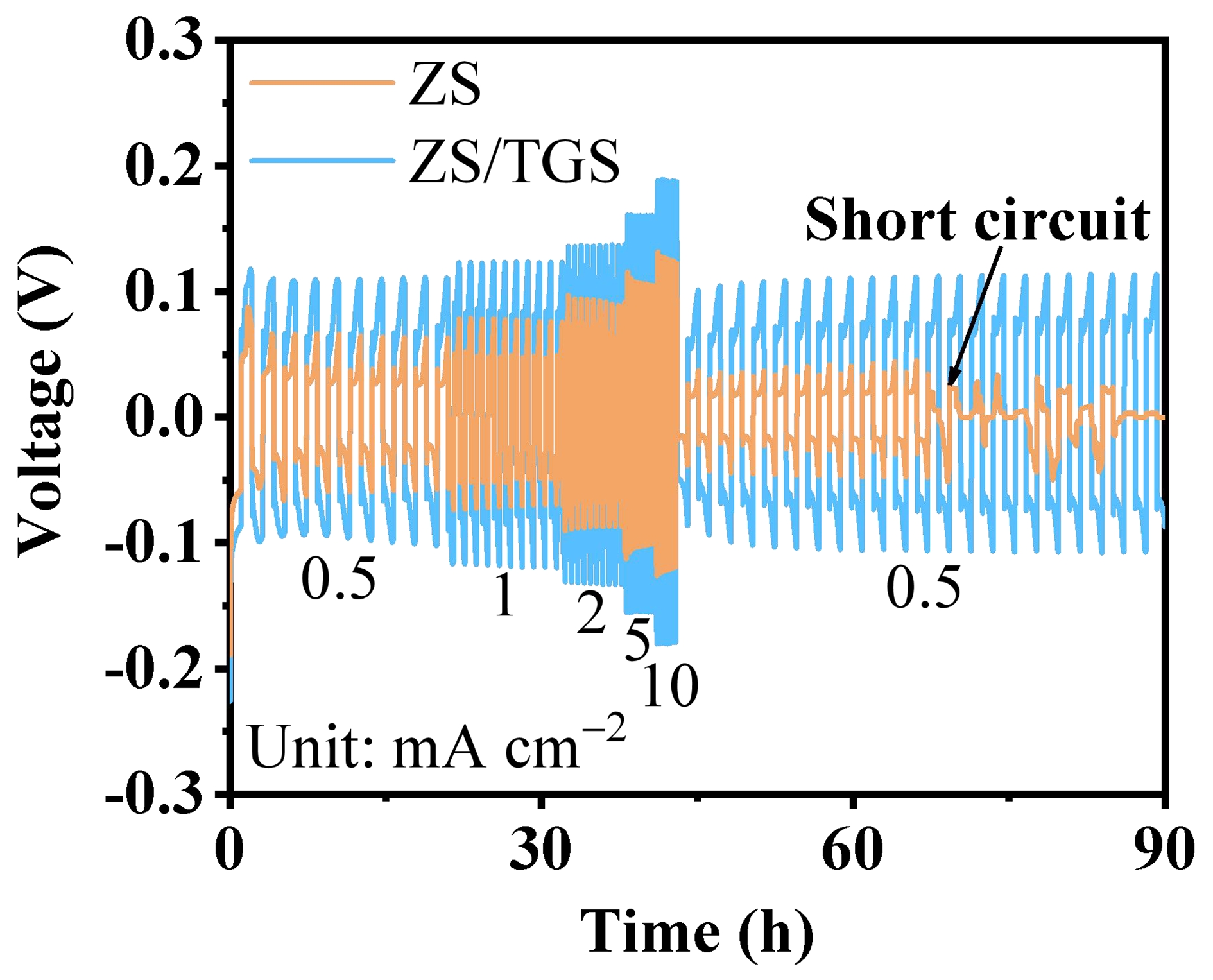


**Fig. S46** Rate performance of Zn||Zn symmetric cells with a fixed areal capacity of 0.5 mAh cm^–2^


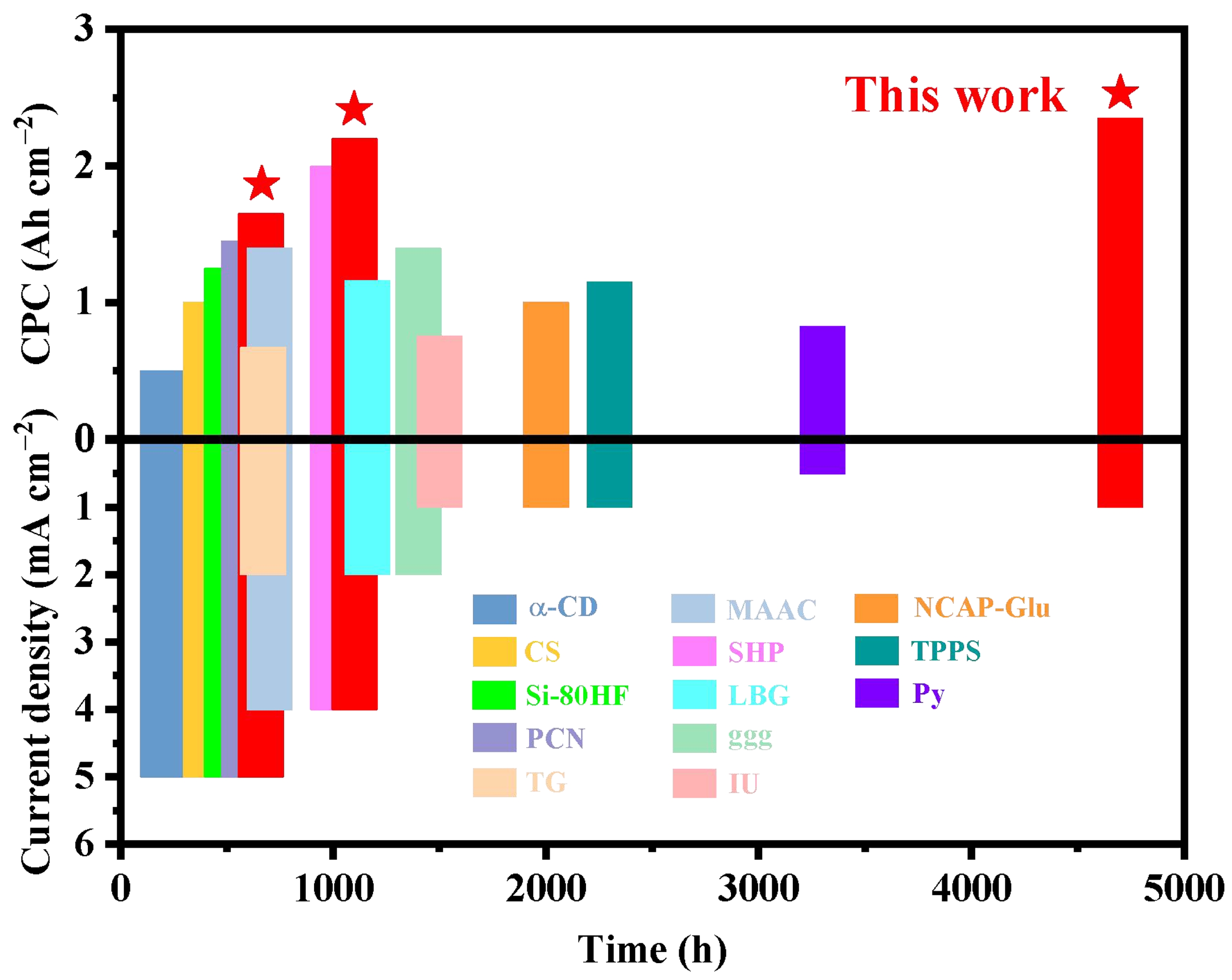


**Fig. S47** Comparative performance chart of various electrolyte additives strategies, illustrating cumulative plating capacity (CPC) versus cycling lifespan


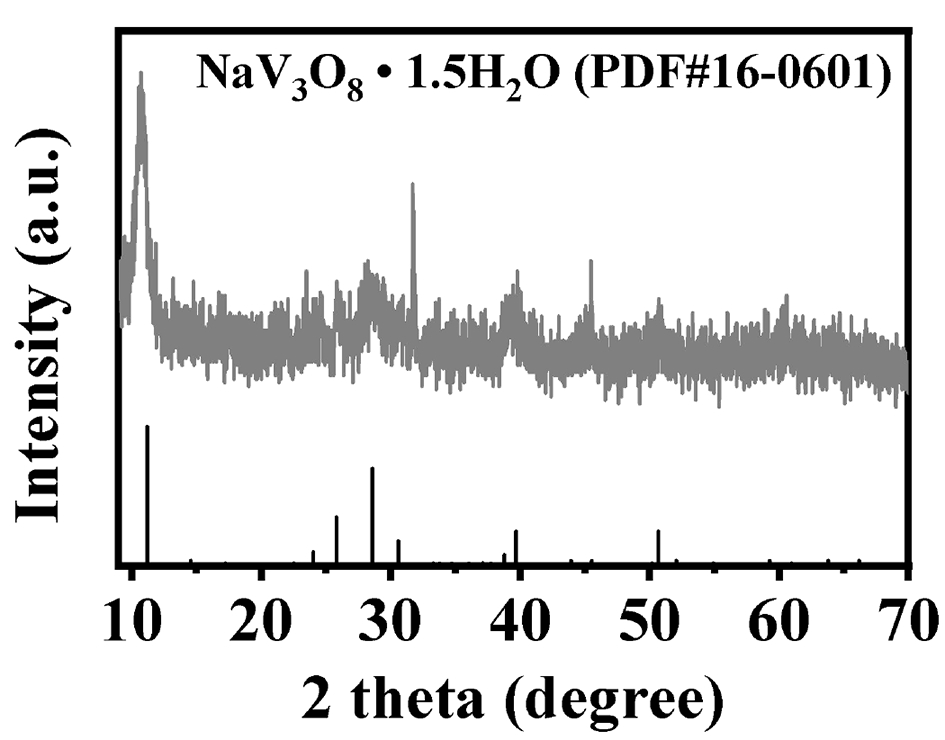


**Fig. S48** XRD pattern of the NVO cathode


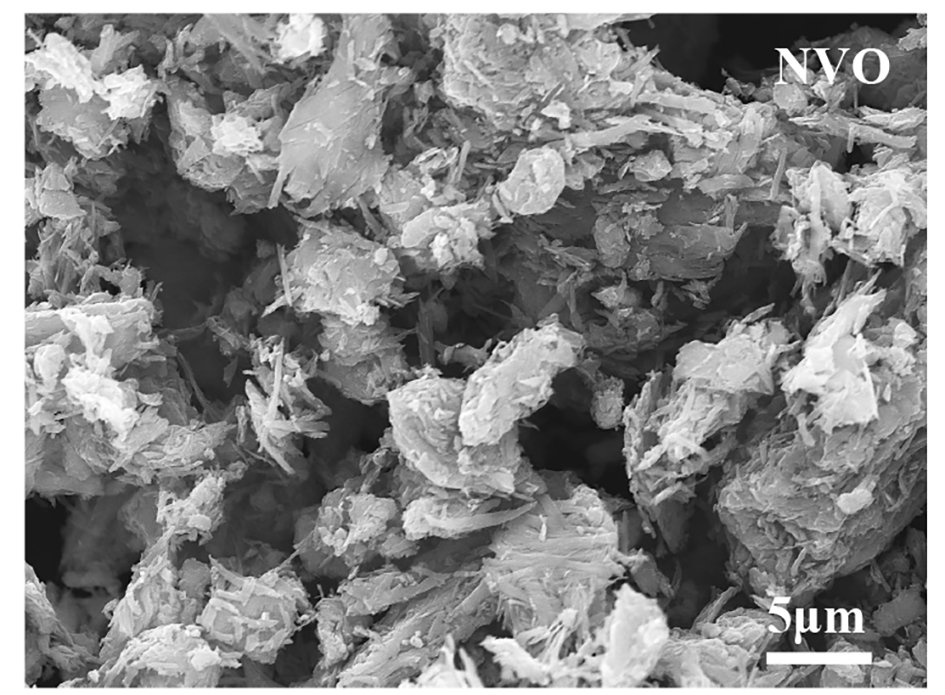


**Fig. S49** SEM image of the NVO cathode


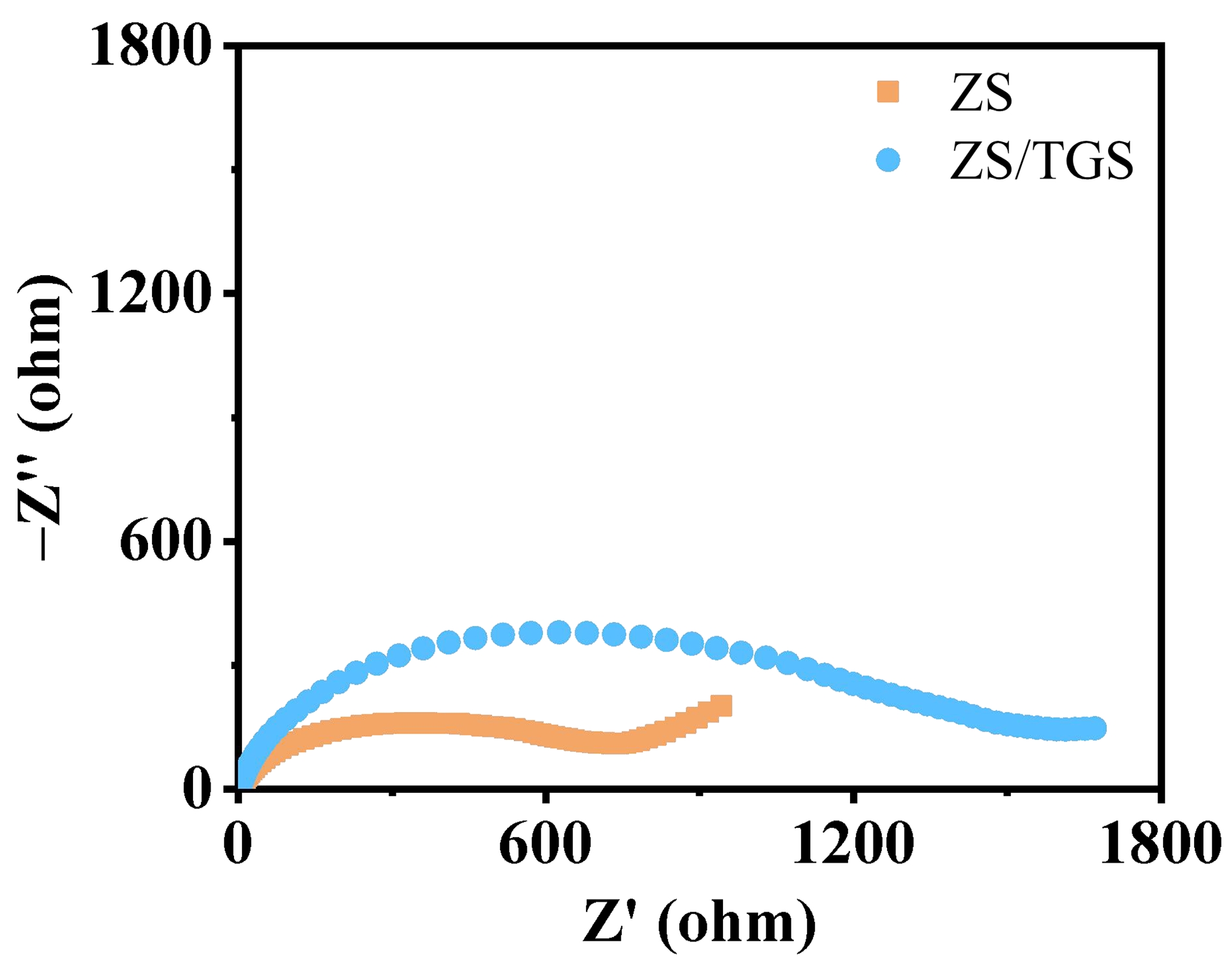


**Fig. S50** EIS spectra of Zn||NVO full cells in ZS and ZS/TGS electrolytes at 298.15 K


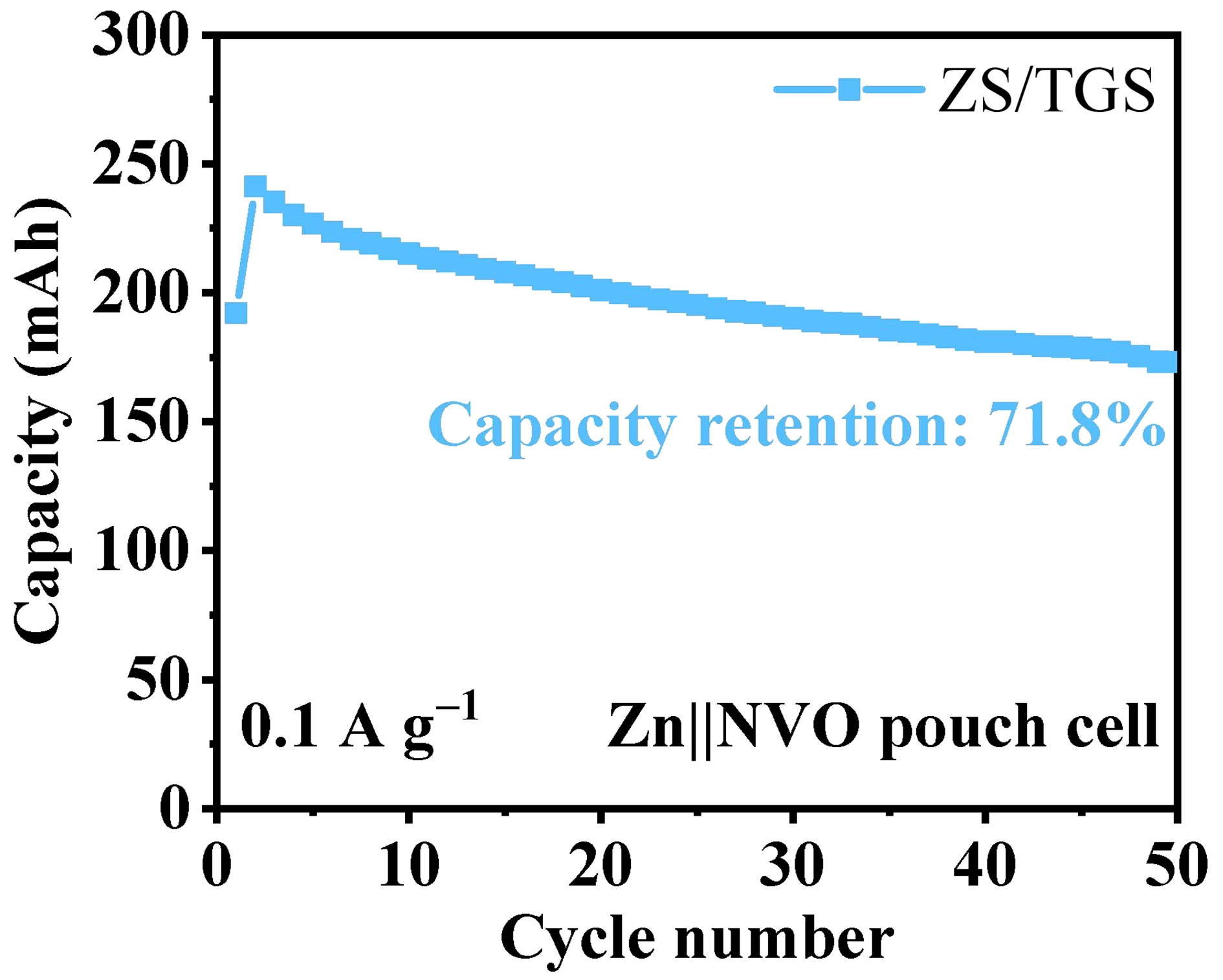


**Fig. S51** Long-term cycling performance of the Zn||NVO pouch cell with ZS/TGS electrolyte at 0.1 A g^−1^ (2 mA cm^−2^), exhibiting a capacity retention of 71.8% after 50 cycles

**S3 Supplementary Tables**

**Table S1** Minimum and maximum surface electrostatic potential points for TGS and H_2_O molecules, along with their corresponding nucleophilic and electrophilic sites

| Molecules | Index | Minimum/maximal surface electrostatic potential extrema (kcal mol^−1^) | Nucleophilic/electrophilic site |
| --- | --- | --- | --- |
| TGS | 1 | −39.2 | O |
|  | 2 | −38.83 | O |
|  | 3 | −37.55 | O |
|  | 4 | −35.35 | O |
|  | 5 | −71.83 | O |
|  | 6 | −25.3 | O |
|  | 7 | −17.15 | Cl |
| H_2_O | 1 | −44.1 | O |
|  | 2 | 54.3 | H |
|  | 3 | 54.2 | H |

**Table S2** Adsorption energy and adsorbate-surface angle for different adsorption configurations on the Zn (002) surface

| System | Adsorption sites | Total energy (eV) | Zn (002) slab energy (eV) | Adsorbate energy (eV) | Adsorption energy (eV) | Angle (degree) |
| --- | --- | --- | --- | --- | --- | --- |
| Zn (002) + TGS | Parallel-1 | −506.765 | −267.171 | −238.626 | −0.967 | 25.564 |
|  | Parallel-2 | −506.712 | −267.171 | −238.626 | −0.914 | 28.838 |
|  | Angle-1 | −506.545 | −267.171 | −238.626 | −0.747 | 37.530 |
|  | Angle-2 | −506.289 | −267.171 | −238.626 | −0.491 | 35.723 |
|  | Vertical-1 | −506.456 | −267.171 | −238.626 | −0.658 | 87.385 |
|  | Vertical-2 | −506.257 | −267.171 | −238.626 | −0.459 | 82.429 |
| Zn (002) + H_2_O | TOP | −281.639 | −267.171 | −14.228 | −0.239 | 14.499 |
|  | Bridge | −281.597 | −267.171 | −14.228 | −0.198 | 31.056 |
|  | HCP | −281.582 | −267.171 | −14.228 | −0.183 | 76.132 |
|  | FCC | −281.594 | −267.171 | −14.228 | −0.194 | 20.194 |

**Table S3** The composition and size of the bulk/interfacial simulation boxes

| Composition | H_2_O | Zn^2+^ | SO_4_^2−^ | TGS | Metal Zn atom | *x* (Å) | *y* (Å) | *z* (Å) |
| --- | --- | --- | --- | --- | --- | --- | --- | --- |
| Bulk ZnSO_4_ electrolyte | 4440 | 160 | 160 | 0 | 0 | 51.76 | 51.76 | 51.76 |
| Bulk ZnSO_4_/TGS electrolyte | 4440 | 160 | 160 | 40 | 0 | 53.71 | 53.71 | 53.71 |
| Interfacial ZnSO_4_ electrolyte | 4440 | 160 | 160 | 0 | 4032 | 55.12 | 55.69 | 128.96 |
| Interfacial ZnSO_4_/TGS electrolyte | 4440 | 160 | 160 | 40 | 4032 | 55.28 | 55.85 | 134.98 |

**Table S4** Comparison of electrochemical performance in Zn||Zn symmetric cells with other reported works

| **Additive** | **Current density**  **(mA cm^–2^)** | **Areal capacity**  **(mAh cm^–2^)** | **Cycle life**  **(hour)** | **CPC**  **(Ah cm^–2^)** | **References** |
| --- | --- | --- | --- | --- | --- |
| **TGS** | **1** | **1** | **4700** | **2.35** | **This work** |
|  | **4** | **1** | **1100** | **2.2** |  |
|  | **5** | **1** | **660** | **1.65** |  |
| TPPS | 1 | 1 | 2300 | 1.15 | [S8] |
| TG | 2 | 0.67 | 670 | 0.67 | [S9] |
| Py | 0.5 | 0.5 | 3300 | 0.825 | [S10] |
| Si-80HF | 5 | 1 | 500 | 1.25 | [S11] |
| MAAC | 4 | 4 | 700 | 1.4 | [S12] |
| α-CD | 5 | 5 | 200 | 0.5 | [S13] |
| IU | 1 | 0.5 | 1500 | 0.75 | [S14] |
| SHP | 4 | 2 | 1000 | 2 | [S15] |
| ggg | 2 | 2 | 1400 | 1.4 | [S16] |
| PCN | 5 | 5 | 580 | 1.45 | [S17] |
| CS | 5 | 5 | 400 | 1 | [S18] |
| LBG | 2 | 2 | 1160 | 1.16 | [S19] |
| NCAP-Glu | 1 | 1 | 2000 | 1 | [S20] |

**Supplementary References**

1. R. Sundararaman, K. Letchworth-Weaver, K.A. Schwarz, D. Gunceler, Y. Ozhabes et al., JDFTx: Software for joint density-functional theory. SoftwareX **6**, 278–284 (2017). <https://doi.org/10.1016/j.softx.2017.10.006>
2. K.F. Garrity, J.W. Bennett, K.M. Rabe, D. Vanderbilt, Pseudopotentials for high-throughput DFT calculations. Comput. Mater. Sci. **81**, 446–452 (2014). <https://doi.org/10.1016/j.commatsci.2013.08.053>
3. J. Perdew, K. Burke, M. Ernzerhof, Generalized gradient approximation made simple. Phys. Rev. Lett. **77**(18), 3865–3868 (1996). <https://doi.org/10.1103/PhysRevLett.77.3865>
4. R. Sundararaman, W.A. Goddard III, The charge-asymmetric nonlocally determined local-electric (CANDLE) solvation model. J. Chem. Phys. **142**(6), 064107 (2015). <https://doi.org/10.1063/1.4907731>
5. K. Letchworth-Weaver, T.A. Arias, Joint density functional theory of the electrode-electrolyte interface: Application to fixed electrode potentials, interfacial capacitances, and potentials of zero charge. Phys. Rev. B **86**(7), 075140 (2012). <https://doi.org/10.1103/physrevb.86.075140>
6. S.-Y. Lee, S.-H. Lee, J.-G. Park, Interaction forces between silica particles and wafer surfaces during chemical mechanical planarization of copper. J. Electrochem. Soc. **150**(5), G327 (2003). <https://doi.org/10.1149/1.1566417>
7. A.M. Smith, M. Borkovec, G. Trefalt, Forces between solid surfaces in aqueous electrolyte solutions. Adv. Colloid Interface Sci. **275**, 102078 (2020). <https://doi.org/10.1016/j.cis.2019.102078>
8. X. Zhao, Y. Wang, C. Huang, Y. Gao, M. Huang et al., Tetraphenylporphyrin-based chelating ligand additive as a molecular sieving interfacial barrier toward durable aqueous zinc metal batteries. Angew. Chem. Int. Ed. **62**(46), e202312193 (2023). <https://doi.org/10.1002/anie.202312193>
9. Z. Liu, R. Wang, Q. Ma, J. Wan, S. Zhang et al., A dual-functional organic electrolyte additive with regulating suitable overpotential for building highly reversible aqueous zinc ion batteries. Adv. Funct. Mater. **34**(5), 2214538 (2024). <https://doi.org/10.1002/adfm.202214538>
10. J. Luo, L. Xu, Y. Zhou, T. Yan, Y. Shao et al., Regulating the inner Helmholtz plane with a high donor additive for efficient anode reversibility in aqueous Zn-ion batteries. Angew. Chem. Int. Ed. **62**(21), e202302302 (2023). <https://doi.org/10.1002/anie.202302302>
11. H. Wu, W. Yan, Y. Xing, L. Li, J. Liu et al., Tailoring the interfacial electric field using silicon nanoparticles for stable zinc-ion batteries. Adv. Funct. Mater. **34**(5), 2213882 (2024). <https://doi.org/10.1002/adfm.202213882>
12. L. Zheng, H. Li, X. Wang, Z. Chen, C. Hu et al., Competitive solvation-induced interphases enable highly reversible Zn anodes. ACS Energy Lett. **8**(5), 2086–2096 (2023). <https://doi.org/10.1021/acsenergylett.3c00650>
13. K. Zhao, G. Fan, J. Liu, F. Liu, J. Li et al., Boosting the kinetics and stability of Zn anodes in aqueous electrolytes with supramolecular cyclodextrin additives. J. Am. Chem. Soc. **144**(25), 11129–11137 (2022). <https://doi.org/10.1021/jacs.2c00551>
14. X. Wang, Y. Ying, X. Li, S. Chen, G. Gao et al., Preferred planar crystal growth and uniform solid electrolyte interfaces enabled by anion receptors for stable aqueous Zn batteries. Energy Environ. Sci. **16**(10), 4572–4583 (2023). <https://doi.org/10.1039/D3EE01580G>
15. M. Chen, M. Yang, X. Han, J. Chen, P. Zhang et al., Suppressing rampant and vertical deposition of cathode intermediate product *via* pHregulation toward large-capacity and high-durability Zn// MnO_2_ batteries. Adv. Mater. **36**(4), 2304997 (2024). <https://doi.org/10.1002/adma.202304997>
16. J. Zhang, Y. Liu, Y. Wang, Z. Zhu, Z. Yang, Zwitterionic organic multifunctional additive stabilizes electrodes for reversible aqueous Zn-ion batteries. Adv. Funct. Mater. **34**(34), 2401889 (2024). <https://doi.org/10.1002/adfm.202401889>
17. J. Heo, Y. Roh, K. Shin, C. Lee, C. Wang et al., Versatile Zn hosting Lewis-basic interfacial layer for high-performant Zn reversibility in aqueous Zn ion battery. Energy Storage Mater. **71**, 103580 (2024). <https://doi.org/10.1016/j.ensm.2024.103580>
18. H. Cui, W. Li, H. Chen, Z. Liu, D. Zhou, Realizing dendrite-free Zn anode using an efficient sulfone-based electrolyte additive for high-performance aqueous zinc-ion batteries. J. Energy Chem. **109**, 455–465 (2025). <https://doi.org/10.1016/j.jechem.2025.05.058>
19. K. Wang, H. Zhan, W. Su, X.-X. Liu, X. Sun, Ordered interface regulation at Zn electrodes induced by trace gum additives for high-performance aqueous batteries. Energy Environ. Sci. **18**(3), 1398–1407 (2025). <https://doi.org/10.1039/D4EE04100C>
20. X. Fan, L. Chen, Y. Wang, X. Xu, X. Jiao et al., Selection of negative charged acidic polar additives to regulate electric double layer for stable zinc ion battery. Nanomicro Lett. **16**(1), 270 (2024). <https://doi.org/10.1007/s40820-024-01475-5>
